# Synthesis of *N*-phenyl- and *N*-thiazolyl-1*H*-indazoles by copper-catalyzed intramolecular *N*-arylation of *ortho*-chlorinated arylhydrazones

**DOI:** 10.3762/bjoc.18.110

**Published:** 2022-08-23

**Authors:** Yara Cristina Marchioro Barbosa, Guilherme Caneppele Paveglio, Claudio Martin Pereira de Pereira, Sidnei Moura, Cristiane Storck Schwalm, Gleison Antonio Casagrande, Lucas Pizzuti

**Affiliations:** 1 Grupo de Pesquisa em Síntese e Caracterização Molecular do Mato Grosso do Sul, Universidade Federal da Grande Dourados, 79804-970, Dourados-MS, Brazil; 2 Centro de Ciências Químicas, Farmacêuticas e de Alimentos, Laboratório de Lipidômica e Bioorgânica, Universidade Federal de Pelotas, Campus Universitário s/n, 96010-900, Capão do Leão-RS, Brazil; 3 Laboratório de Biotecnologia de Produtos Naturais e Sintéticos, Universidade de Caxias do Sul, 95070-560, Caxias do Sul-RS, Brazil

**Keywords:** fused-ring systems, hydrazones, indazoles, intramolecular cyclization, *N*-heterocycles

## Abstract

The broad application of 1*H*-indazoles has prompted the development of several approaches for the synthesis of such compounds, including metal-free, palladium-, or copper-promoted intramolecular *N*-arylation of in situ-generated or isolated *o*-haloarylhydrazones. Such methods mainly start from *o*-bromo derivatives due to the better yield observed when compared to those obtained from *o*-chloroarylhydrazones. However, the *o*-chloroarylaldehydes and *o*-chloroarylketones used to prepare the arylhydrazones are more commercially available and less expensive than brominated analogs. Seeking to cover a lack in the literature, this work reports a convenient protocol for the synthesis of *N*-phenyl- and *N*-thiazolyl-1*H*-indazoles by copper-catalyzed intramolecular *N*-arylation of *o*-chlorinated arylhydrazones. Therefore, a series of seven *N*-phenyl derivatives and a series of six novel *N*-thiazolyl derivatives was obtained in 10–70% and 12–35% yield, respectively, after stirring the *o*-chlorinated arylhydrazones, CuI, KOH, and 1,10-phenantroline for 12–48 hours in DMF at 120 °C. The products were isolated by column chromatography on silica gel. All products were fully characterized by HRMS as well as ^1^H and ^13^C NMR spectroscopy. Thus, this approach is valuable for promoting the synthesis of *N*-phenyl-1*H*-indazoles in a higher yield than that reported in the literature using copper catalysis and the same substrates. This study also prompted the first reported synthesis of pharmacologically interesting *N*-thiazolyl derivatives.

## Introduction

1*H*-Indazoles are important scaffolds due to the prevalence in compounds with biological activity [1], such as anticancer [2], anti-HIV [3], anti-inflammatory [4], antiprotozoal [5], antifungal [6], antibacterial [7], antiplatelet [8], and antihypertensive [9] properties. The relevance to medicinal chemistry is also demonstrated by the presence of the 1*H*-indazole core in the structure of drugs. The anticataract agent bendazac [10], the anti-inflammatory agent benzydamine [11], and the antiemetic agent granisetron [12] are commercially available examples.

In view of the abovementioned interest, an increasing number of approaches for the synthesis of 1*H*-indazoles has been recently reported, including 1,3-dipolar cycloaddition reaction of α-diazomethylphosphonates with *o*-(trimethylsilyl)phenyl triflate in the presence of CsF [13], Cu^2+^-mediated N−N bond formation from ketimines in the presence of oxygen [14], Pd^2+^-mediated oxidative benzannulation from pyrazoles and internal alkynes [15], Pd-catalyzed Aza–Nenitzescu reaction of hydrazones and *p*-benzoquinones [16], and Co^3+^/Cu^2+^-catalyzed C−N/N−N coupling of imidates with anthranils as both aminating reagents and organic oxidants [17]. Additional established routes to 1*H*-indazoles comprise transition metal‐catalyzed [18–20] and metal-free [21–22] intramolecular amination by oxidative C−H bond functionalizations. These methods showed significant improvement with respect to the substrate scope and reaction conditions. However, mostly they are restricted to substrates containing hydrogen [13–14], alkyl [14–15], or (substituted) phenyl moieties [14,16–17] as *N*‐substituents, and others suffer from poor regioselectivity for substrates without a directing group [18–19,21–22]. On the other hand, methods based on metal-free [23], palladium- [24–25], and copper-promoted [26–31] intramolecular *N*-arylation of in situ-generated or isolated *o*-haloarylhydrazones are advantageous concerning regioselectivity. However, they also present limitations, such as costly Pd catalysts, a scope of the *N*-substituent limited to alkyl [30], (substituted) phenyl [23–29], and tosyl moieties [31], and low to moderate yield when *o*-chloroarylaldehydes or *o*-chloroarylketones are the starting material [27–28]. The preference for the use of *o*-bromo analogues is due to to the better yield obtained compared to using *o*-chloroarylhydrazones. However, the availability of commercial *o*-chloroarylaldehydes and *o*-chloroaryl ketones is greater than that of brominated analogs, and the cost of the former is significantly advantageous, even when the lower yield is considered.

Since the cost and availability of the starting material are crucial issues during the synthetic planning, we envisioned that it would be possible to employ *o*-chlorobenzaldehydes as starting material for the synthesis of *N*-phenyl-1*H*-indazoles if the reaction conditions are carefully chosen. In addition, another target of the work was the application of the optimized reaction conditions to the cyclization of thiazolylhydrazones, aiming for the synthesis of novel *N*-thiazolyl-1*H*-indazoles.

## Results and Discussion

First, optimization of the reaction conditions was conducted using substrate **1a** as a model according to Table 1. The molar ratio of **1a**, the catalyst, the base, and the ligand used in Table 1, entry 1 was adapted from the literature [25]. The reaction mixture was stirred for 5 h at 120 °C, which gave the product **2a** in 32% yield. Increasing the time to 12 h led to a better yield of 40% (Table 1, entry 2). The best yield (60%) was reached after 24 h (Table 1, entry 3). The reduction of the temperature to 100 °C drastically decreased the yield of **2a** (Table 1, entry 4). The solvent effect on the reaction yield was evaluated employing other aprotic solvents, but no improvement was observed compared to when DMF was used (Table 1, entries 5–7). When the reaction was carried out in NMP (*N*-methyl-2-pyrrolidone), the ^1^H NMR spectrum of the crude mixture obtained after extraction did not show the presence of the product **2a**, but it showed the presence of 91% of the starting hydrazone (Table 1, entry 8). To evaluate the effect of the base, K_3_PO_4_ or Cs_2_CO_3_ was used, but the yield was lower than 60% (Table 1, entries 9 and 10). The influence of the catalyst/ligand molar ratio (Table 1, entries 11 and 12) showed that in both cases, the yield was not better than that in Table 1, entry 3. Further, the increase in the reaction concentration from 0.2 M to 0.4 M did not allow for better results (Table 1, entry 13). The copper source is a less explored aspect, considering previous literature on the synthesis of 1*H*-indazoles. As such, this work devoted great attention to the nature of the catalyst and evaluated several copper sources other than CuI, such as CuBr, CuCl, CuO, and Cu^0^ in powder form (Table 1, entries 14–17). Among them, CuI provided the best performance. Finally, *N*,*N*'-dimethylethanolamine (DMEA) and *trans*-1,2-diaminocyclohexane (DACH) were evaluated as ligands (Table 1, entries 18 and 19). However, the yield was lower compared to that obtained with 1,10-phenanthroline (phen). Control experiments were performed without catalyst (Table 1, entry 20), base (Table 1, entry 21), or ligand (Table 1, entry 22), but the product could not be detected by TLC analysis.

**Table 1:** Optimization of the reaction conditions for the synthesis of 1-phenyl-1*H*-indazole (**2a**).^a^

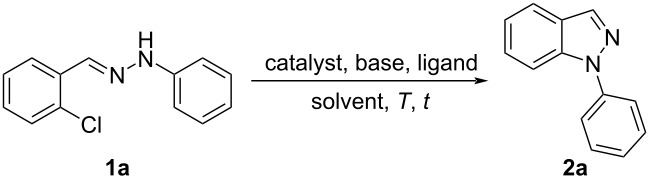							

entry	catalyst	base	ligand	solvent	*T* (°C)	*t* (h)	yield (%)^b^

1	CuI	KOH	phen	DMF	120	5	32
2	CuI	KOH	phen	DMF	120	12	40
3	CuI	KOH	phen	DMF	120	24	60
4	CuI	KOH	phen	DMF	100	24	—^c^
5	CuI	KOH	phen	DMSO	120	24	27
6	CuI	KOH	phen	dioxane	120	24	42
7	CuI	KOH	phen	toluene	120	24	46
8	CuI	KOH	phen	NMP	120	24	—^d^
9	CuI	K_3_PO_4_	phen	DMF	120	24	40
10	CuI	Cs_2_CO_3_	phen	DMF	120	24	26
11	CuI	KOH	phen^e^	DMF	120	24	39
12	CuI	KOH	phen^f^	DMF	120	24	47
13	CuI	KOH	phen	DMF^g^	120	24	57
14	CuCl	KOH	phen	DMF	120	24	47
15	CuBr	KOH	phen	DMF	120	24	50
16	CuO	KOH	phen	DMF	120	24	34
17	Cu^0^	KOH	phen	DMF	120	24	36
18	CuI	KOH	DMEA	DMF	120	24	36
19	CuI	KOH	DACH	DMF	120	24	29
20	—	KOH	phen	DMF	120	24	—^h^
21	CuI	—	phen	DMF	120	24	—^h^
22	CuI	KOH	—	DMF	120	24	—^h^

^a^Unless otherwise noted, the reactions were carried out with **1a** (0.5 mmol), catalyst (20 mol %), base (200 mol %), ligand (22 mol %), and solvent (2.5 mL) and heated at 120 °C for the indicated time. ^b^Isolated yield. ^c^≈23% of the product **2a** and ≈53% of the starting hydrazone **1a** were detected by ^1^H NMR spectroscopy (Figures S1 and S2, Supporting Information File 1). ^d^No product and ≈91% of the starting hydrazone **1a** were detected by ^1^H NMR (Figures S3 and S4, Supporting Information File 1). ^e^40 mol % of phen was used. ^f^20 mol % of phen and 10 mol % of CuI were used. ^g^1.25 mL of DMF (0.4 M) was used. ^h^Product **2a** was not detected by TLC analysis. phen = 1,10-phenanthroline, NMP = *N*-methyl-2-pyrrolidone, DMEA = *N*,*N’*-dimethylethanolamine, DACH = *trans*-1,2-diaminocyclohexane.

**Scheme 1:**
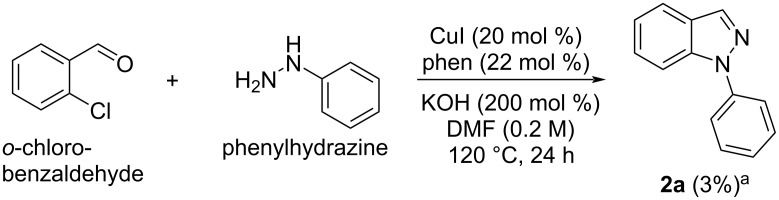
One-pot approach for the synthesis of **2a**. ^a^Yield calculated vs trichloroethylene by ^1^H NMR spectroscopy.

In order to compare the efficiency of the optimized conditions with the one-pot approach, a reaction was carried out starting from *o*-chlorobenzaldehyde and phenylhydrazine under the conditions shown in Table 1, entry 3 (Scheme 1). In the ^1^H NMR spectrum of the crude reaction mixture, only 3% of the desired product **2a** was detected, together with an undefined amount of the intermediate hydrazone **1a** (Figures S5 and S6, Supporting Information File 1).

Next, the most effective conditions as determined from the optimization were applied to prepare a series of seven *N*-phenyl-1*H*-indazole derivatives, **2a**,**b**,**d**–**h**, from *o*-chlorinated arylhydrazones **1a**,**b**,**d**–**h**. The structure of the starting materials and products as well as the reaction conditions and yields are shown in Table 2. In general, the reactions were highly impacted by the structure of the starting materials. The best yield was obtained from the unsubstituted arylhydrazones **1a** (60%) and **1h** (70%). On the other hand, the method was inefficient in converting arylhydrazones containing electron-donating groups at the 4-position. Accordingly, the reaction of 4-methyl-substituted arylhydrazone **1b** led to **2b** in only 10% yield and 4-methoxy-substituted arylhydrazone **1c** was not converted to the desired 1*H*-indazole **2c**. Arylhydrazones **1d**–**f** substituted with halogen atoms were converted in 23–53% yield, but for the 4-fluoro- and 5-fluoro-substituted substrates **1d** and **1f**, a longer reaction time was needed in order to increase the rather low yield observed after 24 h. In turn, to obtain the product **2g** derived from 5-nitro-substituted arylhydrazone **1g**, the reaction time had to be decreased.

**Table 2:** Selected experimental data for the synthesis of *N*-phenyl-1*H*-indazoles **2a**,**b**,**d**–**h**.

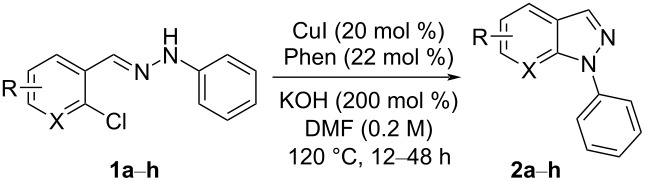			

hydrazone **1**	product **2**	*t* (h)	yield (%)^a^

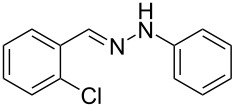 **1a**	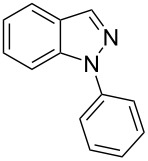 **2a**	24	60
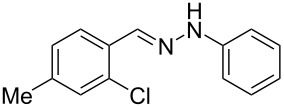 **1b**	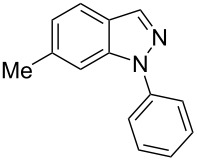 **2b**	24	10
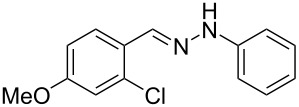 **1c**	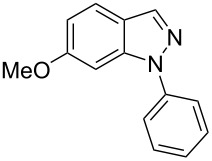 **2c**	24	—^b^
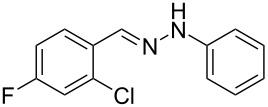 **1d**	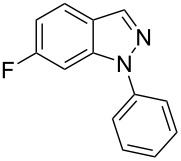 **2d**	39	23
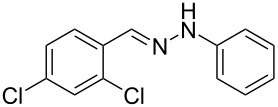 **1e**	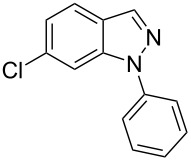 **2e**	24	39
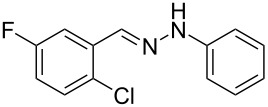 **1f**	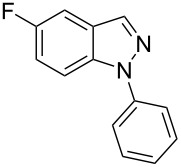 **2f**	48	53
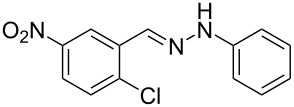 **1g**	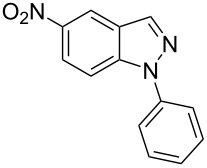 **2g**	12	16
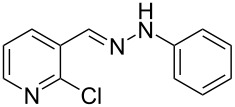 **1h**	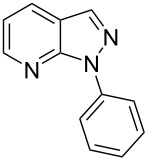 **2h**	24	70

^a^Isolated yield. ^b^Product not obtained.

Aiming to expand the scope of 1*H*-indazoles synthetized by copper-catalyzed intramolecular *N*-arylation of arylhydrazones, which was limited to derivatives with alkyl, (substituted) phenyl, and tosyl *N*-substituents, as detailed in the Introduction section, the same conditions were applied to convert *N*-thiazolyl-substituted arylhydrazones **3a**–**h** to the corresponding *N*-thiazolyl-1*H*-indazoles **4a**–**h**. Unfortunately, the conditions were not suitable to convert arylhydrazones containing an electron-donating group at the 3-position, such as Me in **3b** and OMe in **3c**. In general, the yield was lower than for the products in the *N*-phenyl-1*H*-indazole series. The structure of the starting materials impacted the yield of the products in a similar manner as observed for the previous series. Products **4a** and **4h** were obtained with the best yield of 35% and 34%, respectively. The yield of products **4d**–**g** ranged from 12–24%. Table 3 shows the structures of the starting materials and products as well as the reaction conditions and yields.

**Table 3:** Selected experimental data for the synthesis of *N*-thiazolyl-1*H*-indazoles **4a**,**d**–**h**.

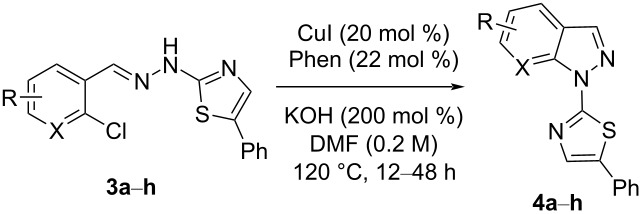			

hydrazone **3**	product **4**	*t* (h)	yield (%)^a^

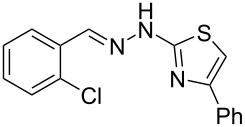 **3a**	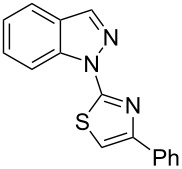 **4a**	24	35
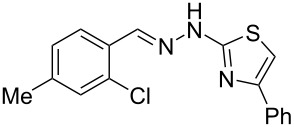 **3b**	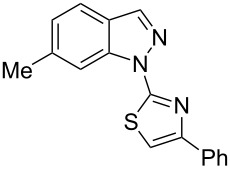 **4b**	24	—^b^
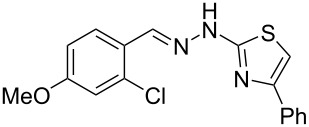 **3c**	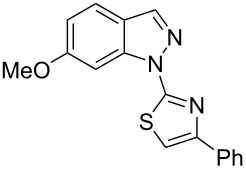 **4c**	48	—^b^
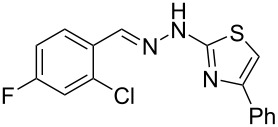 **3d**	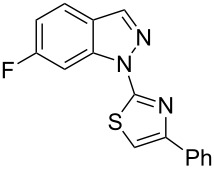 **4d**	24	12
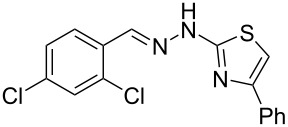 **3e**	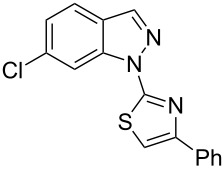 **4e**	24	23
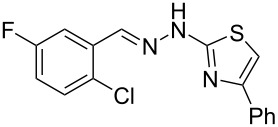 **3f**	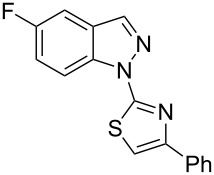 **4f**	24	24
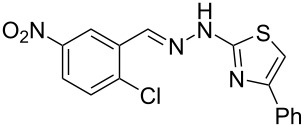 **3g**	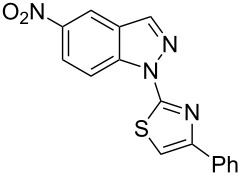 **4g**	12	19
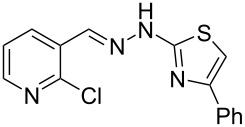 **3h**	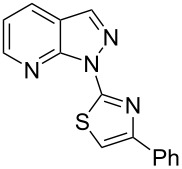 **4h**	24	34

^a^Isolated yield. ^b^Product not obtained.

**Scheme 2:**
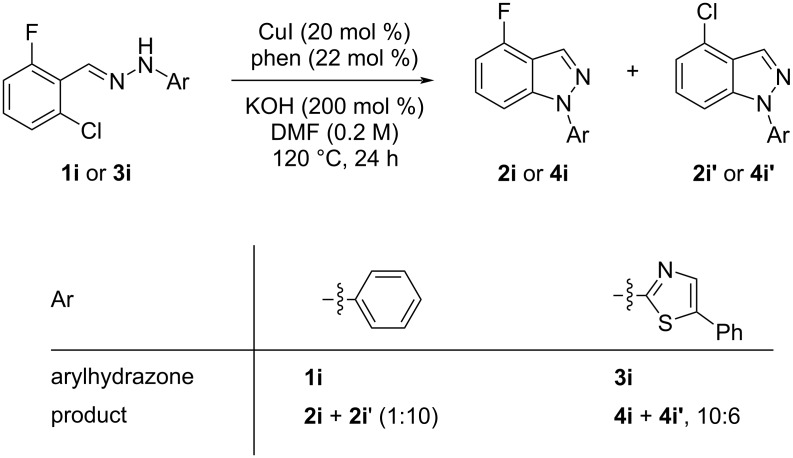
Regioselectivity of the reaction of arylhydrazones **1i** and **3i**, respectively.

When arylhydrazone **1i** or **3i**, respectively, was the starting material, competition between attack of the nitrogen atom on position 2 (C−Cl) and 6 (C−F) was observed (Scheme 2). The analysis of the ^1^H NMR (Figure S31, Supporting Information File 1), ^13^C NMR (Figure S32, Supporting Information File 1), and HRMS spectra (Figures S63 and S64, Supporting Information File 1) of the product obtained from *N*-phenylhydrazone **1i** showed a mixture of **2i** and **2i’**, with **2i’** being the main product, in the ratio 10:1, as calculated from the ^1^H NMR spectrum. The starting material **3i** also afforded a mixture of **4i** and **4i’**, as concluded from the analysis of the ^1^H NMR (Figure S45, Supporting Information File 1), ^13^C NMR (Figure S46, Supporting Information File 1), and HRMS spectra (Figures S79 and S80, Supporting Information File 1). However, **4i** was detected as the major product in the ratio 10:6, as seen in the ^1^H NMR spectrum. This inversion of the regioselectivity can be attributed to electronic and steric effects of the *N*-substituent but needs additional investigation for full comprehension. When arylhydrazone **1i** or **3i**, respectively, was subjected to the same reaction conditions in the absence of CuI, only the product **2i’** or **4i’**, respectively, was detected by ^1^H NMR spectroscopy in the crude reaction mixture (Figures S7–S10 and Figures S11–S14, respectively, Supporting Information File 1). This result shows that *o*-fluorinated arylhydrazones may be suitable as starting materials to prepare the title compounds by a S_N_Ar approach.

## Conclusion

A novel set of conditions to convert *o*-chlorinated arylhydrazones into 1*H*-indazoles by a copper-catalyzed intramolecular *N*-arylation approach has been determined. A series of seven *N*-phenyl-1*H*-indazoles was obtained in 10–70% yield. Although this yield is lower than that reached using *o*-brominated arylhydrazones by methods described in the literature, they surpass the yield obtained from the same substrates using copper catalysis. This approach also prompted the synthesis of six novel and interesting *N*-thiazolyl-1*H*-indazoles in 12–35% yield. Despite the lower yield, this is the first reported method for the preparation of such compounds. In summary, *N*-phenyl- and *N*-thiazolyl-1*H*-indazoles were conveniently obtained from the less expensive and more available *o*-chlorinated arylhydrazones.

## Experimental

### General

All chemicals were purchased from Sigma-Aldrich or Oakwood Chemicals and were used as supplied without further purification. The solvents were purchased from Synth. DMF was distilled over 4 Å molecular sieves and degassed by sparging with nitrogen for 30 min before use. Reactions for the synthesis of 1*H*-indazoles were performed in a resealable screw-cap Schlenk flask (≈10 mL volume) in the presence of a Teflon-coated magnetic stirring bar. TLC analyses were performed with Al plates covered with silica gel (SiliCycle, F_254_) and visualized by UV detection. Silica gel (Vetec, 0.063–0.200 mm, 70–230 mesh) was used for column chromatography. Melting points were determined with a PMF-II MS Tecnopon melting point apparatus using open capillaries. ^1^H (300 or 500 MHz) and ^13^C (75 or 126 MHz) NMR spectra were recorded at 25 °C with a Bruker Avance III HD or Ascend 500 spectrometer, respectively. The samples were dissolved in CDCl_3_/TMS or DMSO-*d*_6_. Chemical shifts (δ) were recorded in ppm. ^1^H NMR spectra recorded in CDCl_3_/TMS were calibrated using the TMS peak, and those recorded in DMSO-*d*_6_ were calibrated using the DMSO-*d*_5_ peak. ^13^C NMR spectra recorded in CDCl_3_/TMS and DMSO-*d*_6_ were calibrated using the CDCl_3_ and DMSO-*d*_6_ peak, respectively. The determination of the amount of product and/or starting material in the crude mixture by ^1^H NMR spectroscopy was conducted by adding 1 equiv of trichloroethylene as calibration compound after diluting the crude mixture in CDCl_3_. The amounts were estimated by comparing the integral value of the product C3–H signal at 8.21 ppm and the hydrazone triplet at 6.89 ppm, respectively, vs the integral value of the calibration compound singlet at 6.46 ppm (see Supporting Information File 1). HRMS spectra were acquired on a hybrid high-resolution and high-accuracy microTof (Q-TOF, Bruker Scientific) spectrometer with electrospray ionization (ESI) source (MicrOTOF-QII, Bruker Scientific) in positive mode. The compounds were individually dissolved in a solution of 50% chromatographic grade MeCN and 50% deionized H_2_O + 0.1% formic acid.

### General experimental procedure for the synthesis of hydrazones **1a–i**

In a 100 mL round-bottom flask, the *o*-chlorinated aromatic aldehyde (5 mmol) and phenylhydrazine (0.540 g, 5 mmol) were dissolved in methanol (25 mL). Glacial acetic acid (0.060 g, 20 mol %) and sodium acetate (0.082 g, 20 mol %) were added, and the solution was stirred for 4 h at room temperature. The resulting mixture was cooled in an ice bath and cold water (30 mL) was added to give a precipitate. In a Büchner funnel, the solid was filtered off and washed with an additional amount of cold water. After drying the solid in a desiccator, it was recrystallized from methanol/water 1:1 to give the products **1a**–**i** with good purity. The identity of the compounds **1a**–**i** was confirmed by HRMS analysis (see Supporting Information File 1).

### General experimental procedure for the synthesis of indazoles **2a,b,d–i,i’**

To a dried Schlenk tube, an arylhydrazone **1a**–**i** (0.5 mmol), KOH (0.056 g, 200 mol %), phen (0.020 g, 22 mol %), CuI (0.019 g, 20 mol %), and DMF (2.5 mL) were successively added under N_2_ atmosphere. The reaction mixture was stirred and heated at 120 °C for 12–48 h (see Table 1). After cooling to room temperature, AcOEt (10 mL) was added to the mixture. This was passed through a short column with silica gel 60, and then the eluate was washed with water (1 × 10 mL) and a saturated aqueous NaCl solution (2 × 10 mL). The resulting organic solution was dried over anhydrous MgSO_4_, filtered, and concentrated under reduced pressure. Each product **2a**,**b**,**d**–**i**,**i’** was isolated using column chromatography with silica gel 60 using the following eluent: hexane/AcOEt 98:2 for **2a**,**b**,**d**,**e**, petroleum ether/AcOEt 98:2 for **2f**, hexane/AcOEt 90:10 for **2g**,**i**,**i’**, and hexane/AcOEt 80:20 for **2h**.

### General experimental procedure for the synthesis of hydrazones **3a–i**

The *o*-chlorinated aromatic aldehyde (1.5 mmol), thiosemicarbazide (0.136 g, 1.5 mmol), 2-bromoacetophenone (0.298 g, 1.5 mmol), and absolute ethanol (2 mL) were added to a 10 mL round bottom flask. The mixture was stirred at room temperature for 5–10 min. The solid was filtered off, washed with ethyl ether, and dried in a desiccator to give the product **3a**–**i** with adequate purity to be used without further purification. The identity of the compounds **3a**–**i** was confirmed by HRMS analysis (see Supporting Information File 1).

### General experimental procedure for the synthesis of indazoles **4a,d–i,i’**

Each arylhydrazone **3a**–**i** was allowed to react following the same procedure reported above for **1a**–**i**. The reaction time ranged from 12 to 48 h (see Table 2). Each product **4a**,**d**–**i**,**i’** was isolated by column chromatography with silica gel 60 using the following eluent: hexane/AcOEt 90:10 for **4a**, petroleum ether/AcOEt 95:5 for **4d**, petroleum ether/AcOEt 98:2 for **4e**, hexane/AcOEt 95:5 for **4f**, hexane/AcOEt 80:20 for **4g**, hexane/AcOEt 98:2 for **4h**, and hexane/AcOEt 85:15 for **4i** and **4i’**.

## Supporting Information

File 1 Reaction analysis by ^1^H and ^13^C NMR spectroscopy, characterization data, NMR spectra for the 1*H*-indazoles and HRMS spectra for the arylhydrazones and 1*H*-indazoles.

## References

[1] Zhang S-G, Liang C-G, Zhang W-H (2018). Molecules.

[2] Dong J, Zhang Q, Wang Z, Huang G, Li S (2018). ChemMedChem.

[3] Kim S-H, Markovitz B, Trovato R, Murphy B R, Austin H, Willardsen A J, Baichwal V, Morham S, Bajji A (2013). Bioorg Med Chem Lett.

[4] Cheekavolu C, Muniappan M (2016). J Clin Diagn Res.

[5] Gerpe A, Aguirre G, Boiani L, Cerecetto H, González M, Olea-Azar C, Rigol C, Maya J D, Morello A, Piro O E (2006). Bioorg Med Chem.

[6] Rodríguez-Villar K, Hernández-Campos A, Yépez-Mulia L, Sainz-Espuñes T d R, Soria-Arteche O, Palacios-Espinosa J F, Cortés-Benítez F, Leyte-Lugo M, Varela-Petrissans B, Quintana-Salazar E A (2021). Pharmaceuticals.

[7] Reddy G S, Viswanath I V K, Rao A T (2018). Indian J Heterocycl Chem.

[8] Chen H-S, Kuo S-C, Teng C-M, Lee F-Y, Wang J-P, Lee Y-C, Kuo C-W, Huang C-C, Wu C-C, Huang L-J (2008). Bioorg Med Chem.

[9] Goodman K B, Cui H, Dowdell S E, Gaitanopoulos D E, Ivy R L, Sehon C A, Stavenger R A, Wang G Z, Viet A Q, Xu W (2007). J Med Chem.

[10] Heruye S H, Maffofou Nkenyi L N, Singh N U, Yalzadeh D, Ngele K K, Njie-Mbye Y-F, Ohia S E, Opere C A (2020). Pharmaceuticals.

[11] Quane P A, Graham G G, Ziegler J B (1998). Inflammopharmacology.

[12] Yarker Y E, McTavish D (1994). Drugs.

[13] Chen G, Hu M, Peng Y (2018). J Org Chem.

[14] Chen C-y, Tang G, He F, Wang Z, Jing H, Faessler R (2016). Org Lett.

[15] Kim O S, Jang J H, Kim H T, Han S J, Tsui G C, Joo J M (2017). Org Lett.

[16] Janardhanan J C, Mishra R K, Das G, Sini S, Jayamurthy P, Suresh C H, Praveen V K, Manoj N, Babu B P (2018). Asian J Org Chem.

[17] Li L, Wang H, Yu S, Yang X, Li X (2016). Org Lett.

[18] Inamoto K, Saito T, Katsuno M, Sakamoto T, Hiroya K (2007). Org Lett.

[19] Li X, He L, Chen H, Wu W, Jiang H (2013). J Org Chem.

[20] Park A, Jeong K-S, Lee H, Kim H (2021). ACS Omega.

[21] Wei W, Wang Z, Yang X, Yu W, Chang J (2017). Adv Synth Catal.

[22] Zhang Z, Huang Y, Huang G, Zhang G, Liu Q (2017). J Heterocycl Chem.

[23] Annor-Gyamfi J K, Gnanasekaran K K, Bunce R A (2018). Molecules.

[24] Cho C S, Lim D K, Heo N H, Kim T-J, Shim S C (2004). Chem Commun.

[25] Lebedev A Y, Khartulyari A S, Voskoboynikov A Z (2005). J Org Chem.

[26] Pabba C, Wang H-J, Mulligan S R, Chen Z-J, Stark T M, Gregg B T (2005). Tetrahedron Lett.

[27] Liu R, Zhu Y, Qin L, Ji S (2008). Synth Commun.

[28] Gao M, Liu X, Wang X, Cai Q, Ding K (2011). Chin J Chem.

[29] Lee H K, Cho C S (2013). Synth Commun.

[30] Veerareddy A, Gogireddy S, Dubey P K (2014). J Heterocycl Chem.

[31] Kylmälä T, Udd S, Tois J, Franzén R (2010). Tetrahedron Lett.

