# Supplementary material for: Synthesis of N-phenyl- and N-thiazolyl-1H-indazoles by copper-catalyzed intramolecular N-arylation of ortho-chlorinated arylhydrazones
Source: Beilstein J Org Chem. 2022 Aug 23;18:1079–87. doi: 10.3762/bjoc.18.110 (PMC9443352; doi:10.3762/bjoc.18.110)
Supplement: File 1 — Reaction analysis by 1H and 13C NMR spectroscopy, characterization data, NMR spectra for the 1H-indazoles and HRMS spectra for the arylhydrazones and 1H-indazoles. [file Beilstein_J_Org_Chem-18-1079-s001.pdf]

BEILSTEIN JOURNAL OF ORGANIC CHEMISTRY

Supporting Information

for

Synthesis of *N*-phenyl- and *N*-thiazolyl-1*H*-indazoles by copper-catalyzed intramolecular *N*-arylation of *ortho*-chlorinated arylhydrazones

Yara Cristina Marchioro Barbosa, Guilherme Caneppele Paveglio,
Claudio Martin Pereira de Pereira, Sidnei Moura, Cristiane Storck Schwalm,
Gleison Antonio Casagrande and Lucas Pizzuti

Beilstein J. Org. Chem. **2022,** *18,* 1079–1087. doi:10.3762/bjoc.18.110

Reaction analysis by ^{1}H and ^{13}C NMR spectroscopy, characterization data, NMR spectra for the 1*H*-indazoles and HRMS spectra for the arylhydrazones and 1*H*-indazoles

License and Terms: This is a supporting information file under the terms of the Creative Commons Attribution License (https://creativecommons.org/licenses/by/4.0). Please note that the reuse, redistribution and reproduction in particular requires that the author(s) and source are credited and that individual graphics may be subject to special legal provisions.
The license is subject to the *Beilstein Journal of Organic Chemistry* terms and conditions: (https://www.beilstein-journals.org/bjoc/terms)

Table of Content

Analysis of the results of some reactions by ^{1}H and ^{13}C NMR S2

Characterization data of compounds **1**–**4** S10

^{1}H and ^{13}C NMR spectra of *N*-phenyl-1*H*-indazoles S17

^{1}H and ^{13}C NMR spectra of *N*-thiazolyl-1*H*-indazoles S25

HRMS spectra of *N*-phenylhydrazones **1a**–**i** S32

HRMS spectra of *N*-phenyl-1*H*-indazoles S41

HRMS spectra of *N*-thiazolylhydrazones **3a**–**i** S50

HRMS spectra of *N*-thiazolyl-1*H*-indazoles S59

References S66

Reaction analysis by ^{1}H and ^{13}C NMR

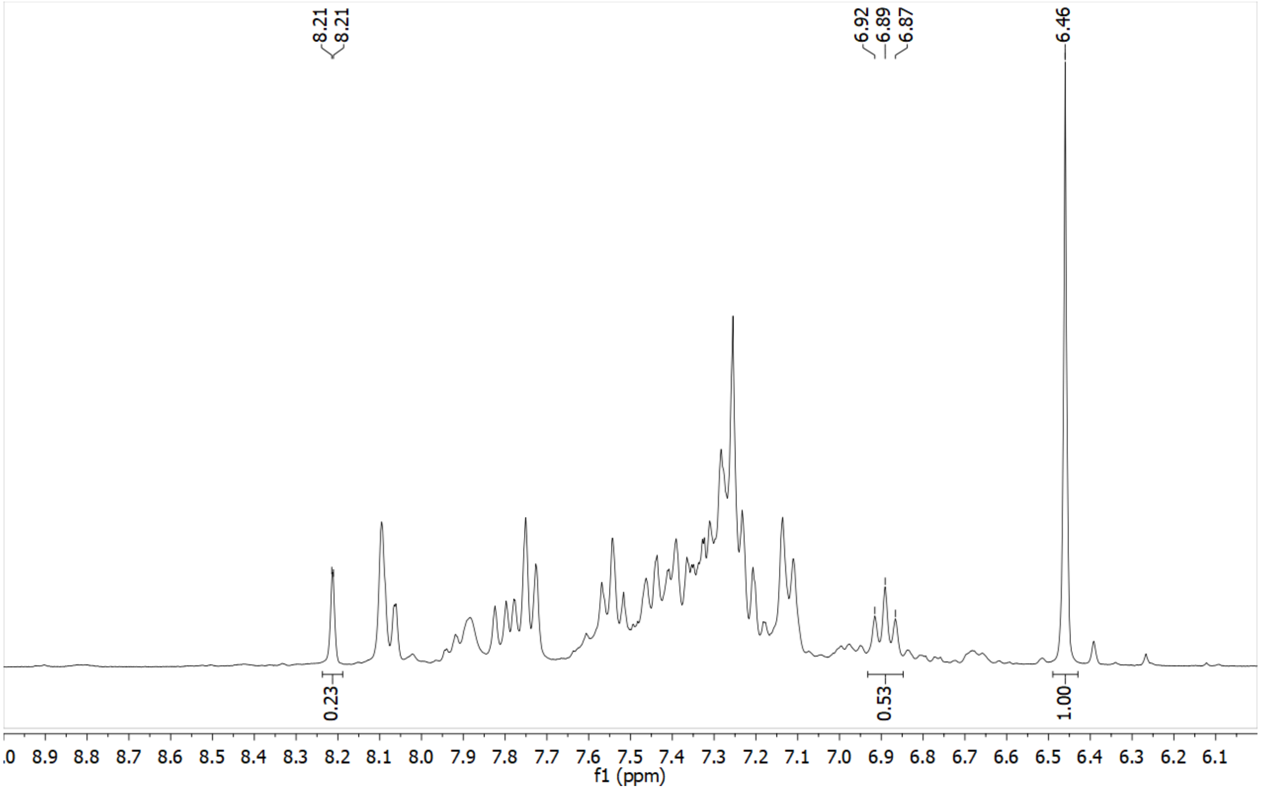

Figure S1. ^{1}H NMR spectrum of the crude product of the reaction of **1a** carried out at 100 °C + trichloroethylene (TCE, 300 MHz, $CDCl_3$).

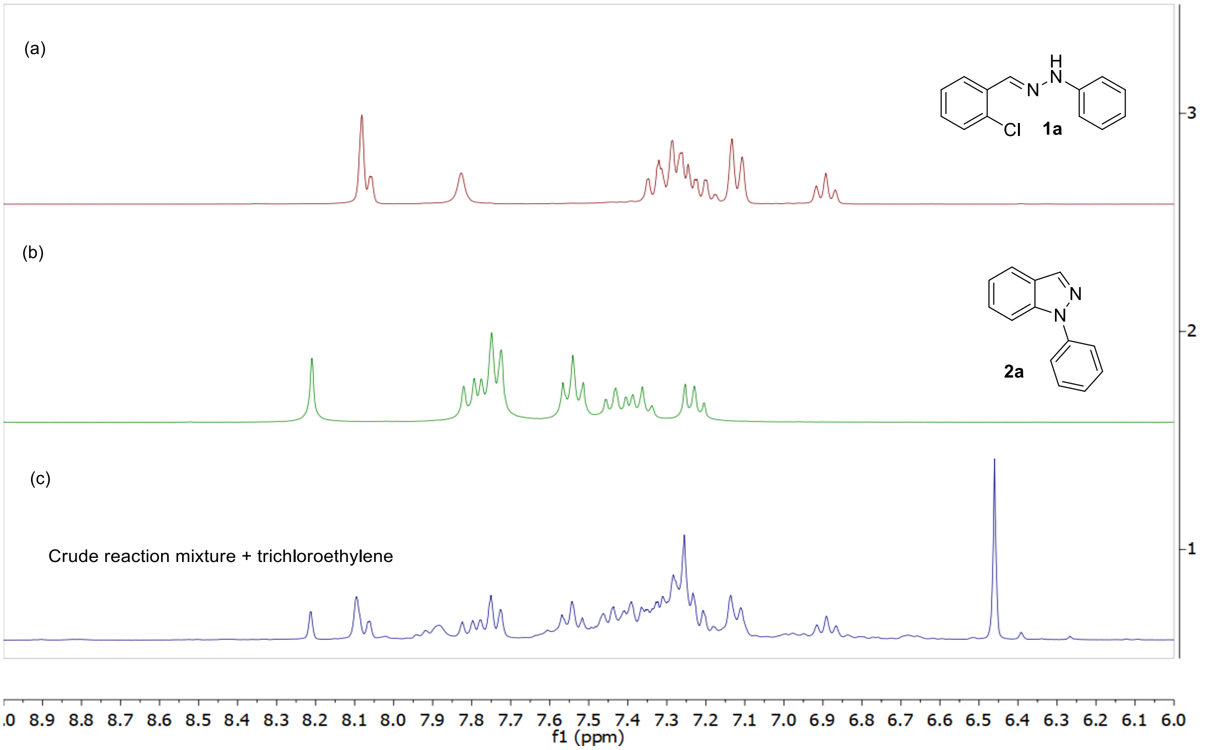

Figure S2. ^{1}H NMR spectra of (a) **1a** (300 MHz, $CDCl_3$), (b) **2a** (300 MHz, $CDCl_3$), and (c) TCE + the crude product of the reaction of **1a** carried out at 100 °C (300 MHz, $CDCl_3$).

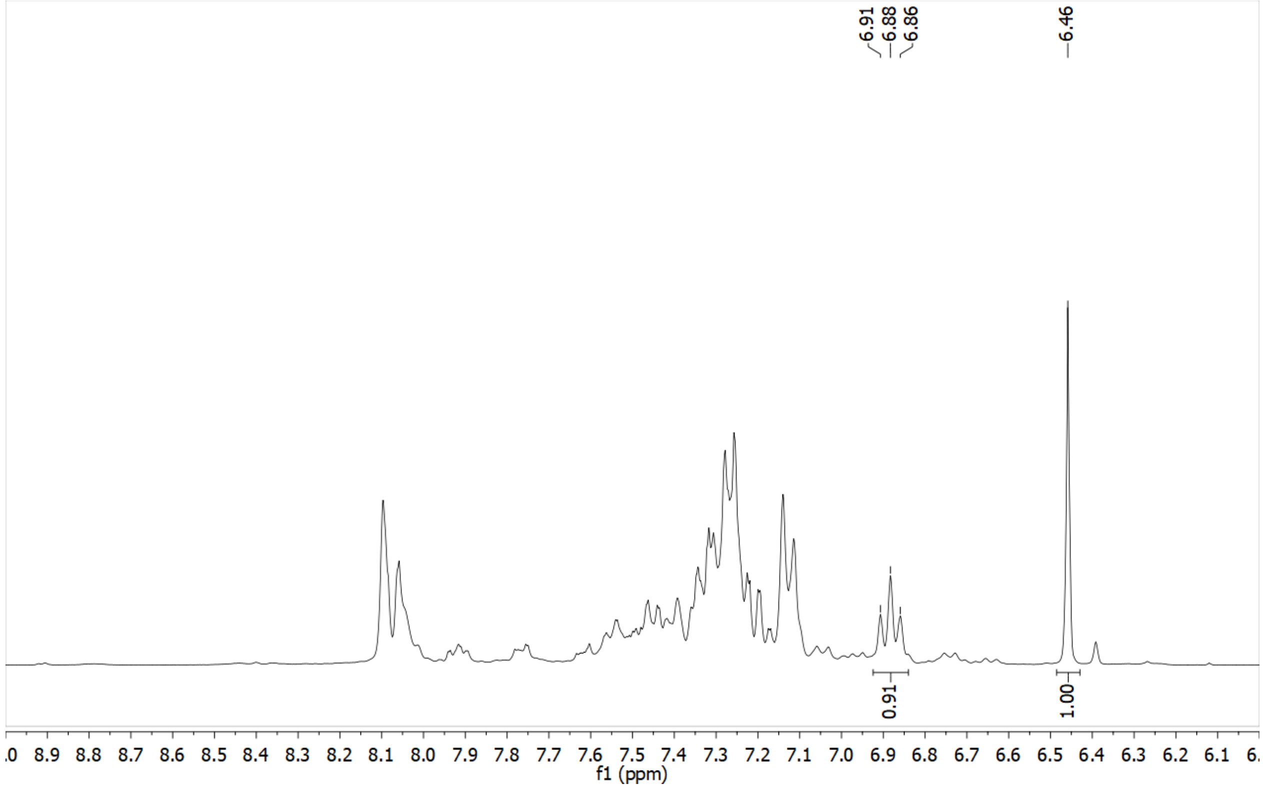

Figure S3. ^{1}H NMR spectrum of the crude product of the reaction of **1a** carried out in NMP + TCE (300 MHz, $CDCl_3$).

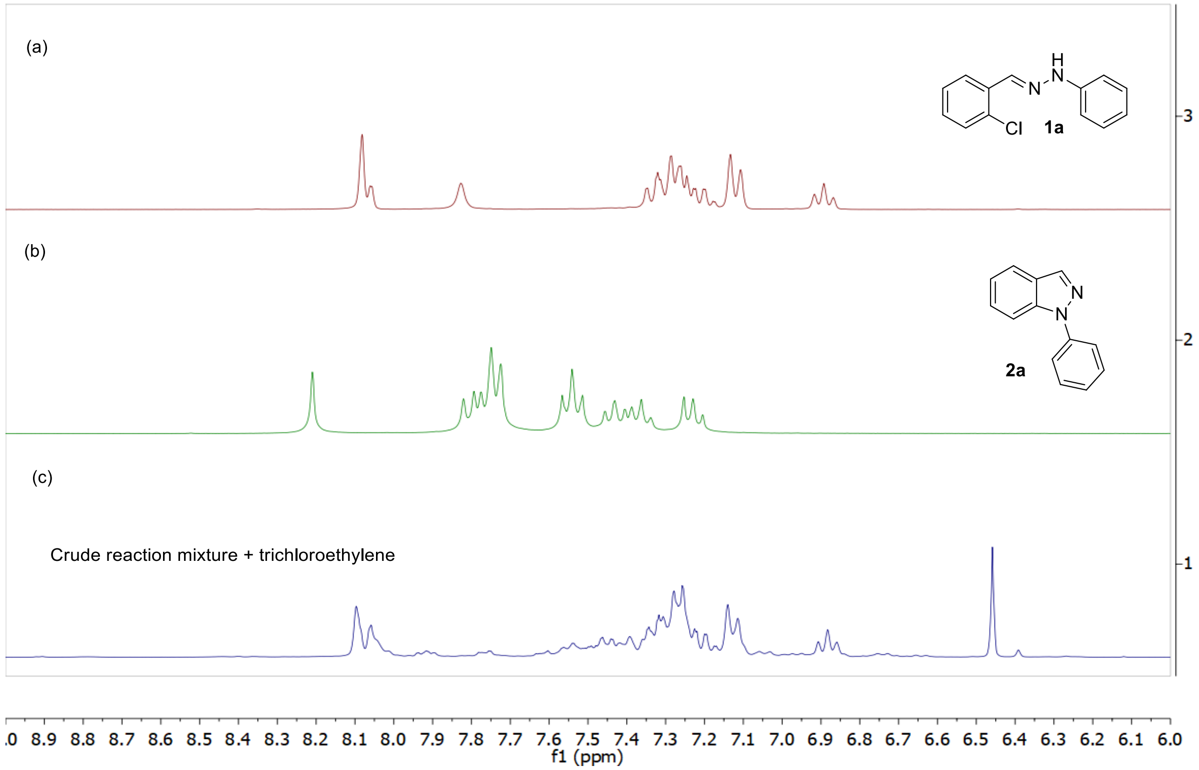

Figure S4. ^{1}H NMR spectra of (a) **1a** (300 MHz, $CDCl_3$), (b) **2a** (300 MHz, $CDCl_3$), and (c) TCE + the crude product of the reaction of **1a** carried out in NMP (300 MHz, $CDCl_3$).

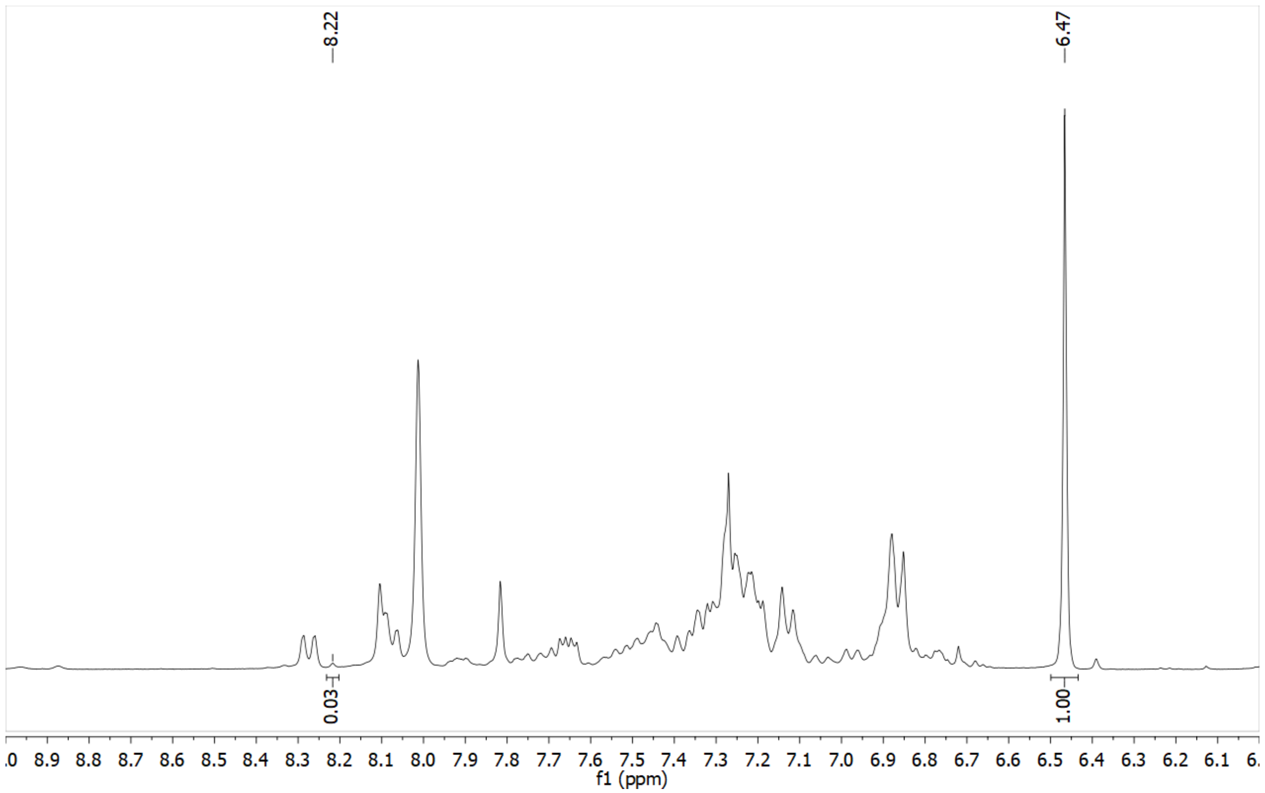

Figure S5. ^{1}H NMR spectrum of the crude product of the one pot reaction of *o*-chlorobenzaldehyde and phenylhydrazine + TCE (300 MHz, $CDCl_3$).

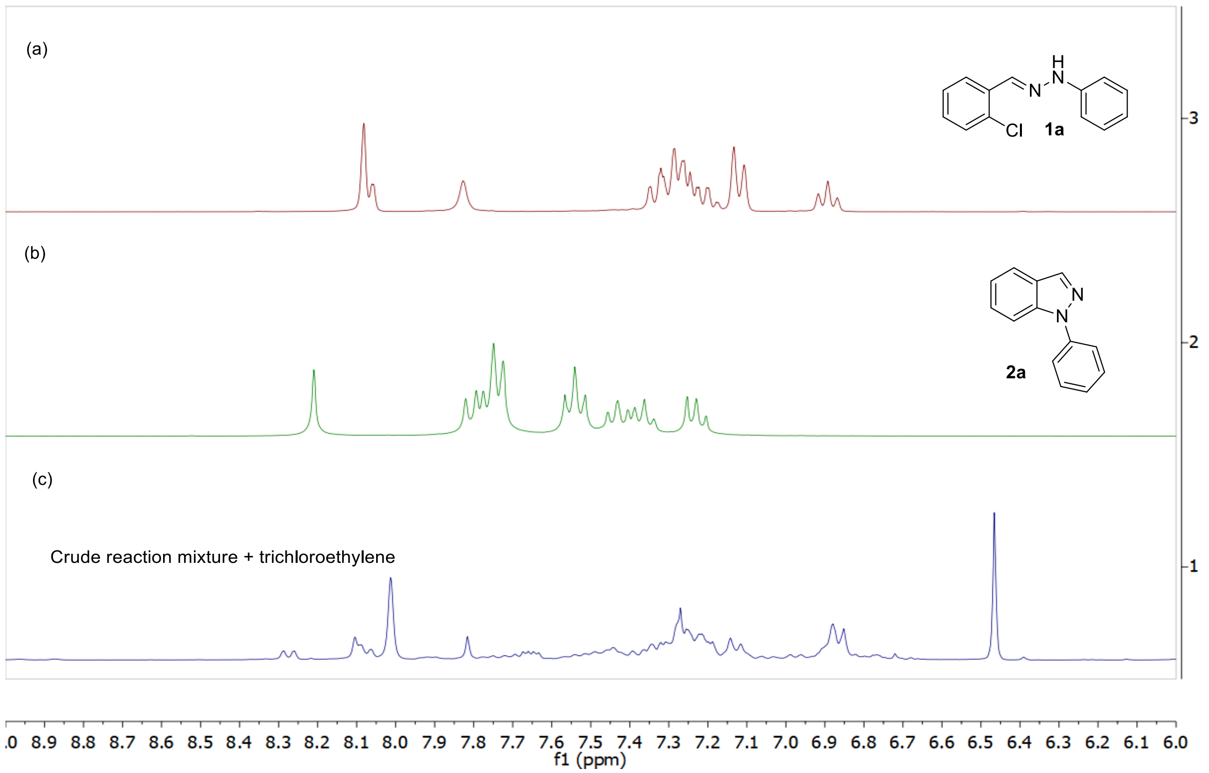

Figure S6. ^{1}H NMR spectra of (a) **1a** (300 MHz, $CDCl_3$), (b) **2a** (300 MHz, $CDCl_3$), and (c) TCE + the crude product of the one pot reaction of *o*-chlorobenzaldehyde and phenylhydrazine (300 MHz, $CDCl_3$).

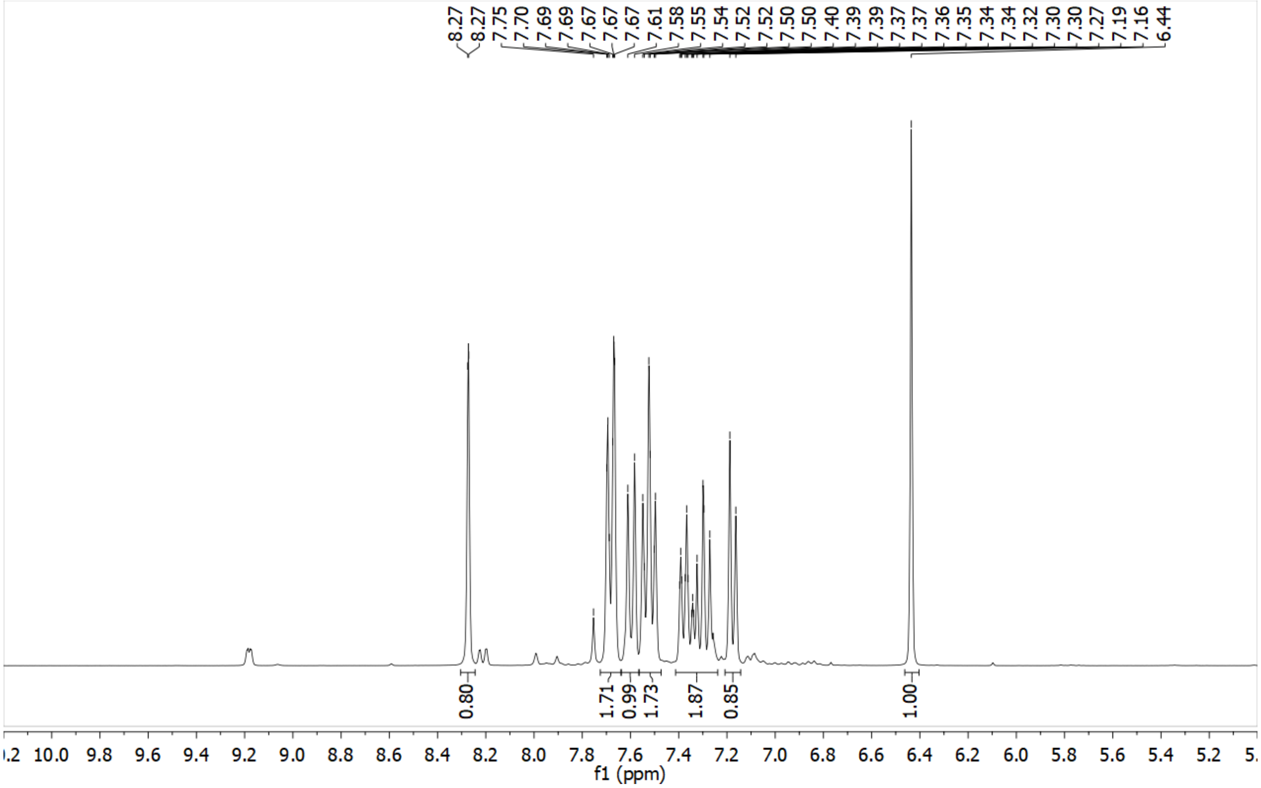

Figure S7. ^{1}H NMR spectrum of the crude product of the reaction of **1i** without CuI + TCE (300 MHz, $CDCl_3$).

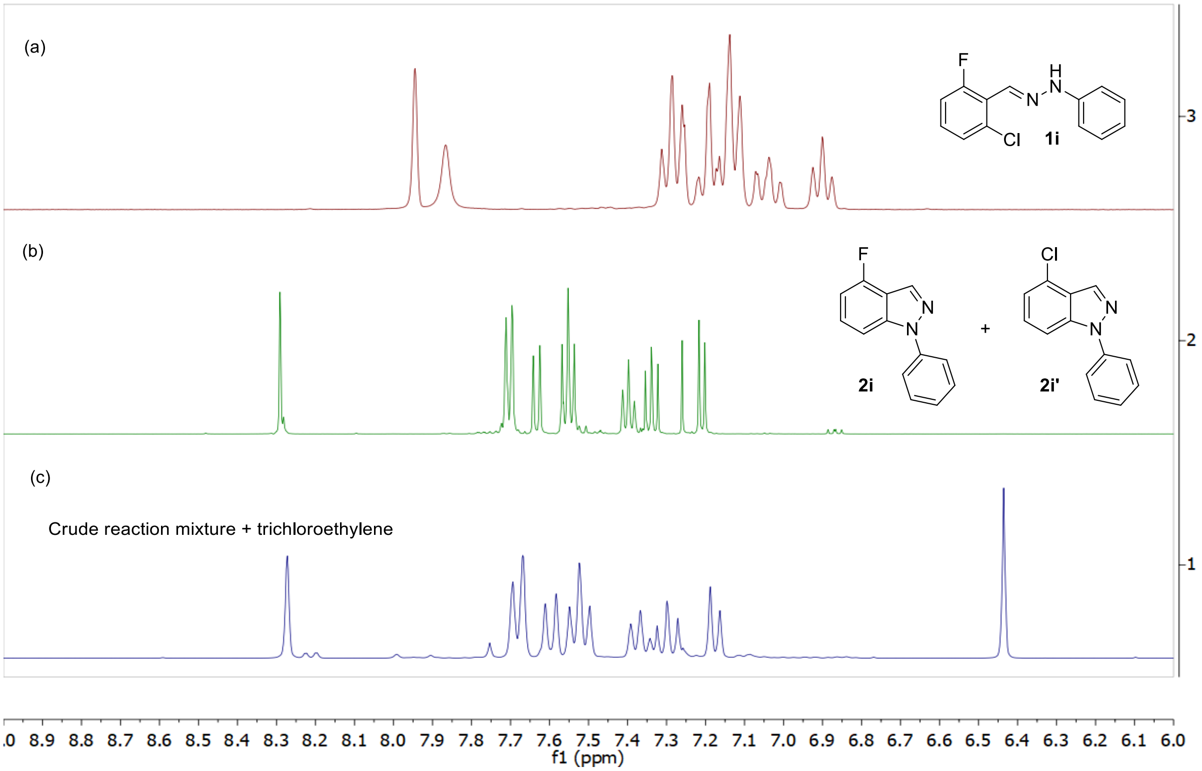

Figure S8. ^{1}H NMR spectra of (a) **1i** (300 MHz, $CDCl_3$), (b) mixture of **2i** and **2i’** (500 MHz, $CDCl_3$), and (c) TCE + the crude product of the reaction of **1i** without CuI (300 MHz, $CDCl_3$).

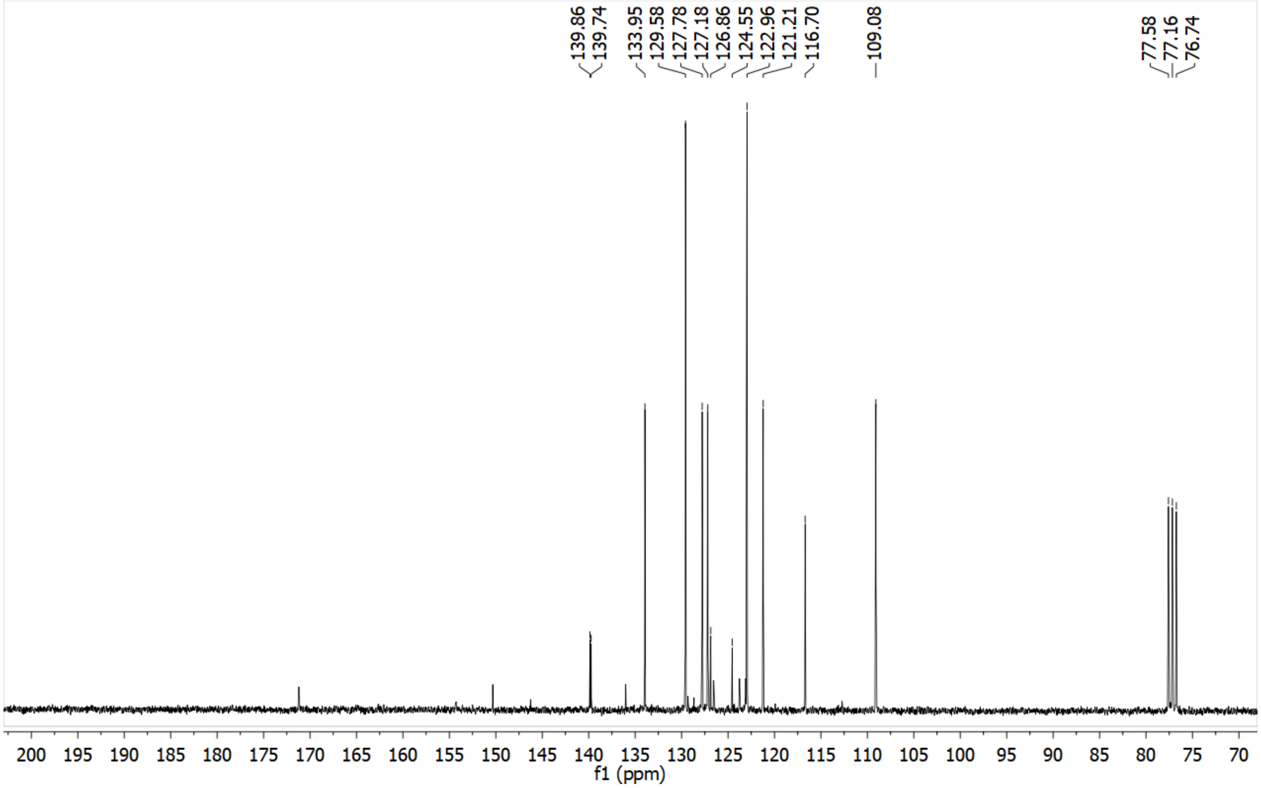

Figure S9. ^{13}C NMR spectrum of the crude product of the reaction of **1i** without CuI + TCE (75 MHz, $CDCl_3$).

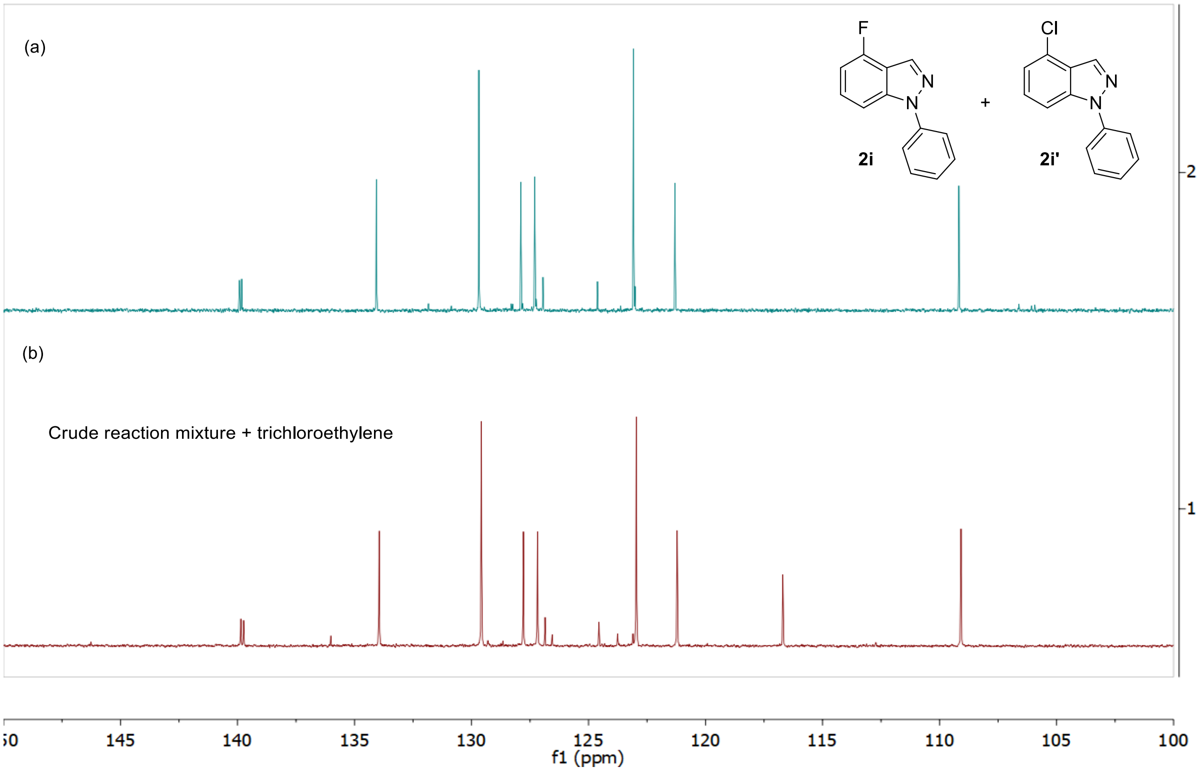

Figure S10. ^{13}C NMR spectra of (a) mixture of **2i** and **2i'** (100 MHz, $CDCl_3$) and (b) TCE + the crude product of the reaction of **1i** without CuI (75 MHz, $CDCl_3$).

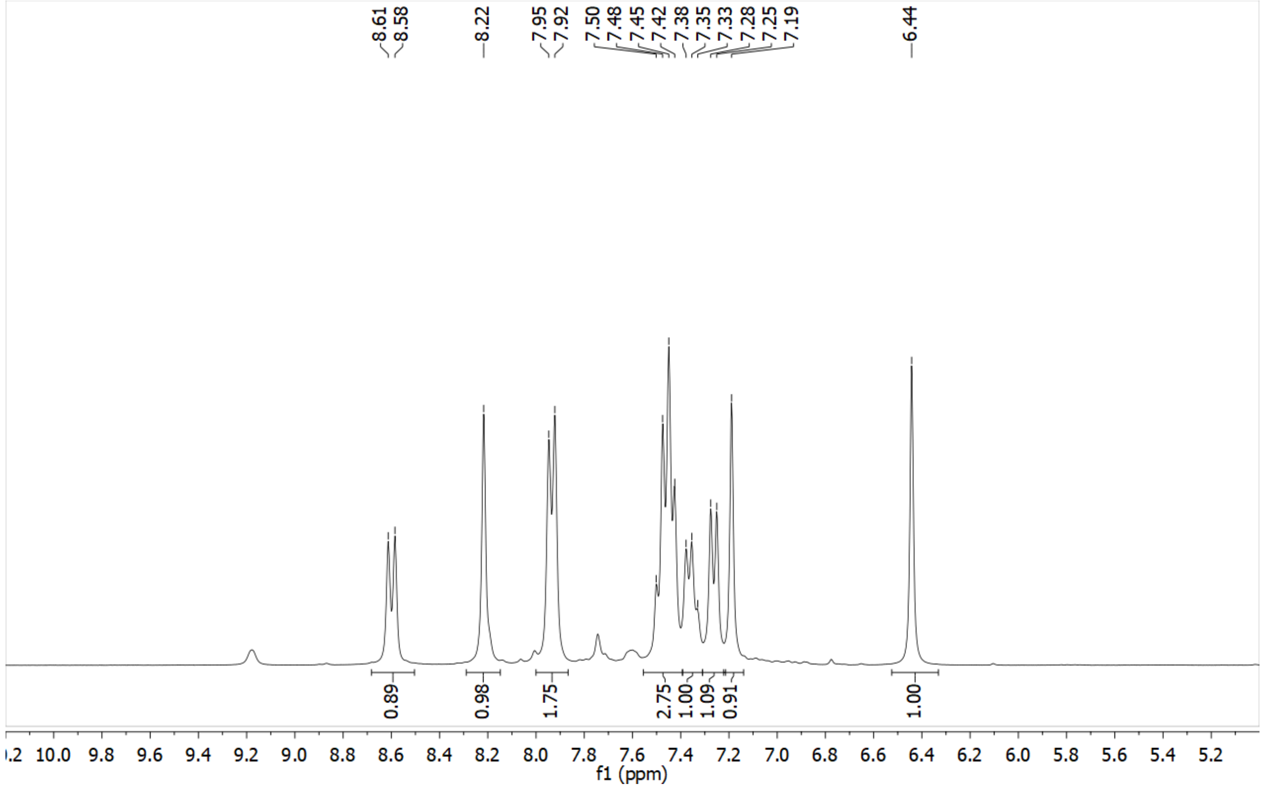

Figure S11. ^{1}H NMR spectrum of the crude product of the reaction of **3i** without CuI + TCE (300 MHz, $CDCl_3$).

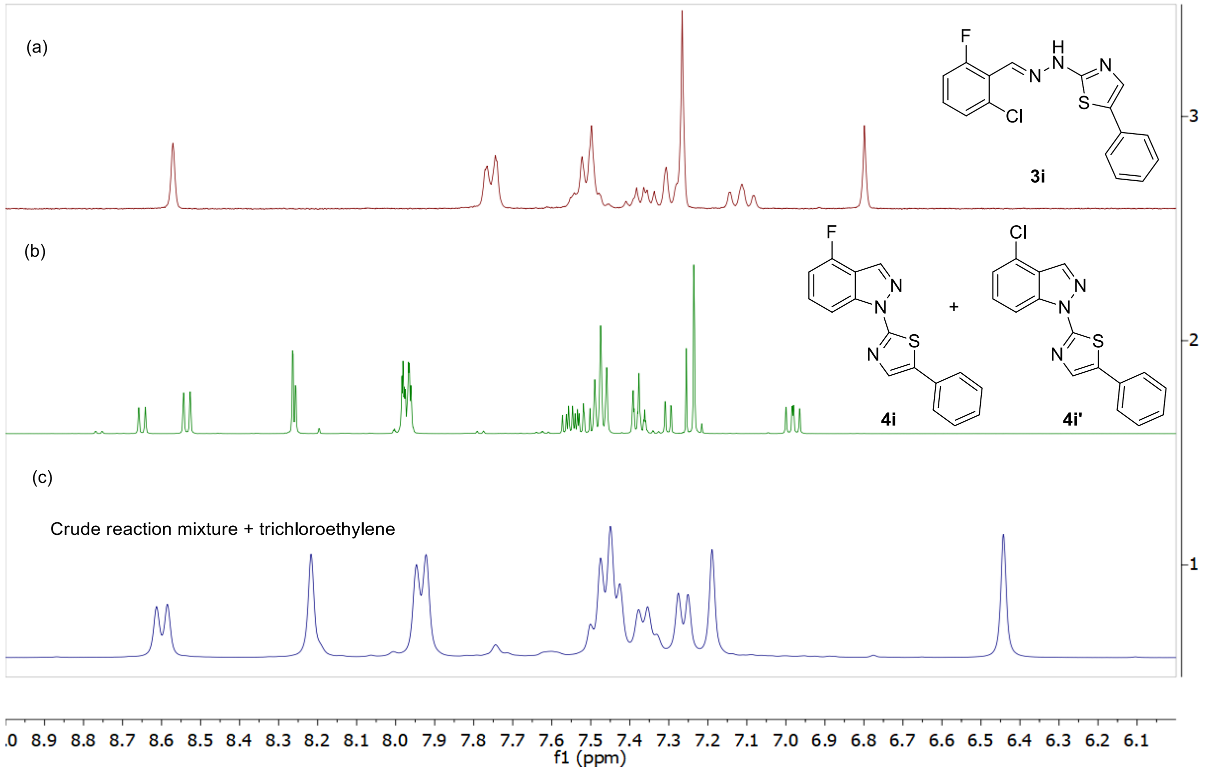

Figure S12. ^{1}H NMR spectra of (a) **3i** (300 MHz, $CDCl_3$), (b) mixture of **4i** and **4i'** (500 MHz, $CDCl_3$), and (c) TCE + the crude product of the reaction of **3i** without CuI (300 MHz, $CDCl_3$).

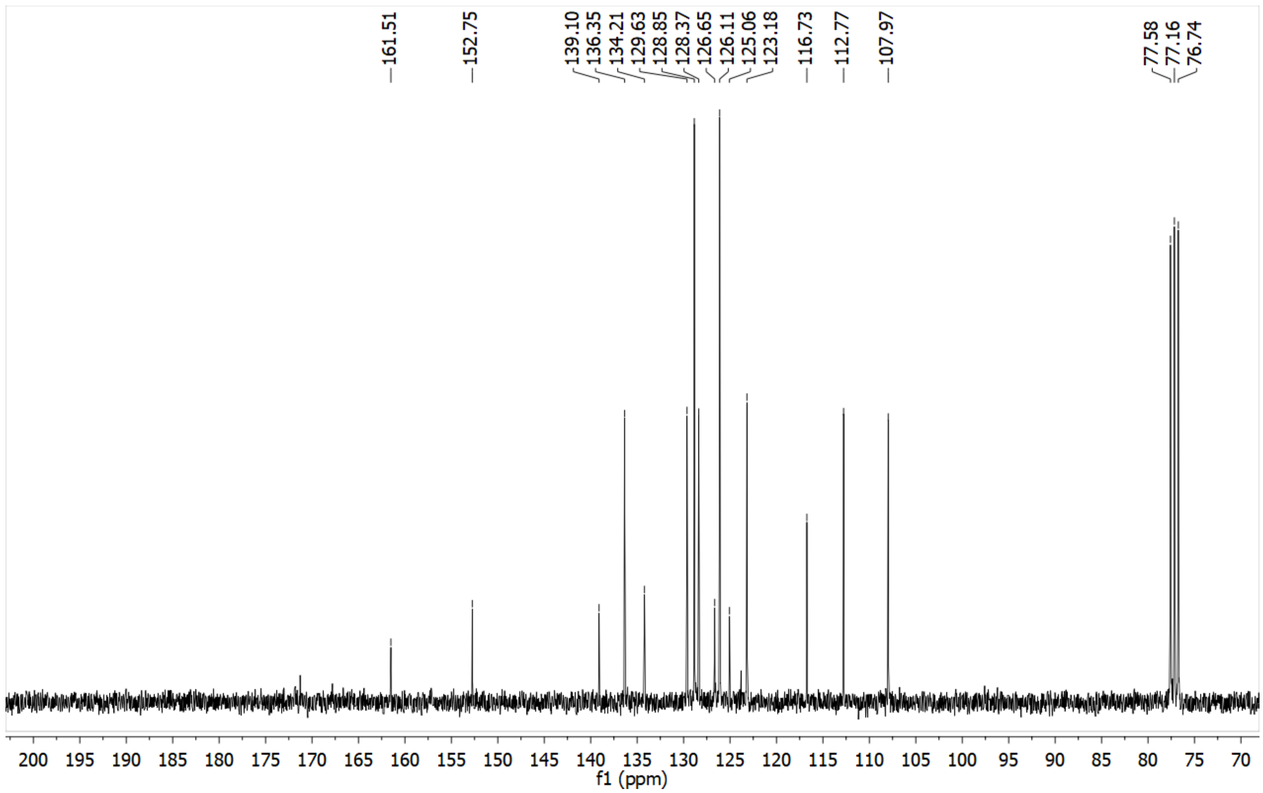

Figure S13. ^{13}C NMR spectrum of the crude product of the reaction of **3i** without CuI + TCE (75 MHz, $CDCl_3$).

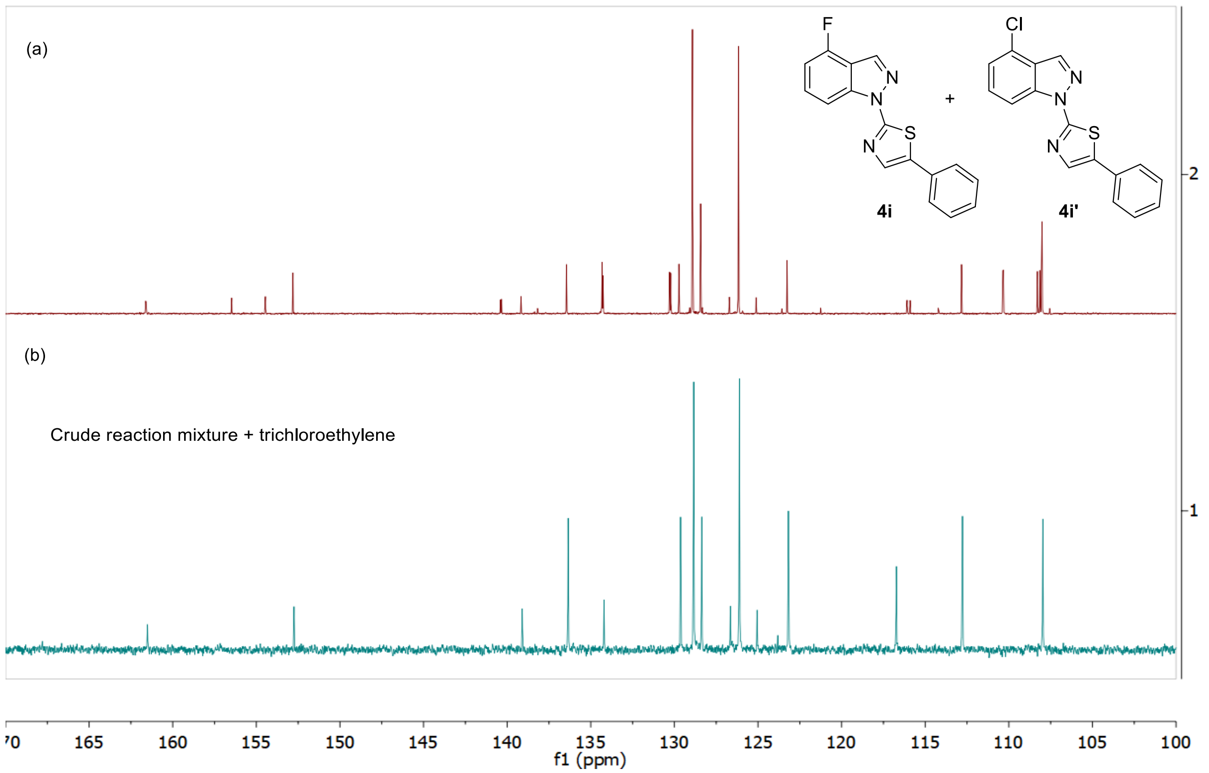

Figure S14. ^{13}C NMR spectra of (a) mixture of **4i** and **4i'** (75 MHz, $CDCl_3$) and (b) TCE + the crude product of the reaction of **3i** without CuI (100 MHz, $CDCl_3$).

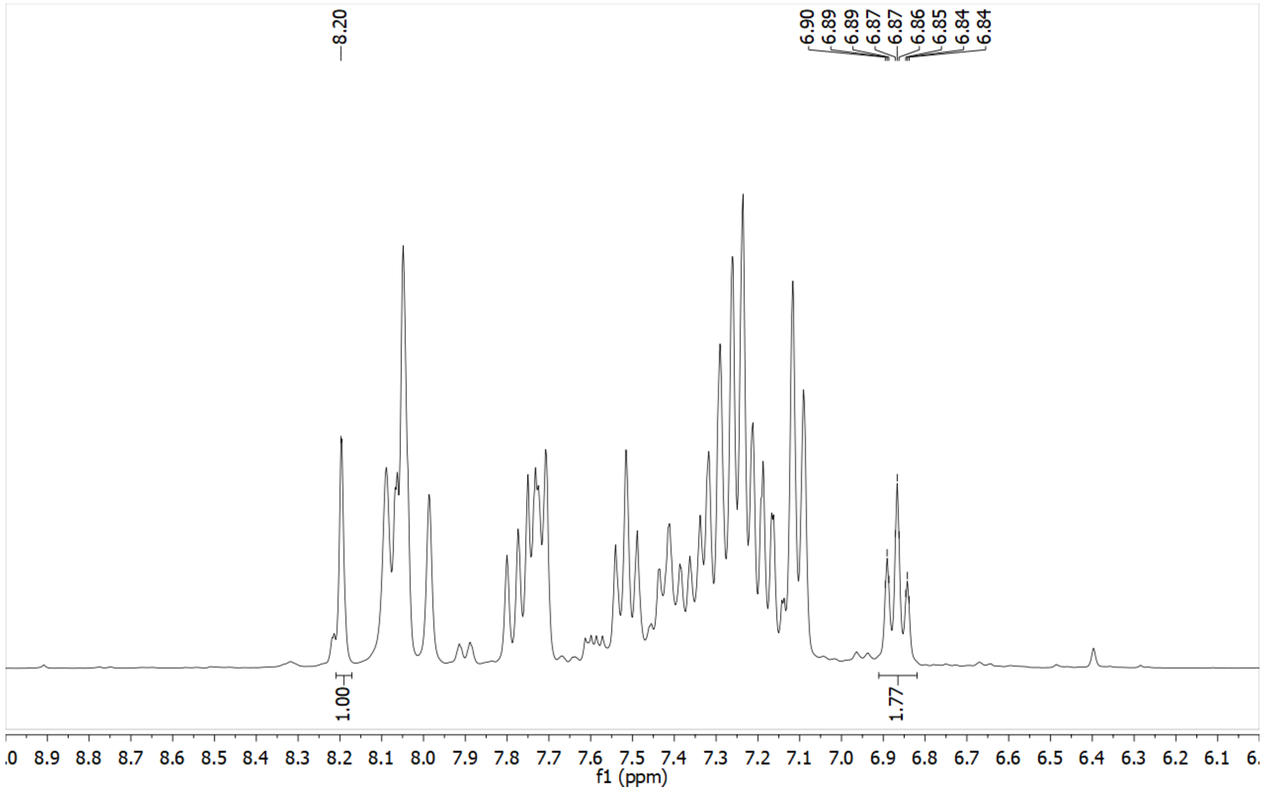

Figure S15. ^{1}H NMR spectrum of the crude product of the reaction of **1a** carried out in gram scale (300 MHz, $CDCl_3$).

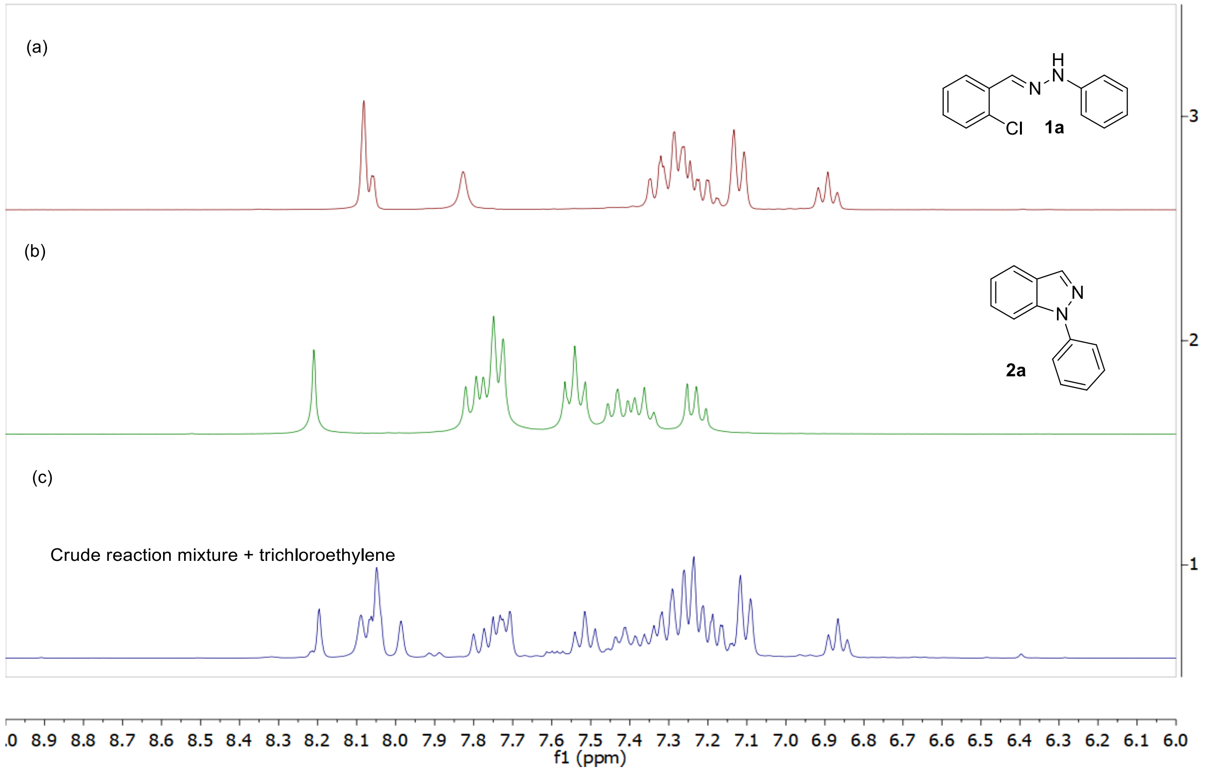

Figure S16. ^{1}H NMR spectra of (a) **1a** (300 MHz, $CDCl_3$), (b) **2a** (300 MHz, $CDCl_3$), and (c) the crude product of the reaction of **1a** carried out in gram scale (300 MHz, $CDCl_3$).

Characterization data of compounds 1–4

Characterization data of hydrazones 1a–i

(*Z*,*E*)-1-(2-Chlorobenzylidene)-2-phenylhydrazine (**1a**) [CAS number 34158-76-4]

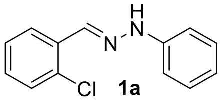

1a

Yellowish solid, yield 1.016 g (88%), mp 77−79 °C (lit. 84.7−85.5 °C [1]). HRMS (ESI-TOF) for $C_{13}H_{12}ClN_2$ $[M+H]^+$ calcd. 231.0684, found 231.0669.

(*Z*,*E*)-1-(2-Chloro-4-methylbenzylidene)-2-phenylhydrazine (**1b**)

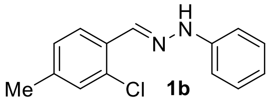

1b

Yellowish solid, yield 1.043 g (85%), mp 90−93 °C. HRMS (ESI-TOF) for $C_{14}H_{14}ClN_2$ $[M+H]^+$ calcd. 245.0840, found 245.0836.

(*Z*,*E*)-1-(2-Chloro-4-methoxybenzylidene)-2-phenylhydrazine (**1c**)

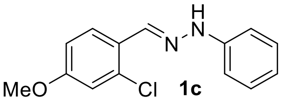

1c

Yellowish solid, yield 1.203 g (92%), mp 100−102 °C. HRMS (ESI-TOF) for $C_{14}H_{14}ClN_2O$ $[M+H]^+$ calcd. 261.0789, found 261.0798.

(*Z*,*E*)-1-(2-Chloro-4-fluorobenzylidene)-2-phenylhydrazine (**1d**)

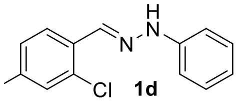

1d

Yellowish solid, yield 1.130 g (90%), mp 99−101 °C. HRMS (ESI-TOF) for $C_{13}H_{11}ClFN_2$ $[M+H]^+$ calcd. 249.0589, found 249.0588.

(*Z*,*E*)-1-(2,4-Dichlorobenzylidene)-2-phenylhydrazine (**1e**) [CAS number 21719-63-1]

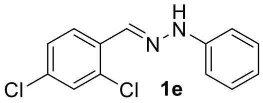

1e

Yellowish solid, yield 1.277 g (96%), mp 150−153 °C. HRMS (ESI-TOF) for $C_{13}H_{11}Cl_2N_2$ $[M+H]^+$ calcd. 265.0294, found 265.0284.

(*Z*,*E*)-1-(2-Chloro-5-fluorobenzylidene)-2-phenylhydrazine (**1f**)

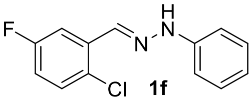

1f

Yellowish solid, yield 1.214 g (97%), mp 80−85 °C. HRMS (ESI-TOF) for $C_{13}H_{11}ClFN_2$ $[M+H]^+$ calcd. 249.0589, found 249.0594.

(*Z*,*E*)-1-(2-Chloro-5-nitrobenzylidene)-2-phenylhydrazine (**1g**) [CAS number 59670-70-1]

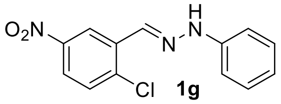

1g

Yellowish solid, yield 0.914 g (66%), mp 173−176 °C (lit. 105−108 °C [2]). HRMS (ESI-TOF) for $C_{13}H_{11}ClN_3O_2$ $[M+H]^+$ calcd. 276.0534, found 276.0530.

(*Z*,*E*)-2-Chloro-3-((2-phenylhydrazono)methyl)pyridine (**1h**)

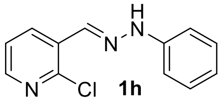

1h

Yellowish solid, yield 1.025 g (88%), mp 200−203 °C. HRMS (ESI-TOF) for $C_{12}H_{11}ClN_3$ $[M+H]^+$ calcd. 232.0636, found 232.0644.

(*Z*,*E*)- 1-(2-Chloro-6-fluorobenzylidene)-2-phenylhydrazine (**1i**) [CAS number 674348-42-6]

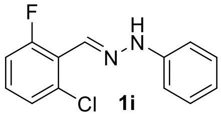

1i

Yellowish solid, yield 1.121 g (90%), mp 78−82 °C. HRMS (ESI-TOF) for $C_{13}H_{11}ClFN_2$ $[M+H]^+$ calcd. 249.0589, found 249.0594.

Characterization data of indazoles 2a,b,d–i,i'

1-Phenyl-1*H*-indazole (**2a**) [CAS number 7788-69-4]

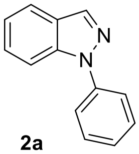

2a

Yellowish solid, yield 0.058 g (60%), mp 92–94 °C (lit. 78–80 °C [3]). ^{1}H NMR (300 MHz, $CDCl_3$) δ 8.21 (s, 1H), 7.82–7.72 (m, 4H), 7.57–7.51 (m, 2H), 7.46–7.34 (m, 2H), 7.25–7.20 (m, 1H); ^{13}C NMR (75 MHz, $CDCl_3$) δ 140.3, 138.9, 135.5, 129.6, 127.3, 126.8, 125.4, 122.9, 121.6, 121.5, 110.6; HRMS (ESI-TOF) for $C_{13}H_{11}N_2$ $[M+H]^+$ calcd. 195.0917, found 195.0922.

6-Methyl-1-phenyl-1*H*-indazole (**2b**) [CAS number 838820-88-5]

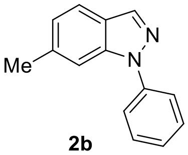

2b

Brownish solid; yield 0.010 g (10%), mp 91–93 °C (lit. 86–87 °C [3]). ^{1}H NMR (300 MHz, $CDCl_3$) δ 8.14 (s, 1H), 7.74–7.66 (m, 3H), 7.57–7.66 (m, 3H), 7.39–7.33 (m, 1H), 7.08–7.05 (m, 1H), 2.51 (s, 3H); ^{13}C NMR (75 MHz, $CDCl_3$) δ 140.4, 139.5, 137.7, 135.4, 129.6, 126.7, 123.8, 123.6, 122.9, 121.0, 110.0, 22.3. HRMS (ESI-TOF) for $C_{14}H_{13}N_2$ $[M+H]^+$ calcd. 209.1073, found 209.1059.

4.3.3. 6-Fluoro-1-phenyl-1*H*-indazole (**2d**) [CAS number 1521657-52-2]

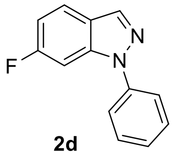

2d

Brownish solid; yield 0.025 g (23%), mp 75−77 °C; ^{1}H NMR (300 MHz, $CDCl_3$) δ 8.17 (d, J = 1.0 Hz, 1H), 7.74 (dd, J = 8.8, 5.2 Hz, 1H), 7.70–7.67 (m, 2H), 7.58–7.52 (m, 2H), 7.41–7.35 (m, 2H), 7.01 (td, J = 8.9, 2.2 Hz, 1H); ^{13}C NMR (75 MHz, $CDCl_3$) δ 162.8 (d, J = 245.5 Hz), 139.9, 139.2 (d, J = 12.5 Hz), 135.6, 129.7, 127.1, 122.8 (d, J = 11.0 Hz), 122.8, 122.2, 111.5 (d, J = 26.0 Hz), 96.6 (d, J = 27.4 Hz). HRMS (ESI-TOF) for $C_{13}H_{10}FN_2$ $[M+H]^+$ calcd. 213.0823, found 213.0809.

6-Chloro-1-phenyl-1*H*-indazole (**2e**) [CAS number 1582296-38-5]

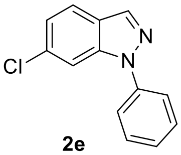

2e

Yellowish solid, yield 0.045 g (39%), mp 89−91 °C; ^{1}H NMR (300 MHz, DMSO-d_6) δ 8.40 (d, J = 1.1 Hz, 1H), 7.90 (d, J = 8.6 Hz, 1H), 7.84–7.83 (m, 1H), 7.77–7.74 (m, 2H), 7.61–7.56 (m, 2H), 7.44–7.38 (m, 1H), 7.27 (dd, J = 8.6, 1.7 Hz, 1H); ^{13}C NMR (75 MHz, DMSO-d_6) δ 139.2, 138.4, 135.9, 132.6, 129.7, 127.0, 123.8, 123.1, 122.3, 110.0; HRMS (ESI-TOF) for $C_{13}H_{10}ClN_2$ $[M+H]^+$ calcd. 229.0527, found 229.0526.

5-Fluoro-1-phenyl-1*H*-indazole (**2f**) [CAS number 350044-22-3]

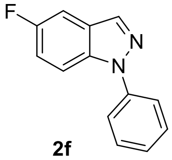

2f

Brownish solid, yield 0.056 g (53%), mp 56−58 °C; ^{1}H NMR (500 MHz, $CDCl_3$) δ 8.17 (d, J = 1.0 Hz, 1H), 7.72–7.68 (m, 3H), 7.57–7.53 (m, 2H), 7.43–7.37 (m, 2H), 7.21 (td, J = 9.0, 2.4 Hz, 1H); ^{13}C NMR (125 MHz, $CDCl_3$) δ 158.3 (d, J = 239.4 Hz), 140.0, 135.9, 135.1 (d, J = 5.5 Hz), 129.7, 127.1, 125.5 (d, J = 10.3 Hz), 122.8, 116.7 (d, J = 27.4 Hz), 111.7 (d, J = 9.5 Hz), 105.4 (d, J = 23.5 Hz). HRMS (ESI-TOF) for $C_{13}H_{10}FN_2$ $[M+H]^+$ calcd. 213.0823, found 213.0830.

5-Nitro-1-phenyl-1*H*-indazole (**2g**) [CAS number 838821-02-6]

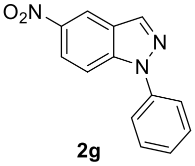

2g

Yellowish solid, yield 0.019 g (16%), mp 184−186 °C (lit. 178−180 °C [2]); ^{1}H NMR (500 MHz, $CDCl_3$) δ 8.81 (dd, J = 2.1, 0.7 Hz, 1H), 8.42 (d, J = 0.9 Hz, 1H), 8.32 (dd, J = 9.3, 2.1 Hz, 1H), 7.79 (dt, J = 9.2, 0.8 Hz, 1H), 7.73–7.70 (m, 2H), 7.62–7.59 (m, 2H), 7.49–7.45 (m, 1H); ^{13}C NMR (125 MHz, $CDCl_3$) δ 143.0, 140.7, 139.1, 137.7, 129.9, 128.1, 124.6, 123.3, 122.4, 119.2, 110.9; HRMS (ESI-TOF) for $C_{13}H_{10}N_3O_2$ $[M+H]^+$ calcd. 240.0768, found 240.0760.

1-Phenyl-1*H*-pyrazolo[3,4-*b*]pyridine (**2h**) [CAS number 20208-81-5]

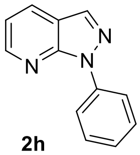

2h

Redwish solid, yield 0.068 g (70%), mp 68−70 °C (lit. 53−55 °C [3]); ^{1}H NMR (500 MHz, $CDCl_3$) δ 8.64 (dd, *J* = 4.5, 1.6 Hz, 1H), 8.27–8.25 (m, 2H), 8.21 (s, 1H), 8.13 (dd, *J* = 8.0, 1.7 Hz, 1H), 7.56–7.52 (m, 2H), 7.35–7.31 (m, 1H), 7.22 (dd, *J* = 8.0, 4.5 Hz, 1H); ^{13}C NMR (125 MHz, $CDCl_3$) δ 150.2, 149.3, 139.5, 134.0, 130.4, 129.3, 126.3, 121.5, 117.8, 117.3; HRMS (ESI-TOF) for $C_{12}H_{10}N_3$ $[M+H]^+$ calcd. 196.0866, found 196.0869.

4-Fluoro-1-phenyl-1*H*-indazole (**2i**) + 4-chloro-1-phenyl-1*H*-indazole (**2i'**) [CAS number 861327-91-5]

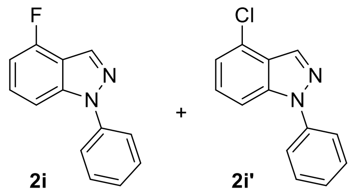

2i **2i'**

HRMS (ESI-TOF) for $C_{13}H_{10}FN_2$ $[M+H]^+$ calcd. 213.0823, found 213.0817. HRMS (ESI-TOF) for $C_{13}H_{10}ClN_2$ $[M+H]^+$ calcd. 229.0527, found 229.0525.

Characterization data of hydrazones 3a–i

(*Z*,*E*)-2-(2-(2-Chlorobenzylidene)hydrazinyl)-4-phenylthiazole (**3a**) [CAS number 324066-80-0]

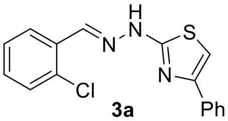

3a

Pale yellow solid, yield 0.445 mg (94%), mp 205−208 °C (lit. 220−221 °C [4]). HRMS (ESI-TOF) for $C_{16}H_{13}ClN_3S$ $[M+H]^+$ calcd. 314.0513, found 314.0504.

(*Z*,*E*)-2-(2-(2-Chloro-4-methylbenzylidene)hydrazinyl)-4-phenylthiazole (**3b**)

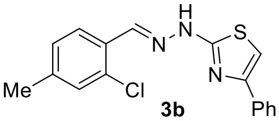

3b

Whitish solid, yield 0.356 g (72%), mp 220−223 °C; HRMS (ESI-TOF) for $C_{17}H_{15}ClN_3S$ $[M+H]^+$ calcd. 328.0670, found 328.0689.

(*Z*,*E*)-2-(2-(2-Chloro-4-methoxybenzylidene)hydrazinyl)-4-phenylthiazole (**3c**)

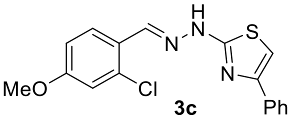

3c

Pale yellow solid, yield 0.415 g (80%), mp 190−193 °C. HRMS (ESI-TOF) for $C_{17}H_{15}ClN_3OS$ $[M+H]^+$ calcd. 344.0619, found 344.0614.

(*Z*,*E*)-2-(2-(2-Chloro-4-fluorobenzylidene)hydrazinyl)-4-phenylthiazole (**3d**)

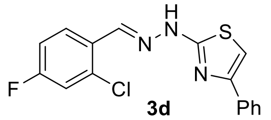

3d

Yellowish solid, yield 0.483 g (97%), mp 210−213 °C. HRMS (ESI-TOF) for $C_{16}H_{12}ClFN_3S$ $[M+H]^+$ calcd. 332.0419, found 332.0431.

(*Z*,*E*)-2-(2-(2,4-Dichlorobenzylidene)hydrazinyl)-4-phenylthiazole (**3e**) [CAS number 339284-43-4]

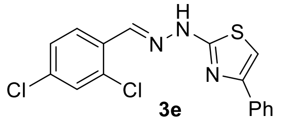

3e

Pale yellow solid, yield 0.396 g (75%), mp 207−210 °C (lit. 244−246 °C [5]). HRMS (ESI-TOF) for $C_{16}H_{12}Cl_2N_3S$ $[M+H]^+$ calcd. 348.0124, found 348.0126.

(*Z*,*E*)-2-(2-(2-Chloro-5-fluorobenzylidene)hydrazinyl)-4-phenylthiazole (**3f**)

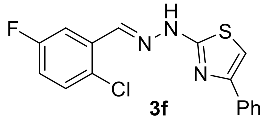

3f

Whitish solid, yield 0.451 g (90%), mp 185−188 °C. HRMS (ESI-TOF) for $C_{16}H_{12}ClFN_3S$ $[M+H]^+$ calcd. 332.0419, found 332.0411.

(*Z*,*E*)-2-(2-(2-Chloro-5-nitrobenzylidene)hydrazinyl)-4-phenylthiazole (**3g**) [CAS number 1684423-73-1]

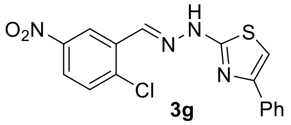

3g

Yellowish solid, yield 0.375 g (70%), mp 190−192 °C. HRMS (ESI-TOF) for $C_{16}H_{12}ClN_4O_2S$ $[M+H]^+$ calcd. 359.0364, found 359.0355.

(*Z*,*E*)-2-(2-((2-Chloropyridin-3-yl)methylene)hydrazinyl)-4-phenylthiazole (**3h**)

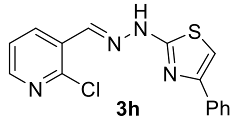

3h

Yellowish solid, yield 0.427 g (91%), mp 225−230 °C. HRMS (ESI-TOF) for $C_{15}H_{12}ClN_4S$ $[M+H]^+$ calcd. 315.0466, found 315.0472.

(*Z*,*E*)-2-(2-(2-Chloro-6-fluorobenzylidene)hydrazinyl)-4-phenylthiazole (**3i**) [CAS number 405924-76-7]

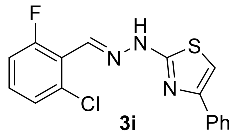

3i

Whitish solid, yield 0.484 g (97%), mp 200−203 °C. HRMS (ESI-TOF) for $C_{16}H_{12}ClFN_3S$ $[M+H]^+$ calcd. 332.0419, found 332.0419.

Characterization data of indazoles 2a,d–i,i’

2-(1*H*-Indazol-1-yl)-4-phenylthiazole (**4a**)

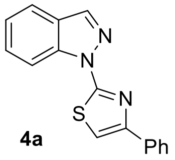

4a

Brownish solid, yield 0.049 g (35%), mp 160–163 °C; ^{1}H NMR (500 MHz, $CDCl_3$) δ 8.79–8.77 (m, 1H), 8.22 (d, J = 0.9 Hz, 1H), 8.02–8.00 (m, 2H), 7.80 (d, J = 8.0 Hz, 1H), 7.66–7.63 (m, 1H), 7.51–7.48 (m, 2H), 7.40–7,34 (m, 2H), 7.24 (s, 1H); ^{13}C NMR (125 MHz, $CDCl_3$) δ 162.0, 152.8, 138.4, 138.2, 134.5, 129.1, 128.9, 128.4, 126.2, 126.0, 123.6, 121.3, 114.2, 107.6; HRMS (ESI-TOF) for $C_{16}H_{12}N_3S$ $[M+H]^+$ calcd. 278.0746, found 278.0746.

2-(6-Fluoro-1*H*-indazol-1-yl)-4-phenylthiazole (**4d**)

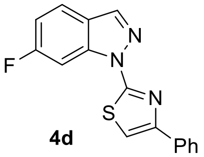

4d

Brownish solid, yield 0.018 g (12%), mp 129–132 °C; ^{1}H NMR (500 MHz, $CDCl_3$) δ 8.44 (ddd, J = 9.4, 1.8, 1.0 Hz, 1H), 8.16 (d, J = 0.9 Hz, 1H), 7.99–7.97 (m, 2H), 7.73 (dd, J = 8.8, 5.0 Hz, 1H), 7.50– 7.47 (m, 2H), 7.40–7.37 (m, 1H), 7.23 (s, 1H), 7.11 (td, J = 8.9, 2.3 Hz, 1H); ^{13}C NMR (125 MHz, $CDCl_3$) δ 163.5 (d, J = 247.5 Hz), 161.6, 152.8, 138.7 (d, J = 13.9 Hz), 138.0, 134.2, 129.0, 128.4, 126.2, 122,6, 122,6 (d, J = 11.0 Hz), 113.2 (d, J = 26.0 Hz), 107.8, 100.6 (d, J = 28.2 Hz); HRMS (ESI-TOF) for $C_{16}H_{11}FN_3S$ $[M+H]^+$ calcd. 296.0652, found 296.0640.

2-(6-Chloro-1*H*-indazol-1-yl)-4-phenylthiazole (**4e**)

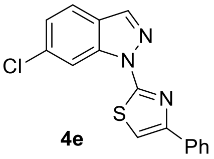

4e

Yellowish solid, yield 0.037 g (23%), mp 140–143 °C; ^{1}H NMR (500 MHz, $CDCl_3$) δ 8.74 (dd, J = 1.7, 0.9 Hz, 1H), 8.14 (d, J = 0.8 Hz, 1H), 7.97–7.95 (m, 2H), 7.67 (d, J = 8.5 Hz, 1H), 7.50–7.47 (m, 2H), 7.40–7.36 (m, 1H), 7.29 (dd, J = 8.5, 1.8 Hz, 1H), 7.21 (s, 1H); ^{13}C NMR (125 MHz, $CDCl_3$) δ 161.5, 152.8, 138.5, 137.9, 135.4, 134.2, 129.0, 128.4, 126.2, 124.6, 124.4, 122.0, 114.1, 107.9; HRMS (ESI-TOF) for $C_{16}H_{11}ClN_3S$ $[M+H]^+$ calcd. 312.0357, found 312.0362.

2-(5-Fluoro-1*H*-indazol-1-yl)-4-phenylthiazole (**4f**)

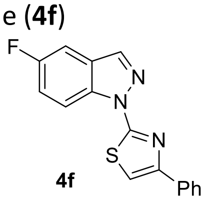

4f

Yellowish solid, yield 0.036 g (24%), mp 165–168 °C; ^{1}H NMR (500 MHz, $CDCl_3$) δ 8.74 (ddt, J = 9.0, 4.4, 0.8 Hz, 1H), 8.16 (d, J = 1.0 Hz, 1H), 7.99–7.97 (m, 2H), 7.52–7.46 (m, 2H), 7.42–7.36 (m, 3H), 7.23 (s, 1H); ^{13}C NMR (125 MHz, $CDCl_3$) δ 161.7, 159.2 (d, J = 241.7 Hz), 152.7, 137,7 (d, J = 5.2 Hz), 135.3, 134.3, 128.9, 128.4, 126.2, 126.1, 118.2 (d, J = 26.5 Hz), 115.5 (d, J = 9.1 Hz), 107.8, 105.8 (d, J = 24.0 Hz); HRMS (ESI-TOF) for $C_{16}H_{11}FN_3S$ $[M+H]^+$ calcd. 296.0652, found 296.0635.

2-(5-Nitro-1*H*-indazol-1-yl)-4-phenylthiazole (**4g**)

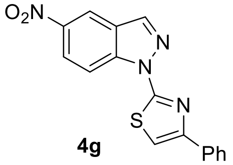

4g

Brownish solid, yield 0.031 g (19%), mp 278–280 °C; ^{1}H NMR (300 MHz, $CDCl_3$) δ 8.89 (d, *J* = 9.2 Hz, 1H), 8.77 (d, *J* = 2.1 Hz, 1H), 8.51 (dd, *J* = 9.3, 2.1 Hz, 1H), 8.39 (s, 1H), 8.00–7.97 (m, 2H), 7.53–7.48 (m, 2H), 7.44–7.38 (m, 1H), 7.32 (s, 1H); ^{13}C NMR (75 MHz, $CDCl_3$) δ 161.1, 153.2, 144.2, 140.0, 139.4, 134.0, 129.0, 128.7, 126.2, 125.3, 124.0, 118.5, 114.8, 108.8; HRMS (ESI-TOF) for $C_{16}H_{11}N_4O_2S$ $[M+H]^+$ calcd. 323.0621, found 323.0597.

4-Phenyl-2-(1*H*-pyrazolo[3,4-*b*]pyridin-1-yl)thiazole (**4h**)

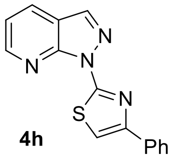

4h

Yellowish solid, yield 0.048 g (34%), mp 65–68 °C; ^{1}H NMR (500 MHz, $CDCl_3$) δ 8.77 (dd, *J* = 4.6, 1.6 Hz, 1H), 8.33 (s, 1H), 8.19 (dd, *J* = 8.0, 1.6 Hz, 1H), 8.05–8.02 (m, 2H), 7.46–7.43 (m, 2H), 7.40 (s, 1H), 7.37–7.34 (m, 1H), 7.34 (dd, *J* = 7.9, 4.6 Hz, 1H); ^{13}C NMR (125 MHz, $CDCl_3$) δ 157.5, 152.5, 150.2, 149.7, 136.8, 134.2, 130.9, 128.7, 128.3, 126.6, 119.1, 117.5, 109.4; HRMS (ESI-TOF) for $C_{15}H_{11}N_4S$ $[M+H]^+$ calcd. 279.0699, found 279.0700.

2-(4-Fluoro-1*H*-indazol-1-yl)-4-phenylthiazole (**4i**) + 2-(4-chloro-1*H*-indazol-1-yl)-4-phenylthiazole (**4i’**)

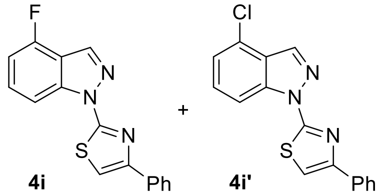

4i **4i'**

HRMS (ESI-TOF) for $C_{16}H_{11}FN_3S$ $[M+H]^+$ calcd. 296.0652, found 296.0651. HRMS (ESI-TOF) for $C_{16}H_{11}ClN_3S$ $[M+H]^+$ calcd. 312.0357, found 312.0353.

^{1}H and ^{13}C NMR spectra of *N*-phenyl-1*H*-indazoles

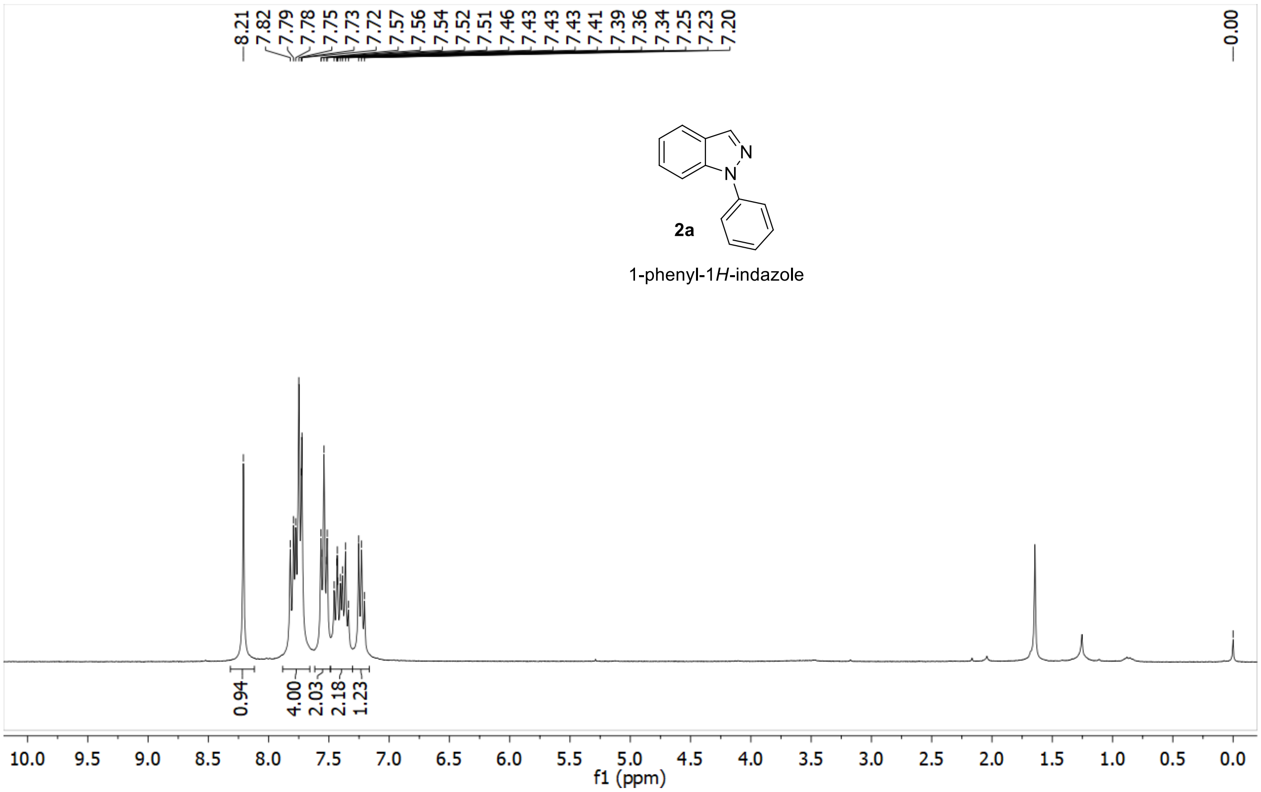

Figure S17. ^{1}H NMR spectrum of compound **2a** (300 MHz, $CDCl_3$).

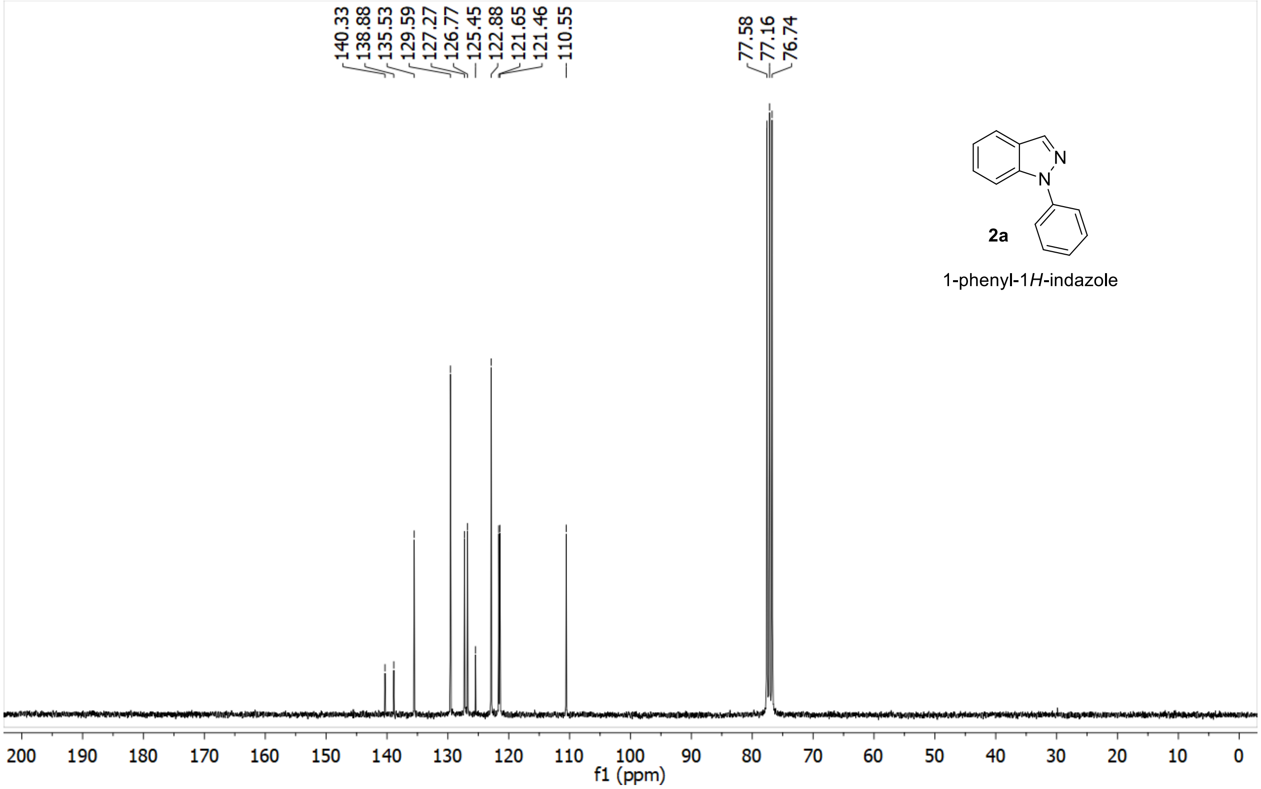

Figure S18. ^{13}C NMR spectrum of compound **2a** (75 MHz, $CDCl_3$).

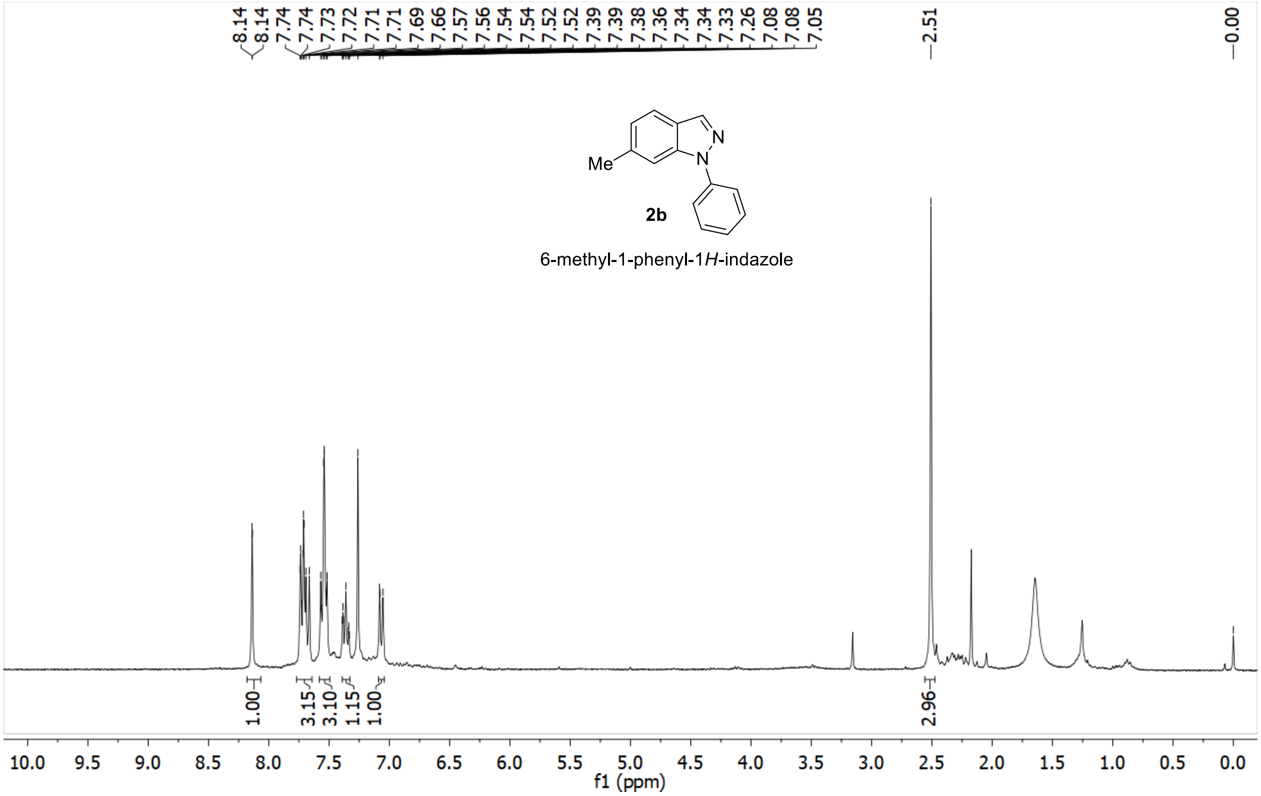

Figure S19. ^{1}H NMR spectrum of compound **2b** (300 MHz, $CDCl_3$).

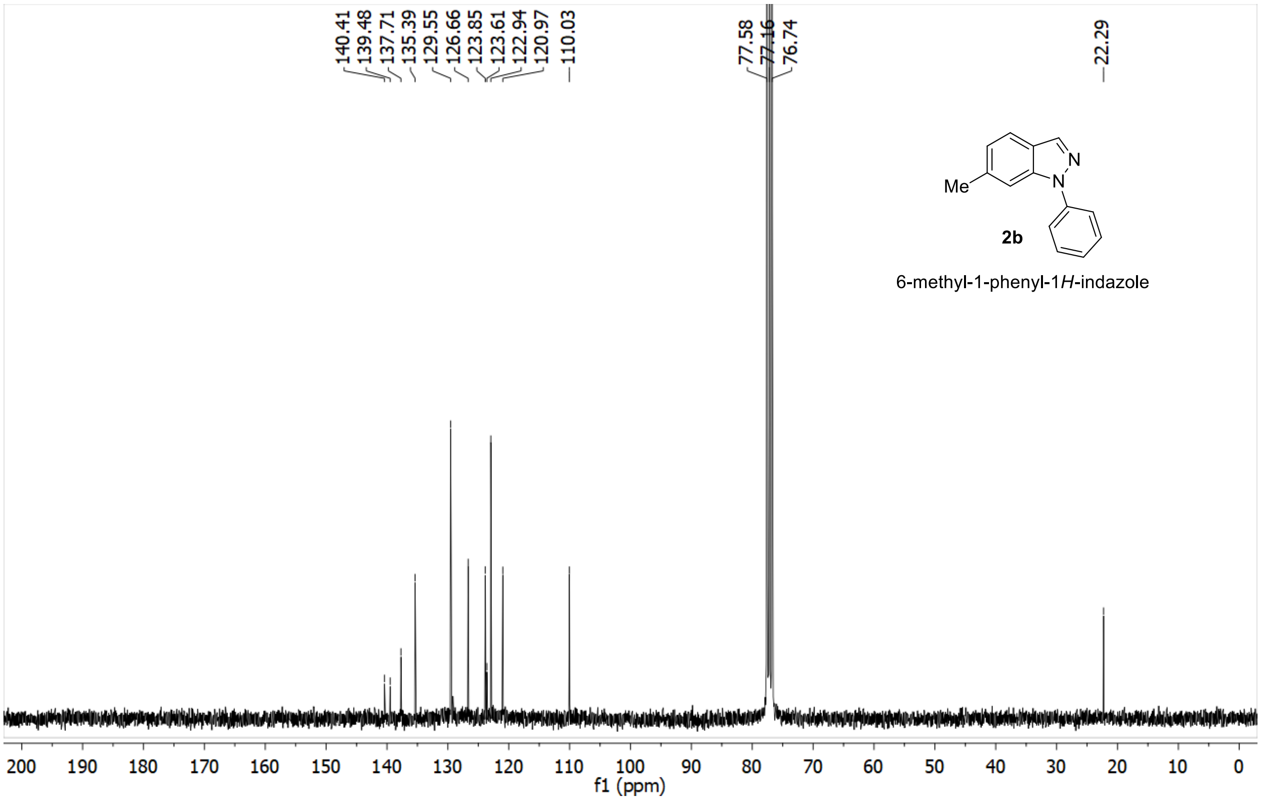

Figure S20. ^{13}C NMR spectrum of compound **2b** (75 MHz, $CDCl_3$).

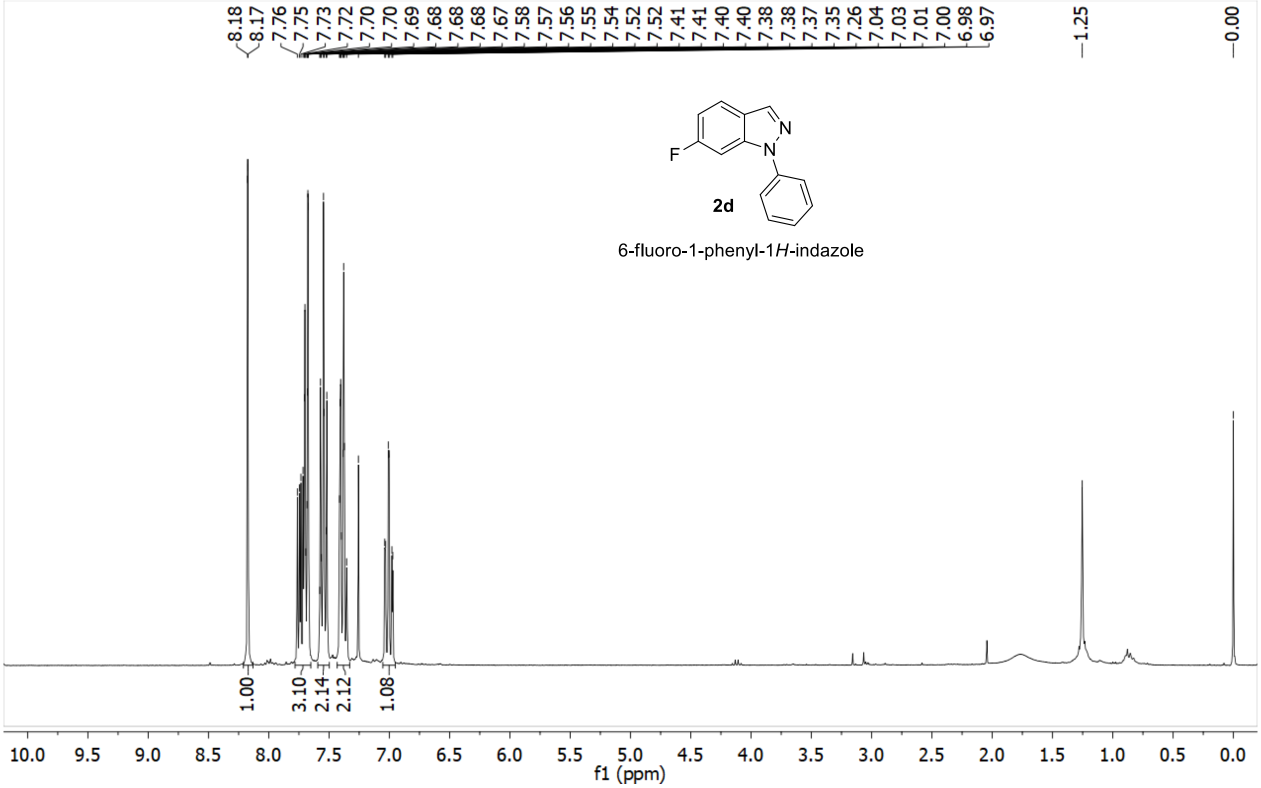

Figure S21. ^{1}H NMR spectrum of compound **2d** (300 MHz, $CDCl_3$).

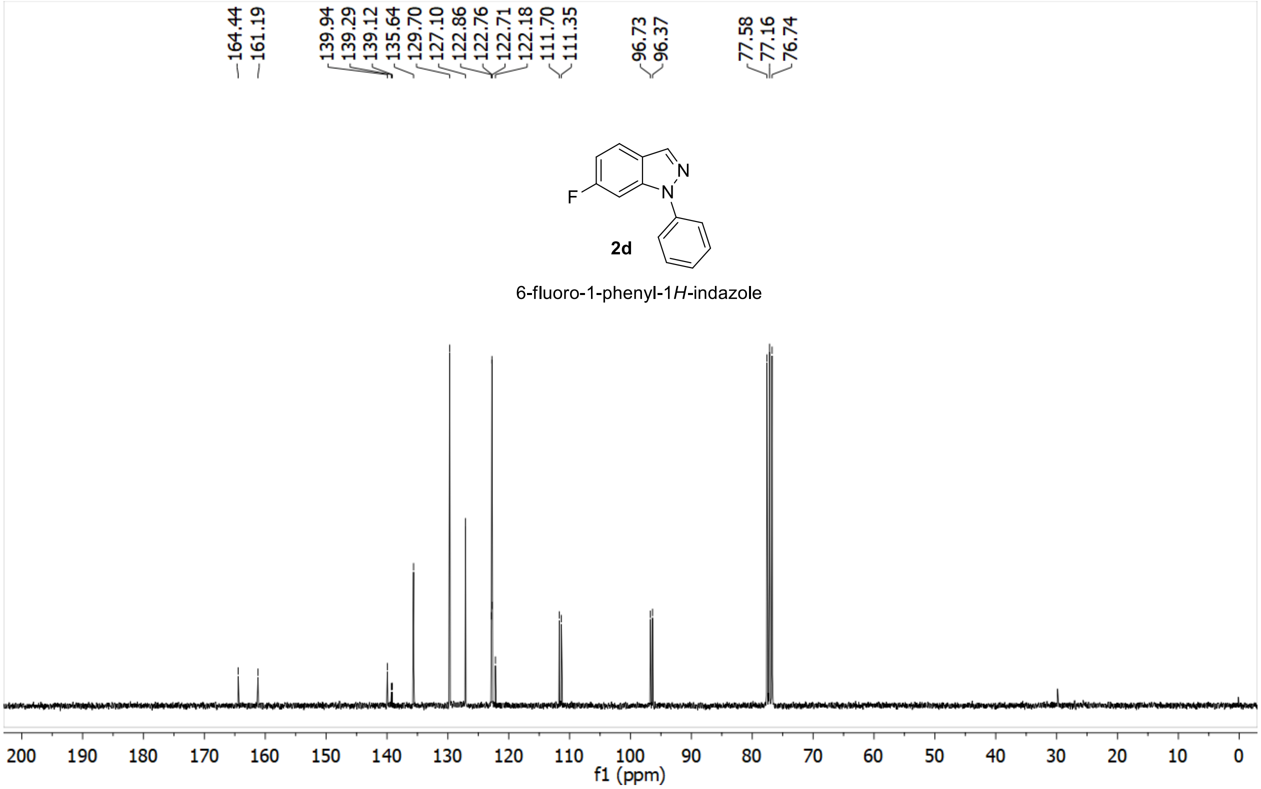

Figure S22. ^{13}C NMR spectrum of compound **2d** (75 MHz, $CDCl_3$).

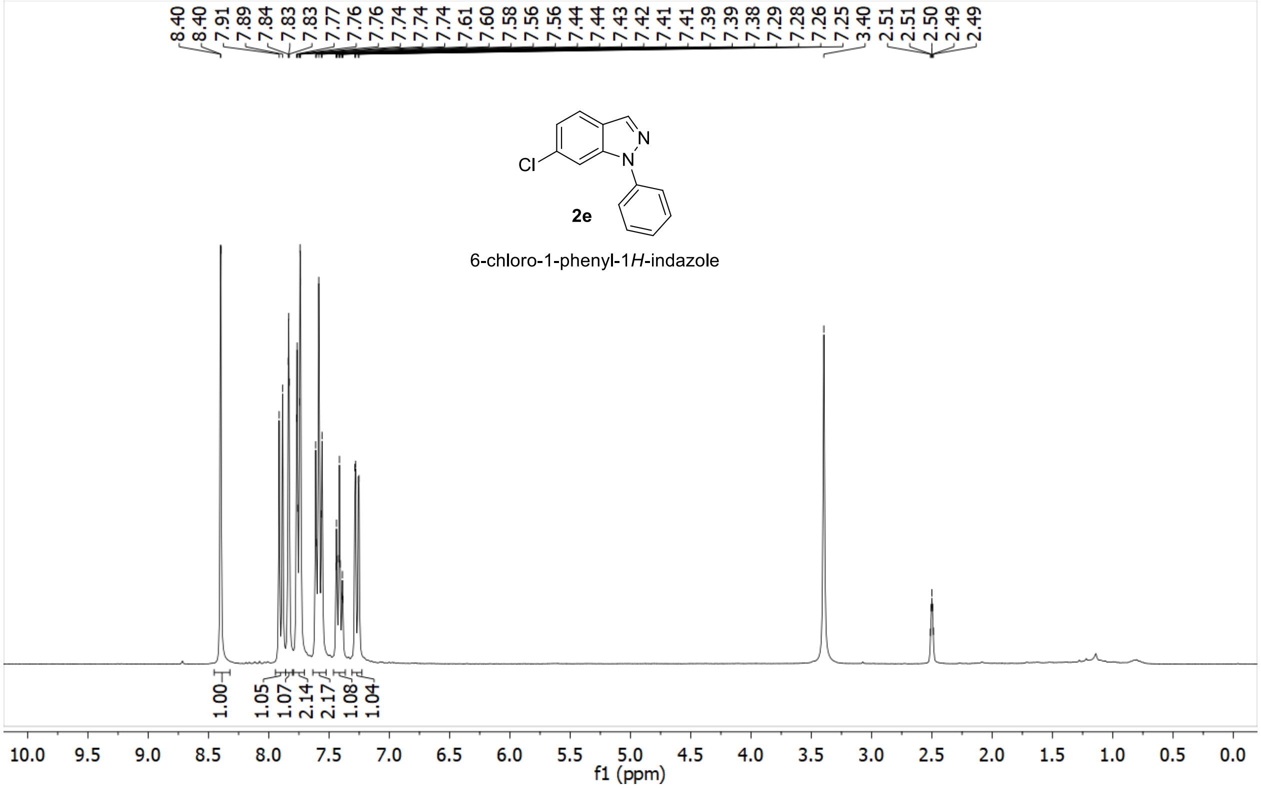

Figure S23. ^{1}H NMR spectrum of compound **2e** (300 MHz, DMSO-d_6).

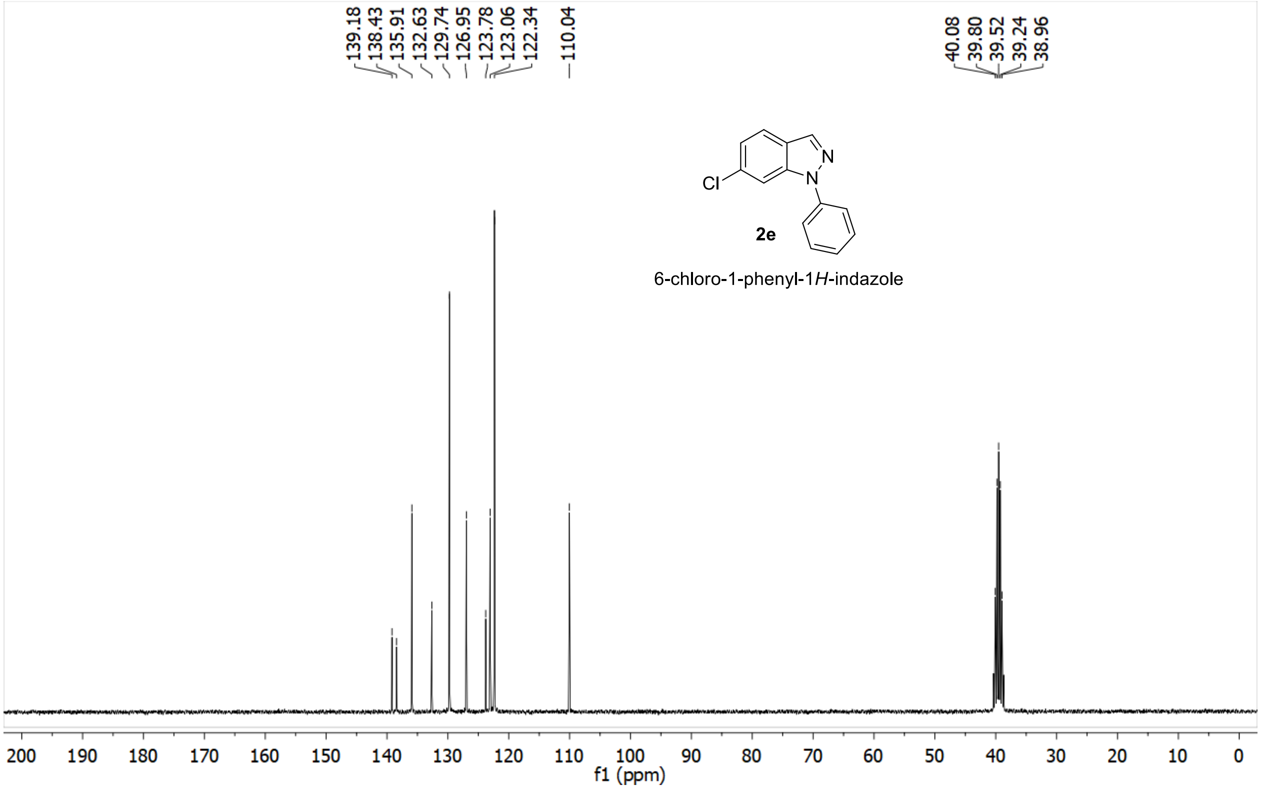

Figure S24. ^{13}C NMR spectrum of compound **2e** (75 MHz, DMSO-d_6).

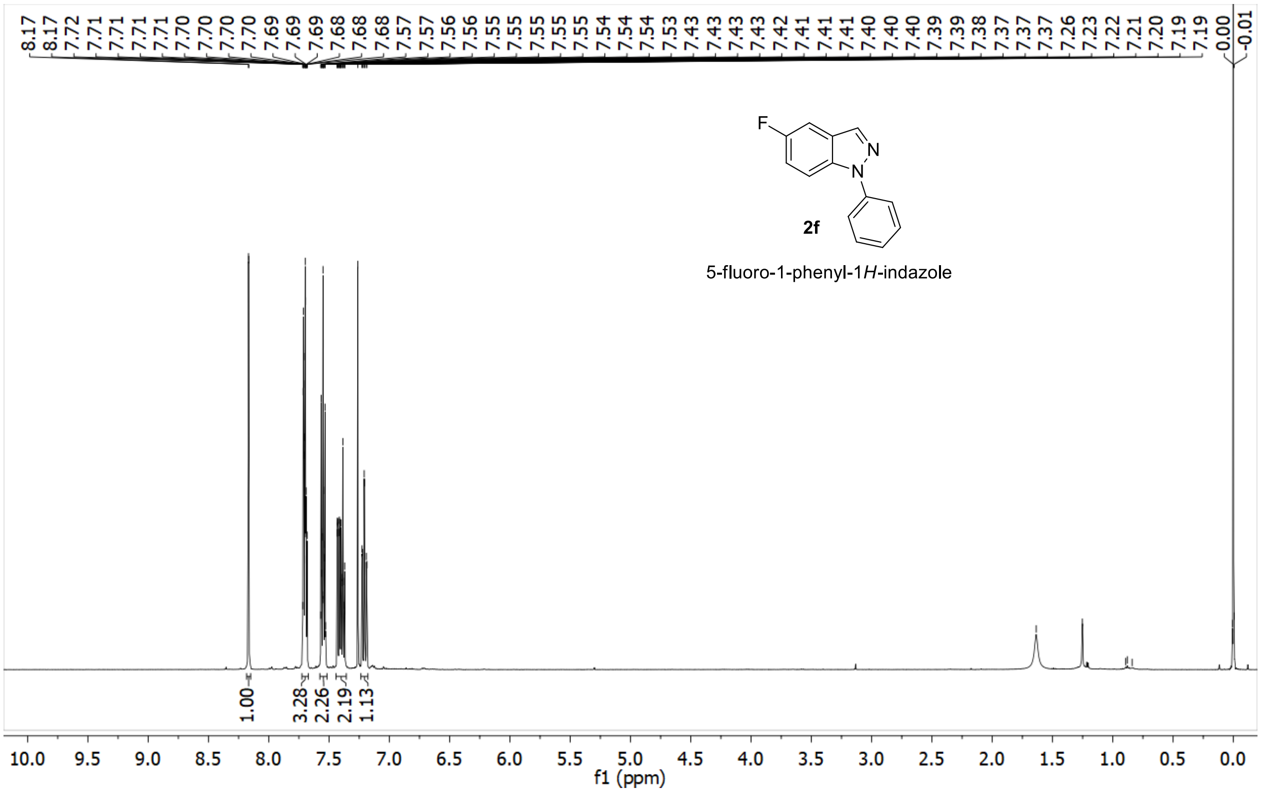

Figure S25. ^{1}H NMR spectrum of compound **2f** (500 MHz, $CDCl_3$).

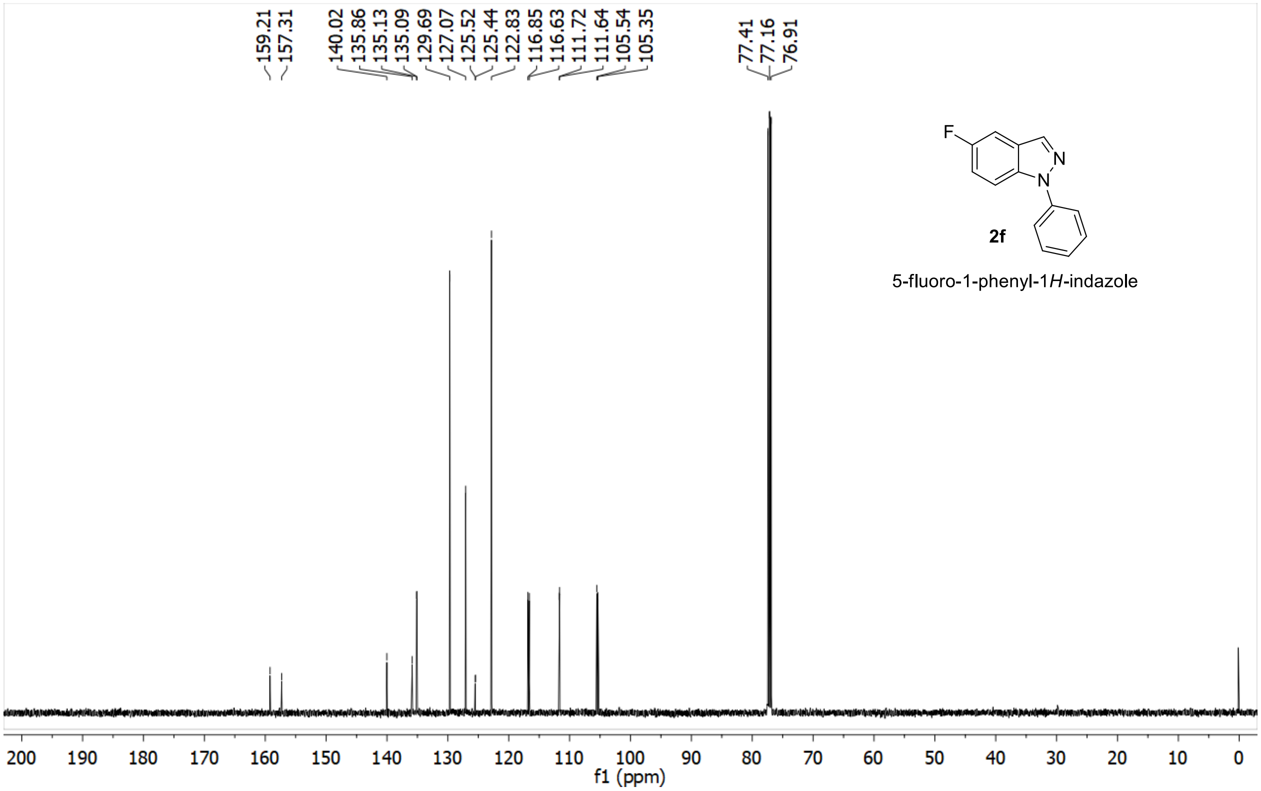

Figure S26. ^{13}C NMR spectrum of compound **2f** (125 MHz, $CDCl_3$).

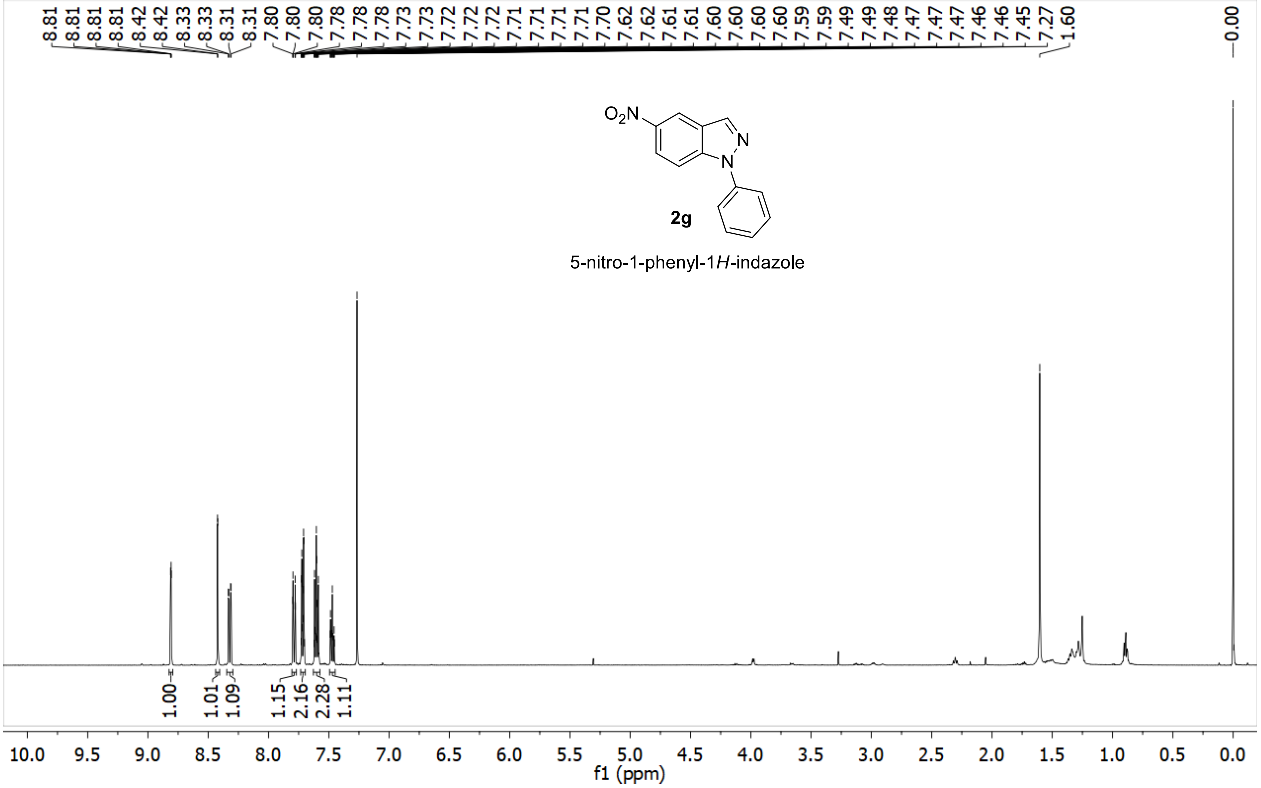

Figure S27. ^{1}H NMR spectrum of compound **2g** (500 MHz, $CDCl_3$).

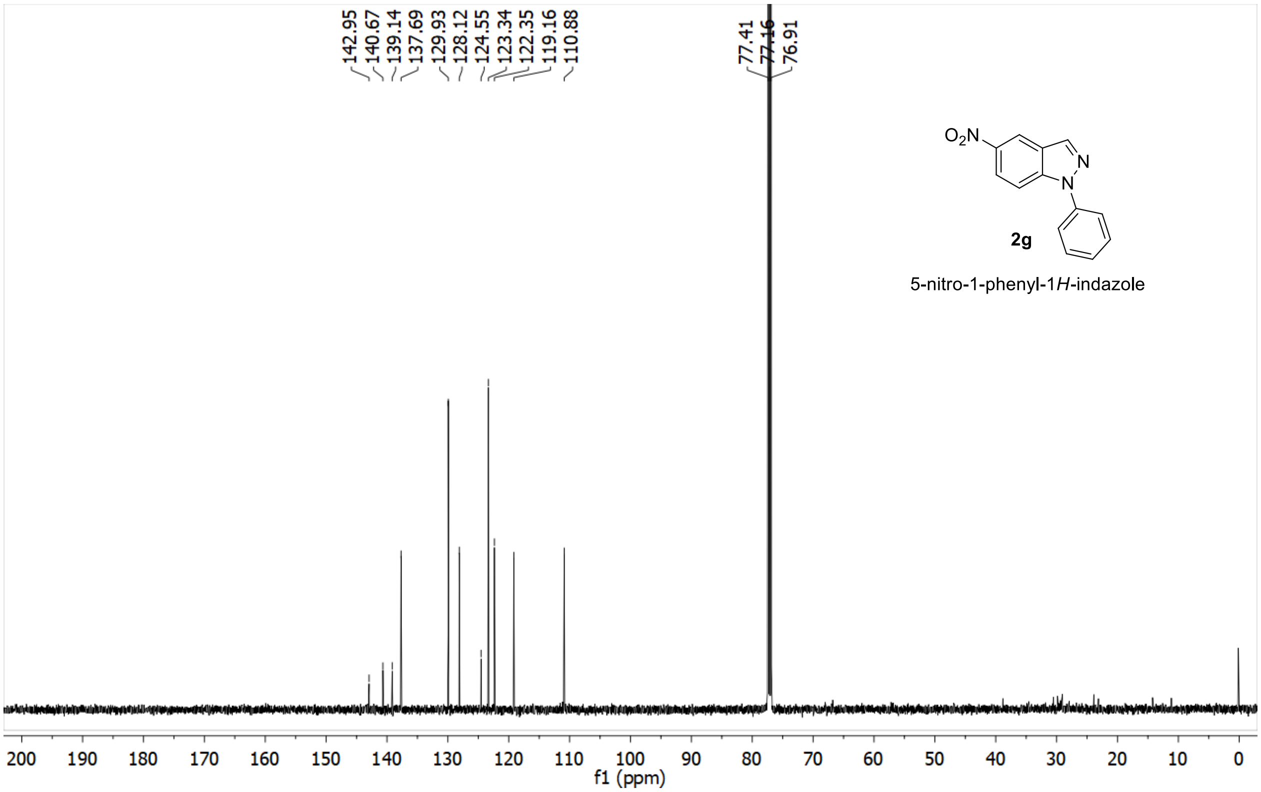

Figure S28. ^{13}C NMR spectrum of compound **2g** (125 MHz, $CDCl_3$).

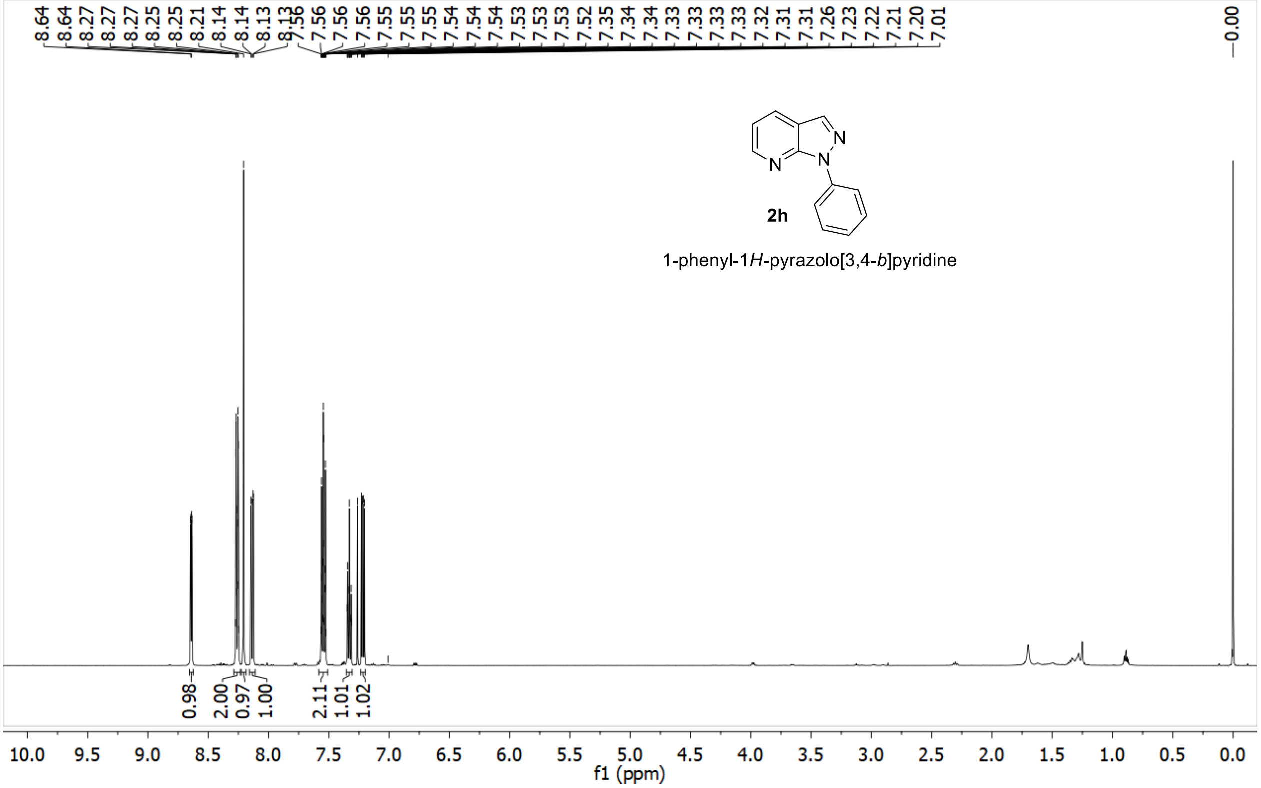

Figure S29. ^{1}H NMR spectrum of compound **2h** (500 MHz, $CDCl_3$).

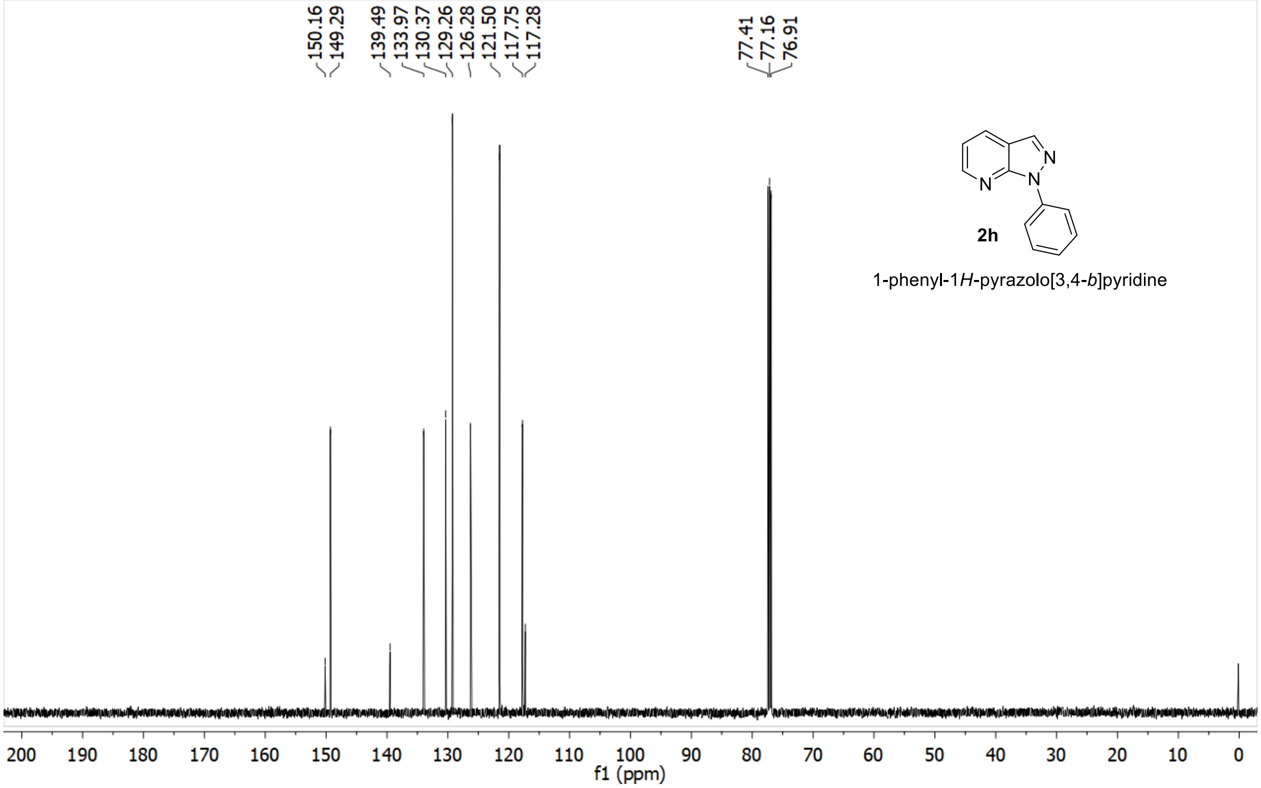

Figure S30. ^{13}C NMR spectrum of compound **2h** (125 MHz, $CDCl_3$).

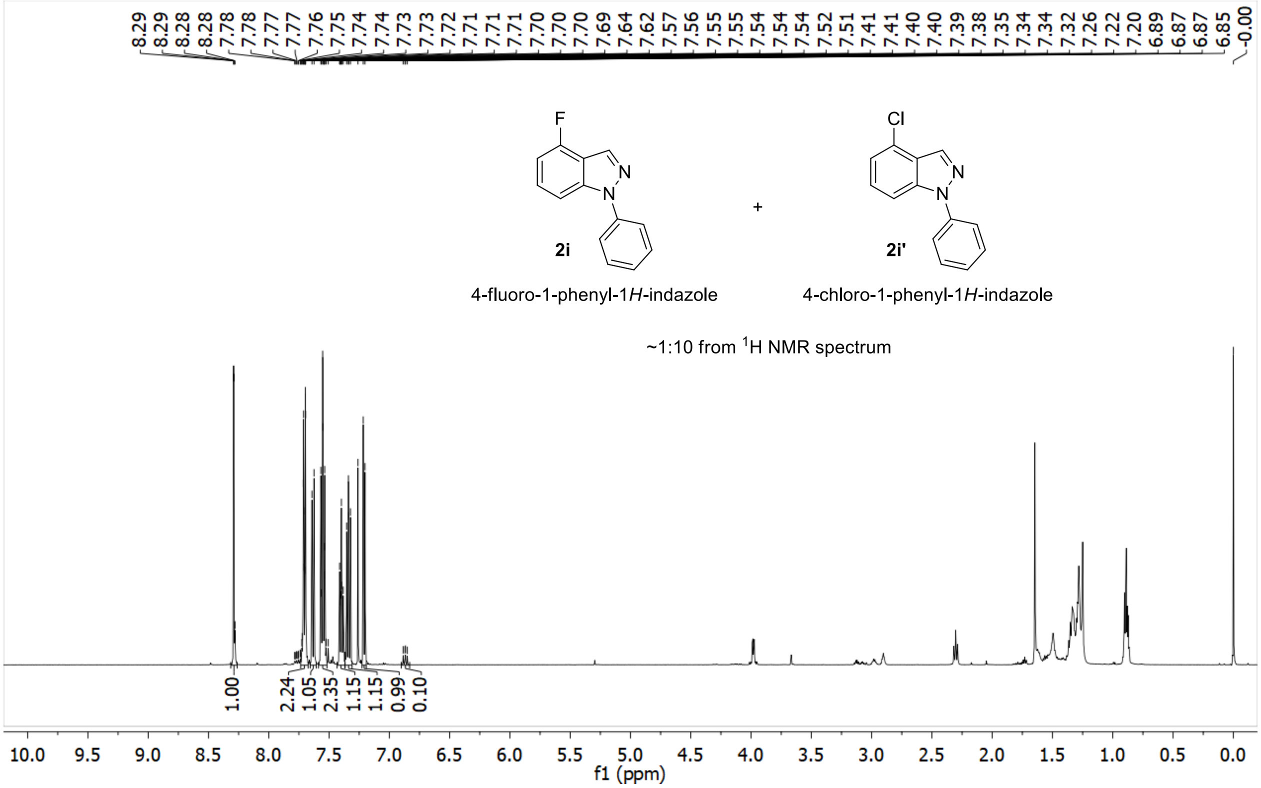

Figure S31. ^{1}H NMR spectrum of the mixture of compound **2i** and **2i'** (500 MHz, $CDCl_3$).

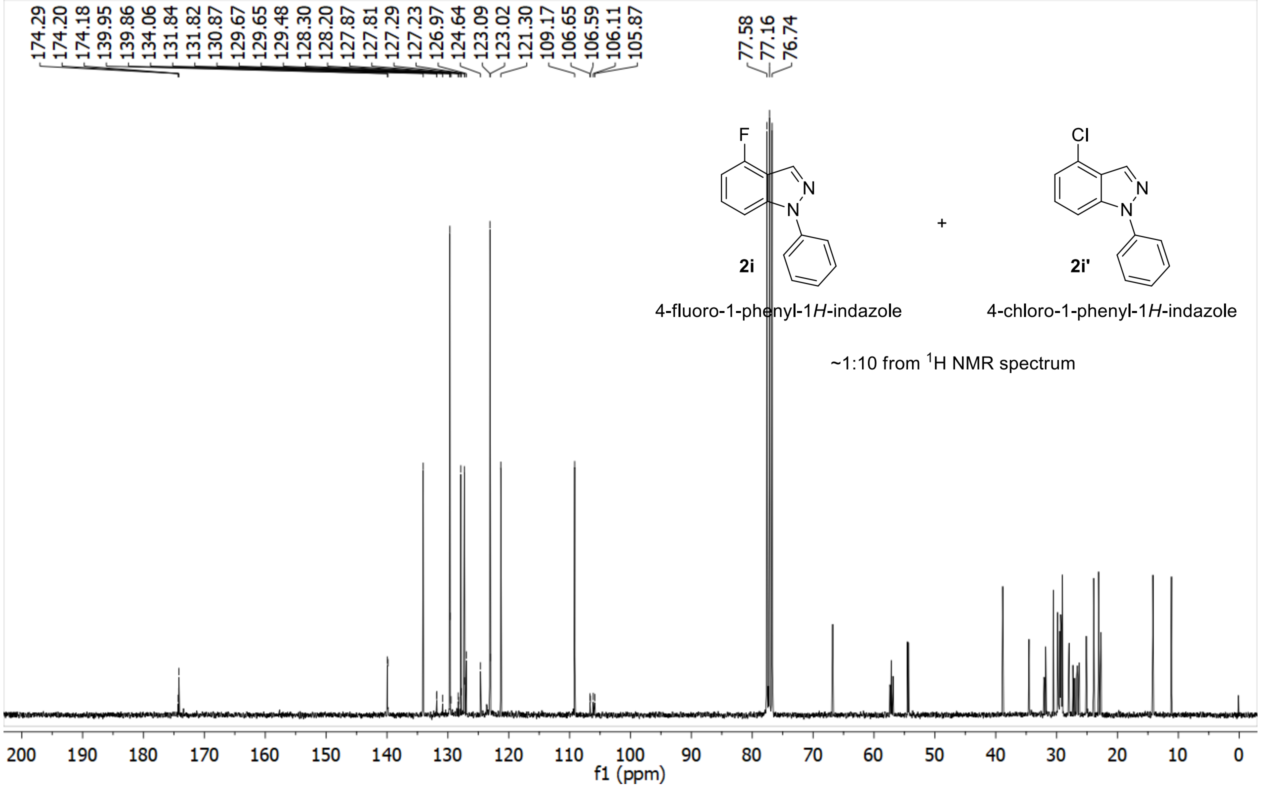

Figure S32. ^{13}C NMR spectrum of the mixture of compound **2i** and **2i'** (125 MHz, $CDCl_3$).

^{1}H and ^{13}C NMR spectra of *N*-thiazolyl-1*H*-indazoles

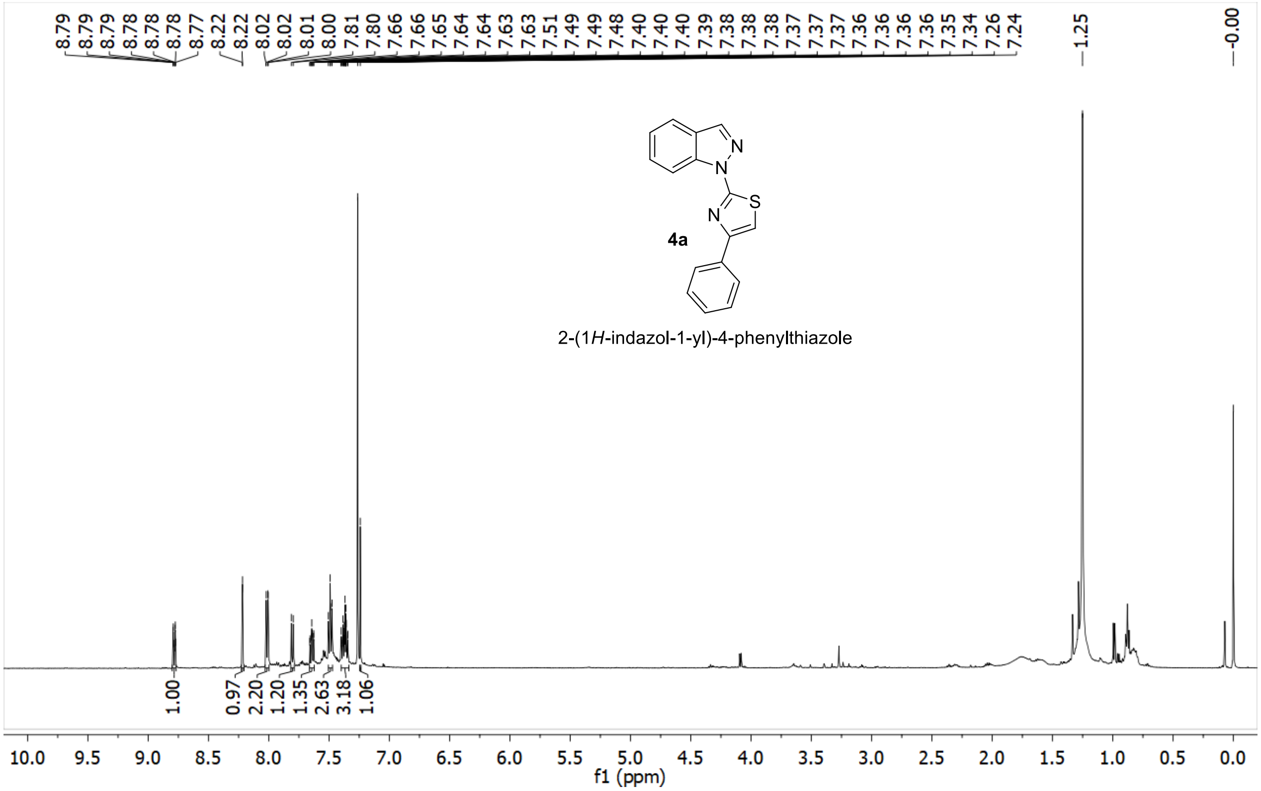

Figure S33. ^{1}H NMR spectrum of compound **4a** (500 MHz, $CDCl_3$).

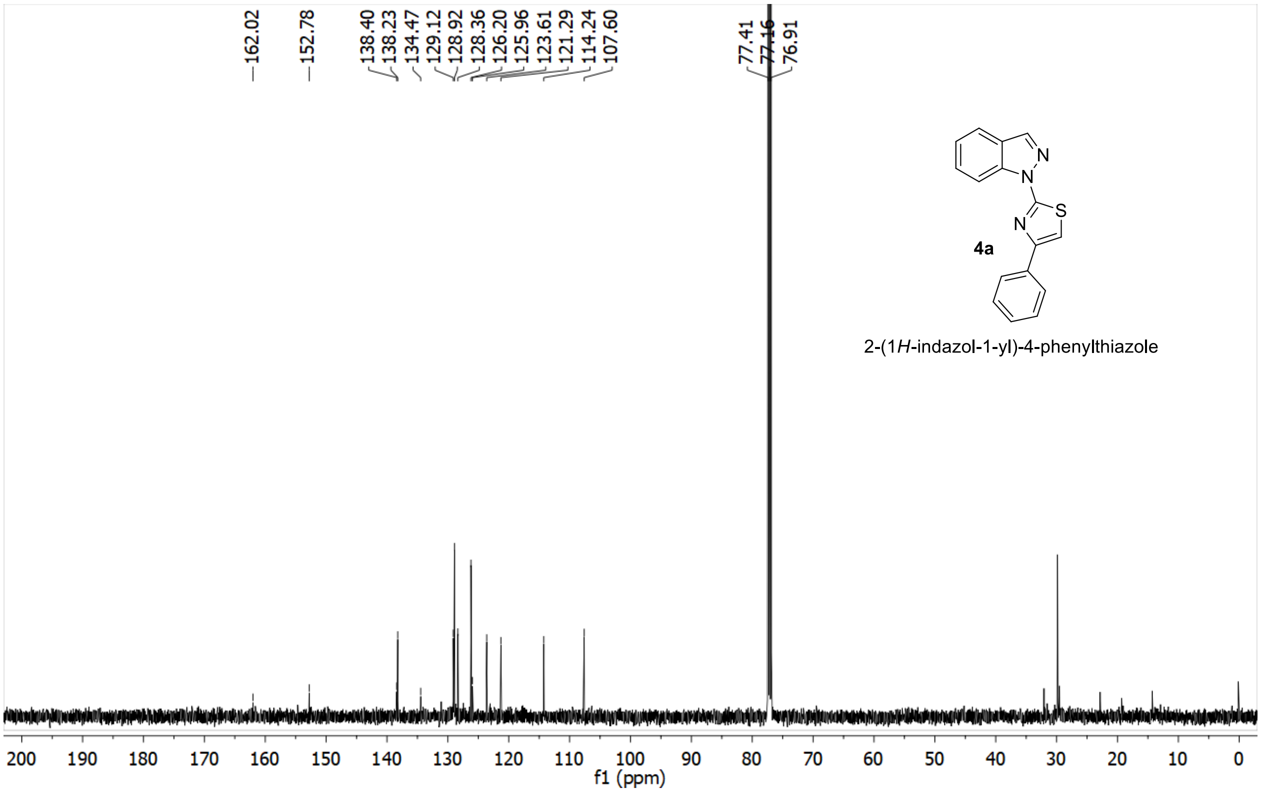

Figure S34. ^{13}C NMR spectrum of compound **4a** (125 MHz, $CDCl_3$).

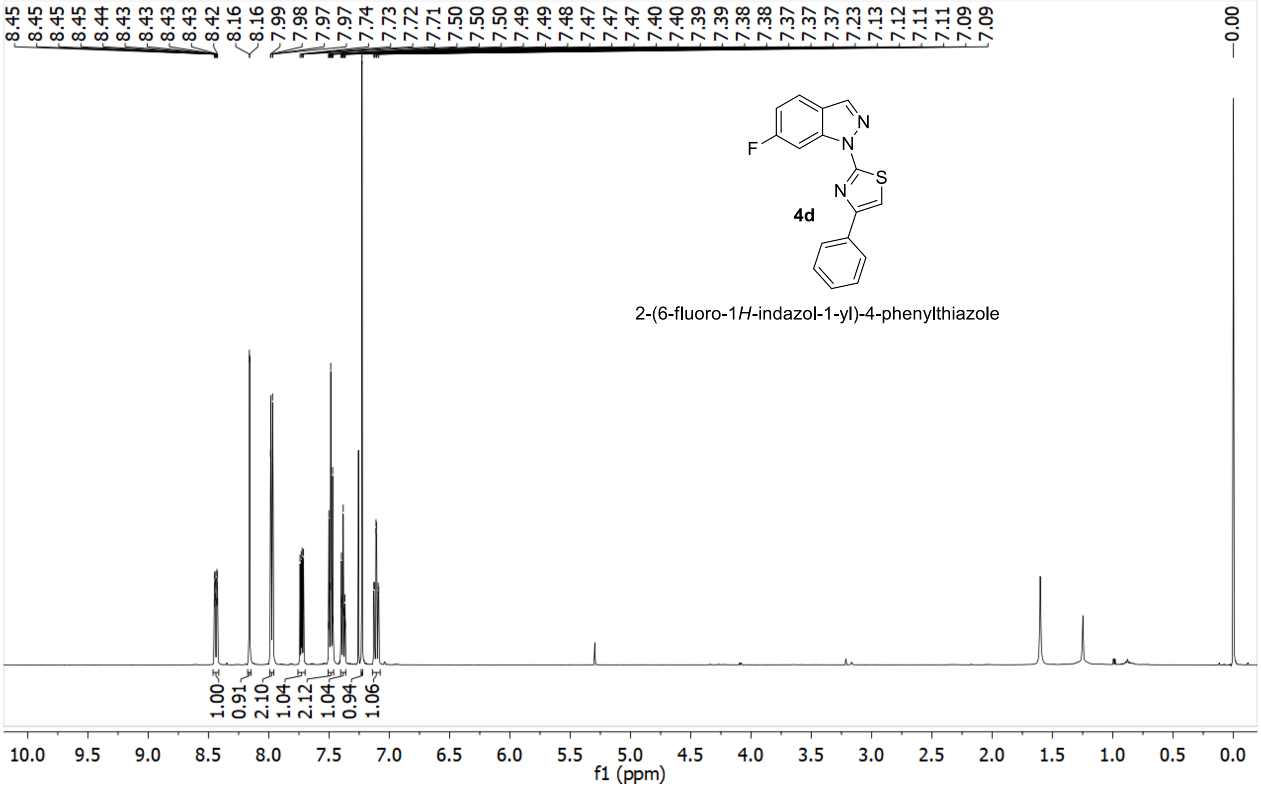

Figure S35. ^{1}H NMR spectrum of compound **4d** (500 MHz, $CDCl_3$).

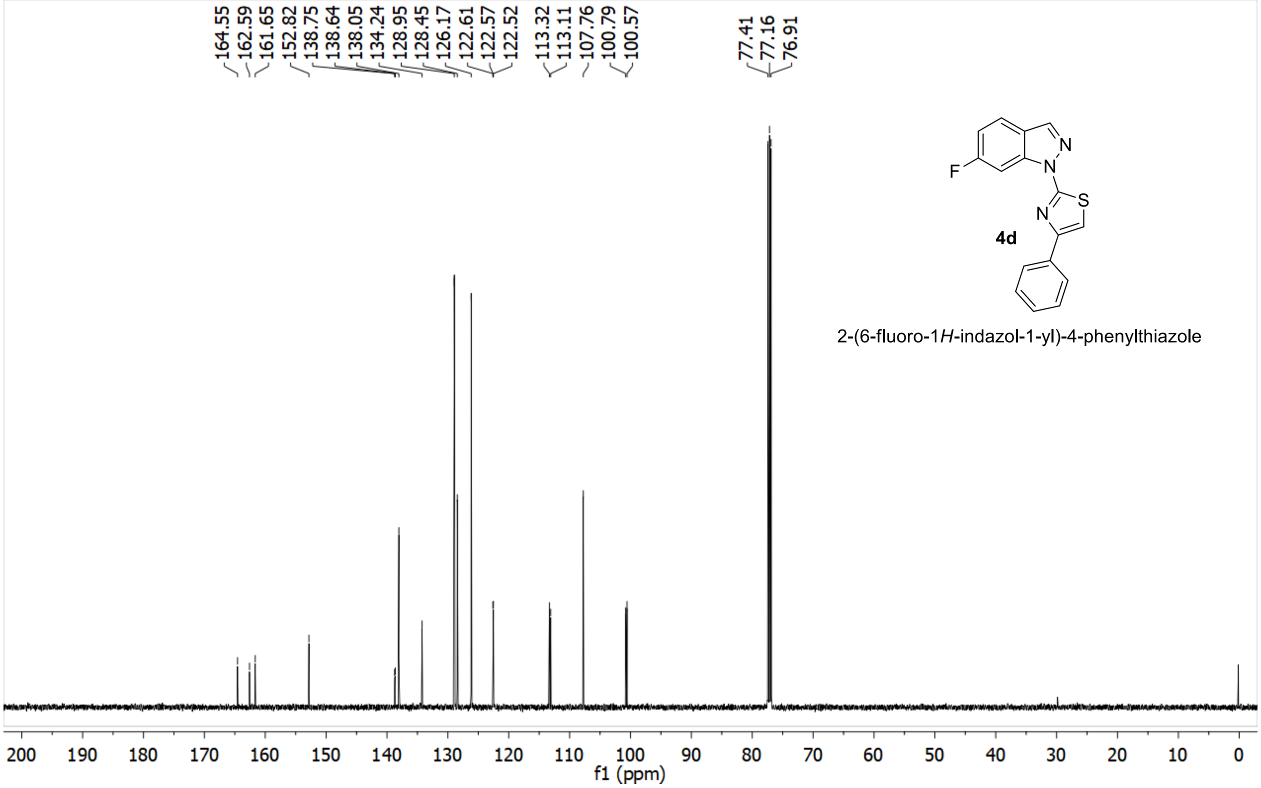

Figure S36. ^{13}C NMR spectrum of compound **4d** (125 MHz, $CDCl_3$).

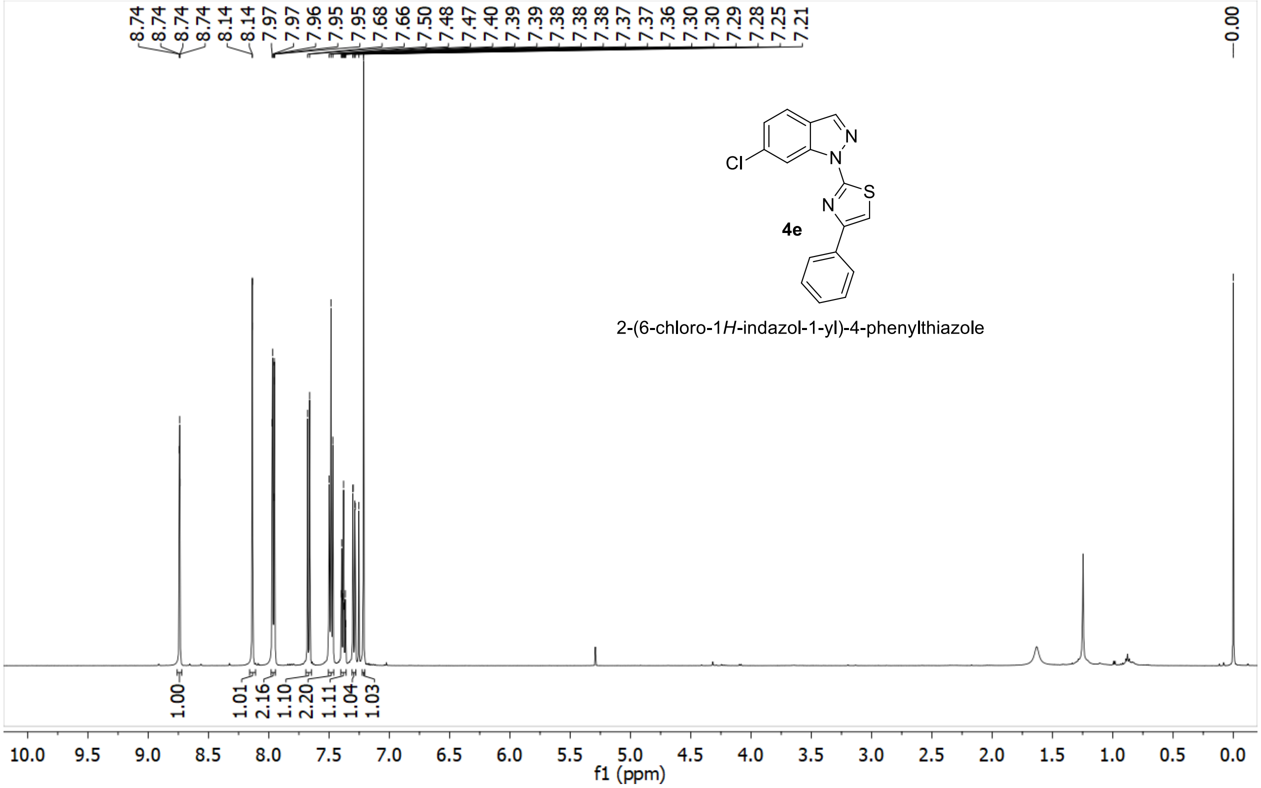

Figure S37. ^{1}H NMR spectrum of compound **4e** (500 MHz, $CDCl_3$).

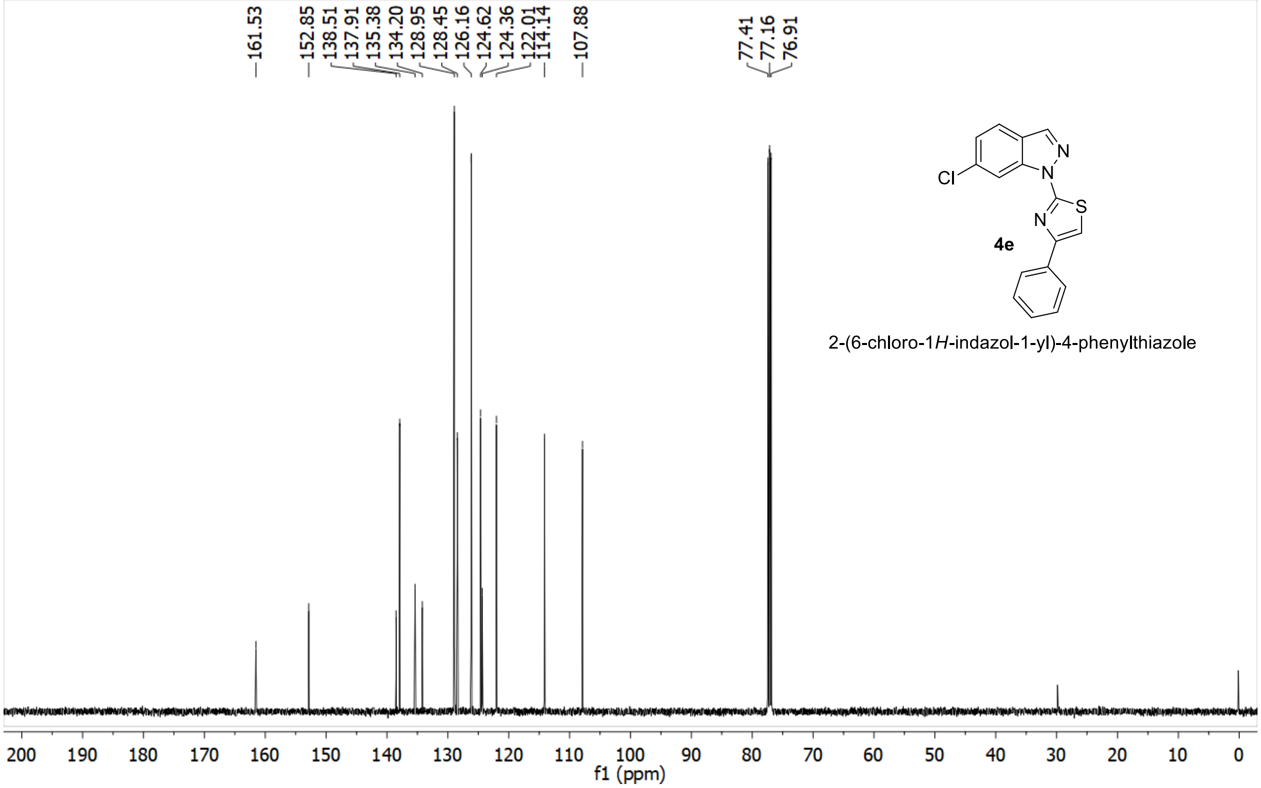

Figure S38. ^{13}C NMR spectrum of compound **4e** (125 MHz, $CDCl_3$).

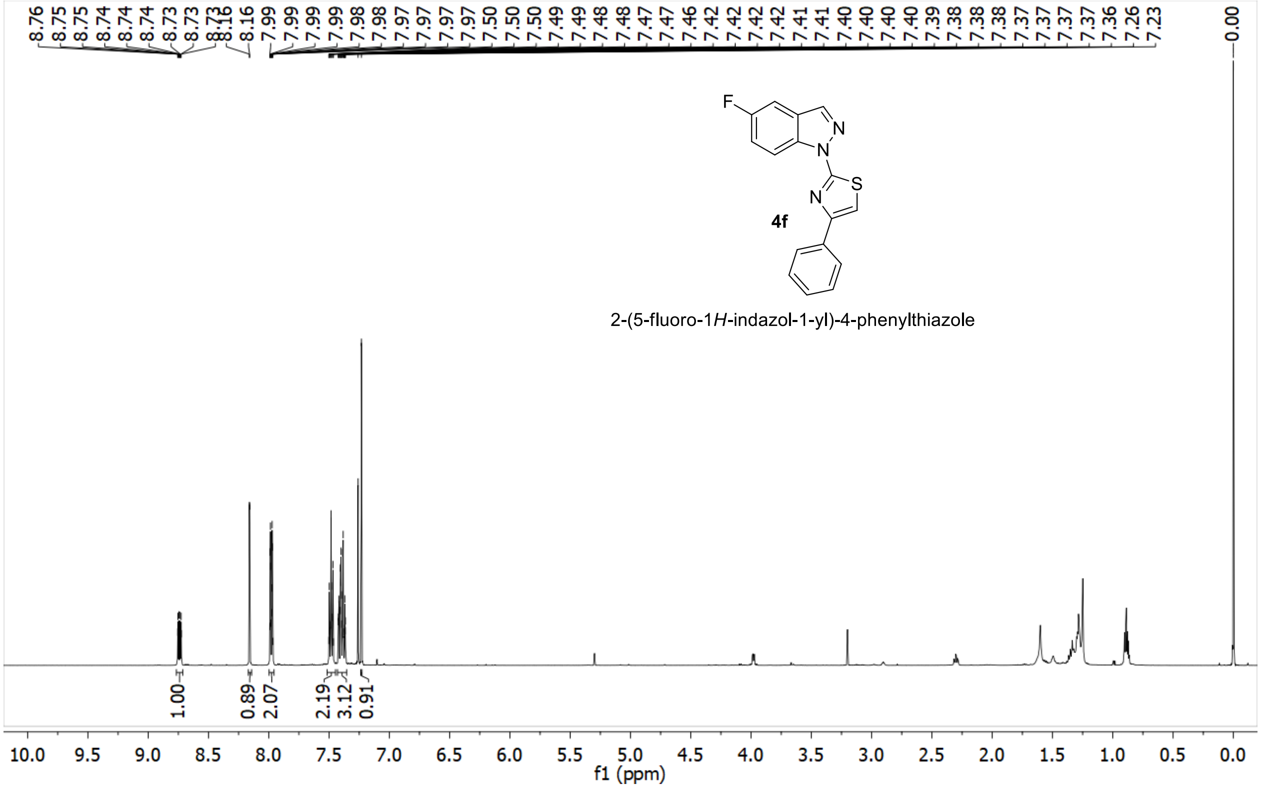

Figure S39. ^{1}H NMR spectrum of compound **4f** (500 MHz, $CDCl_3$).

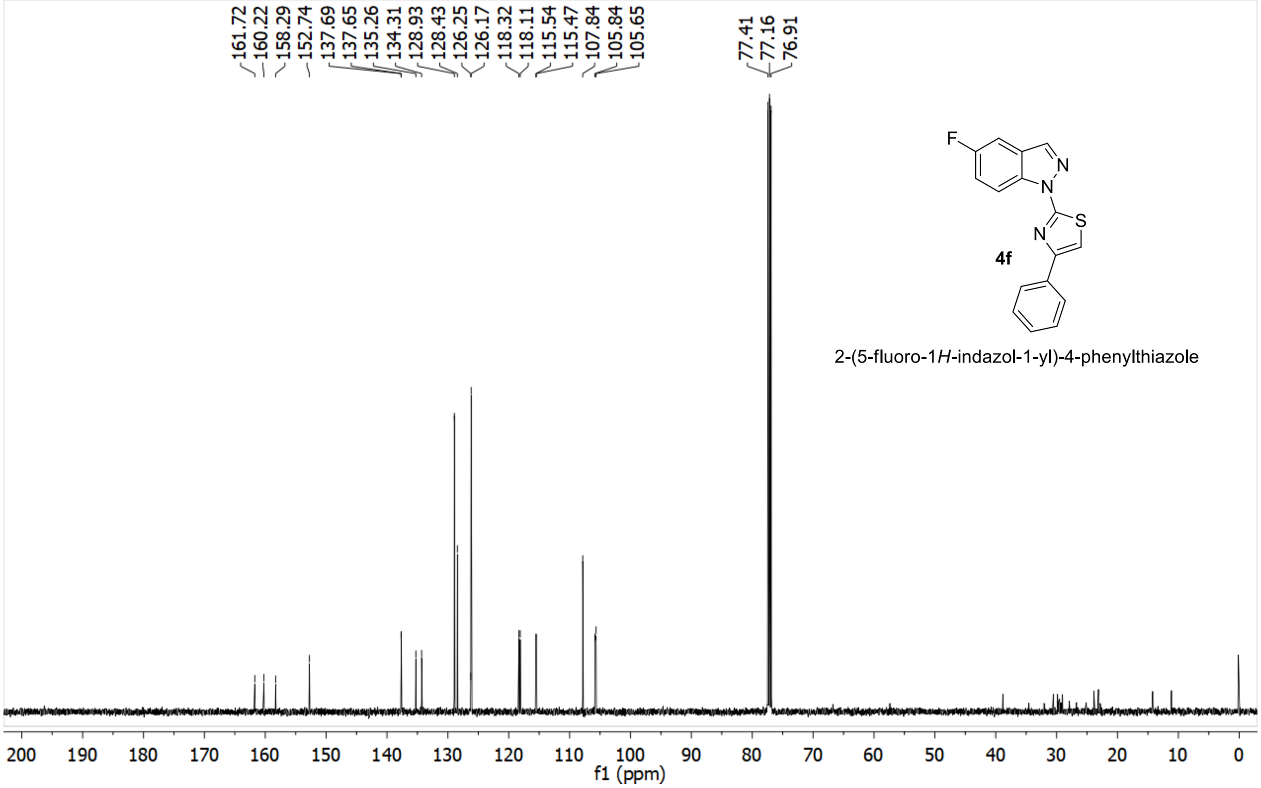

Figure S40. ^{13}C NMR spectrum of compound **4f** (125 MHz, $CDCl_3$).

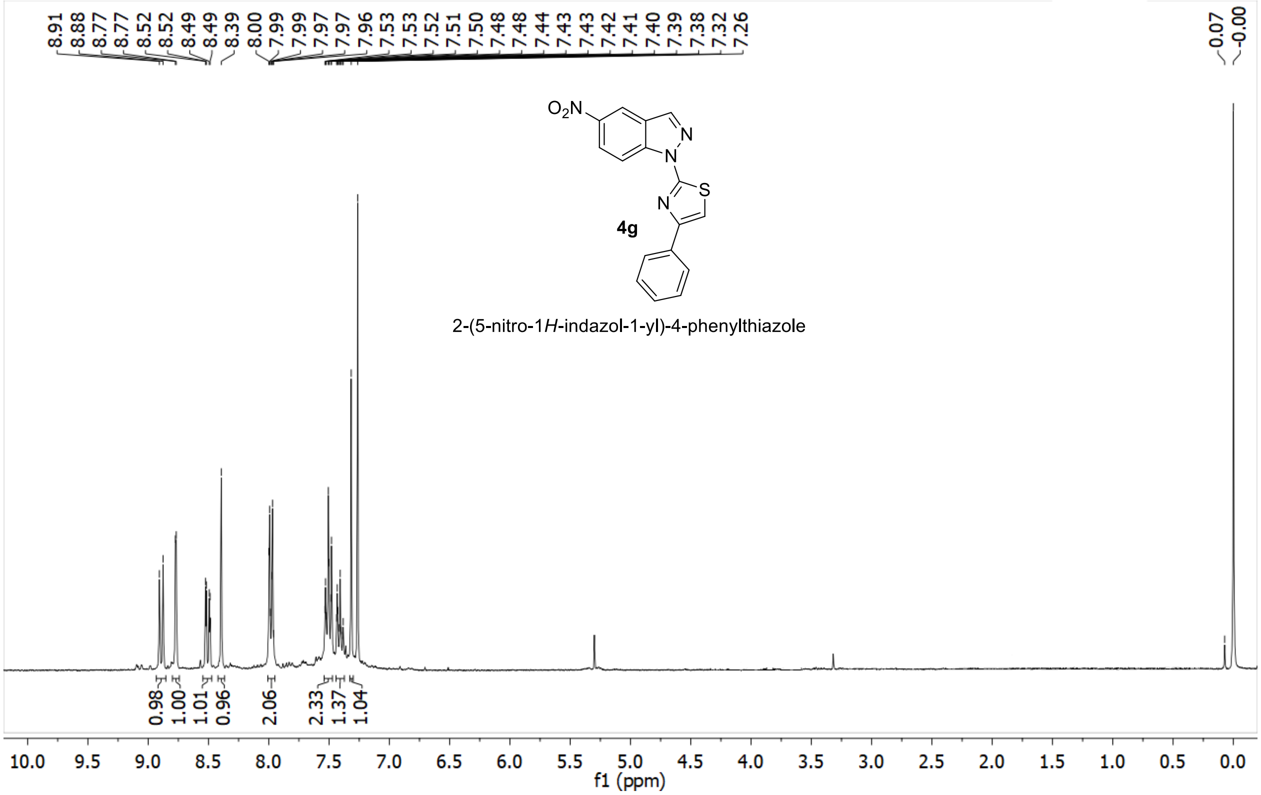

Figure S41. ^{1}H NMR spectrum of compound **4g** (300 MHz, $CDCl_3$).

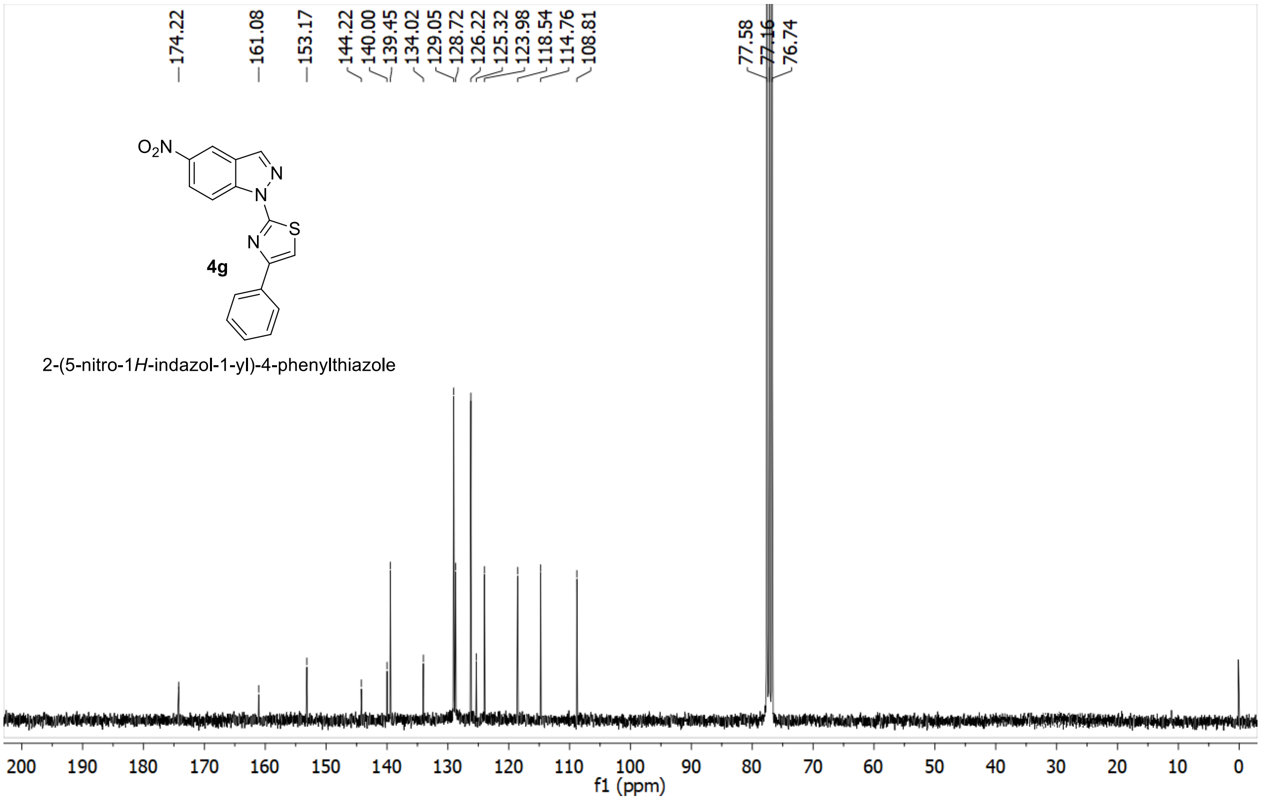

Figure S42. ^{13}C NMR spectrum of compound **4g** (75 MHz, $CDCl_3$).

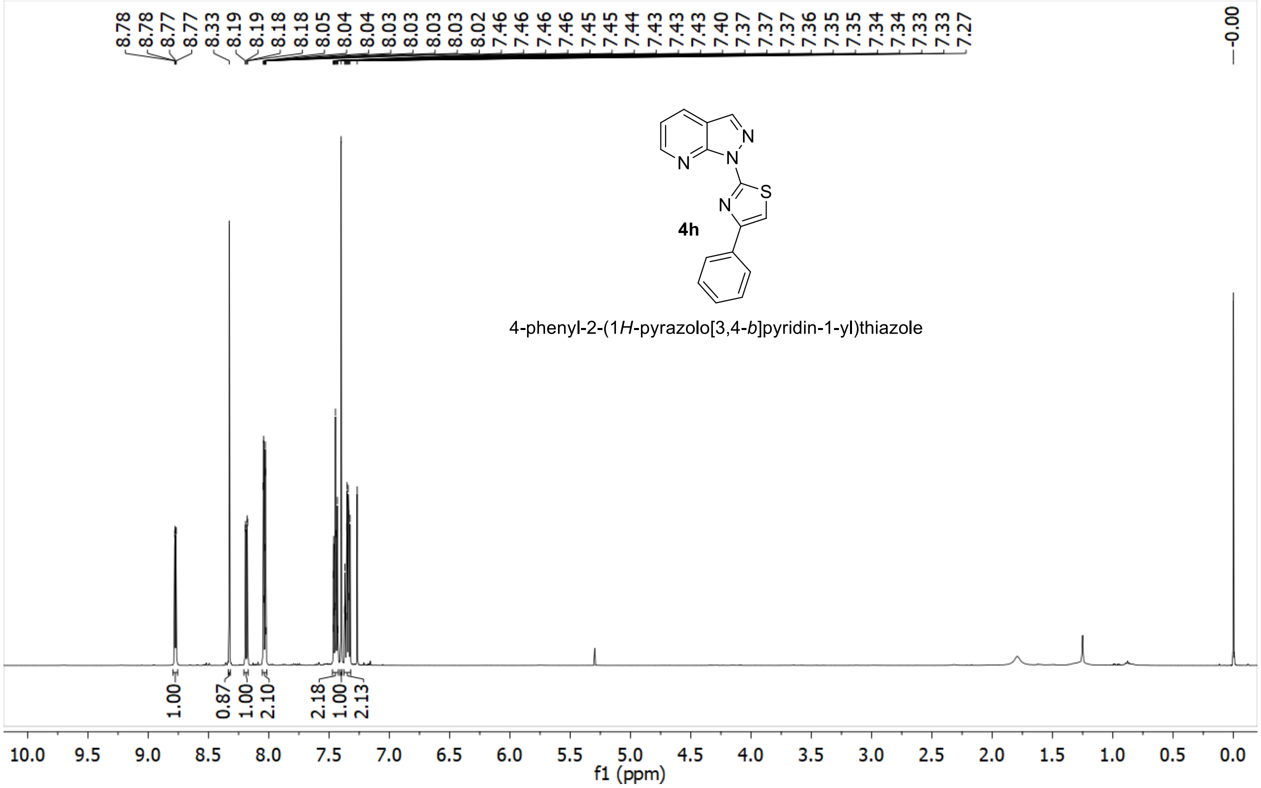

Figure S43. ^{1}H NMR spectrum of compound **4h** (500 MHz, $CDCl_3$).

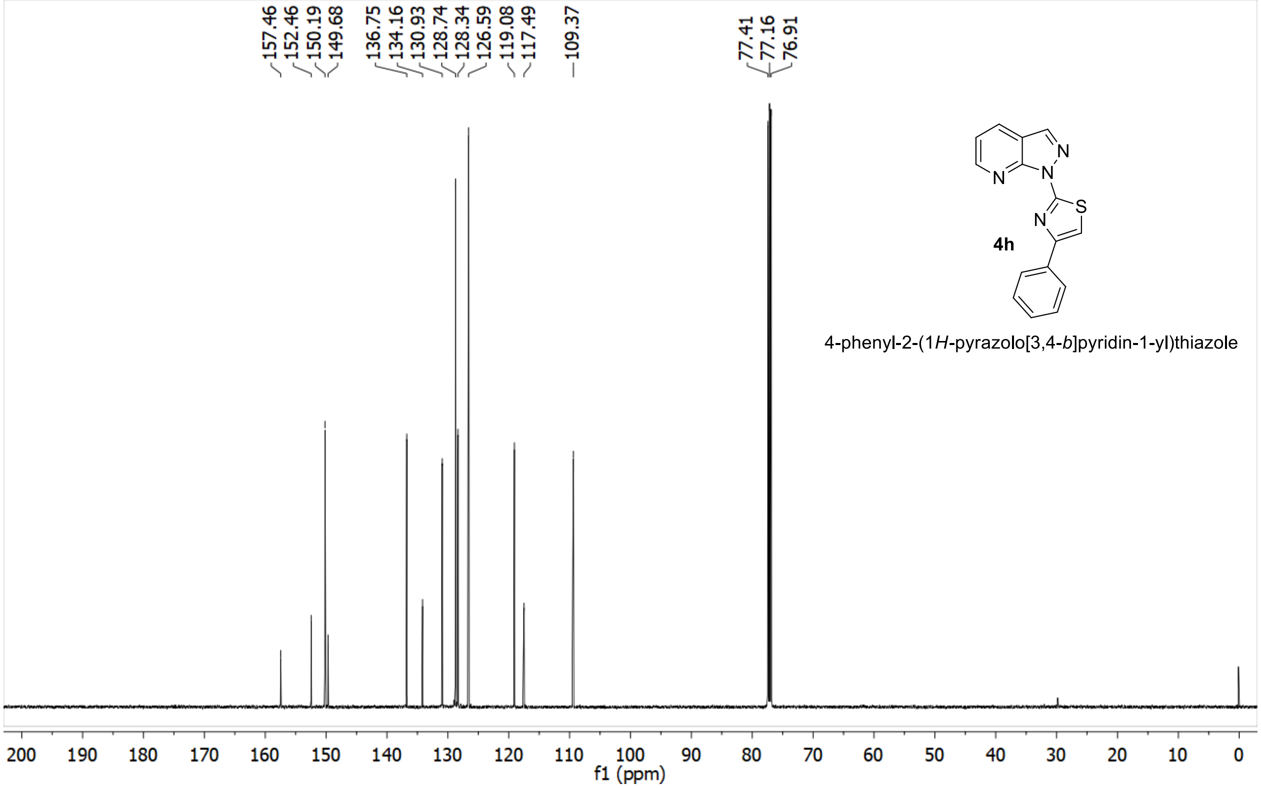

Figure S44. ^{13}C NMR spectrum of compound **4h** (125 MHz, $CDCl_3$).

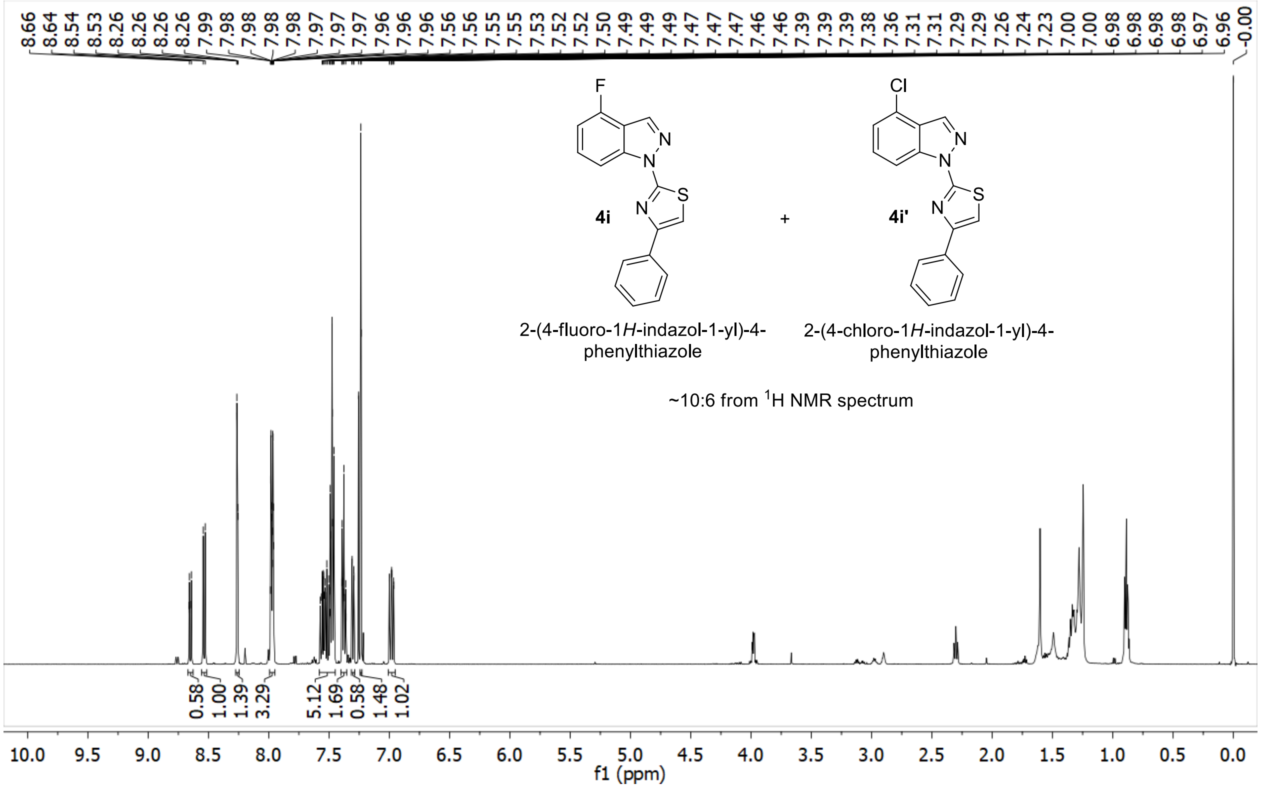

Figure S45. ^{1}H NMR spectrum of the mixture of compounds **4i** and **4i'** (500 MHz, $CDCl_3$).

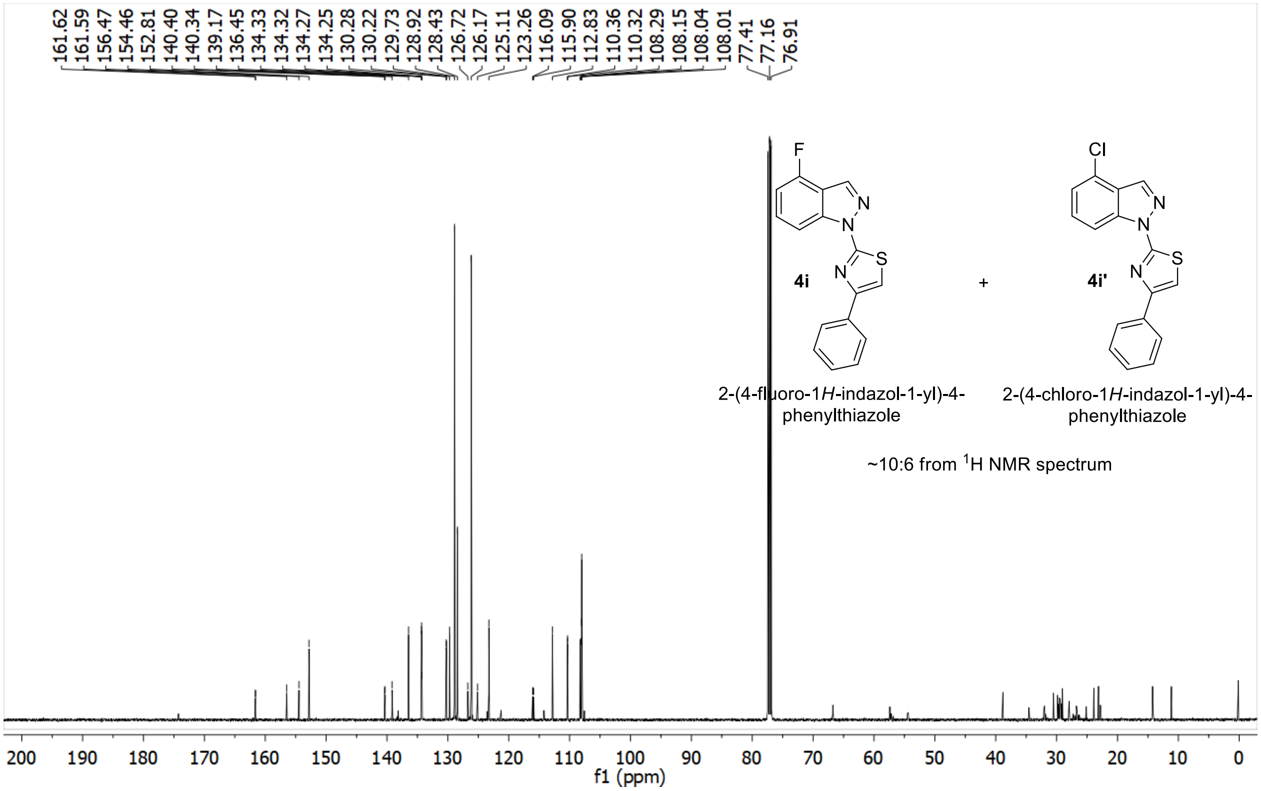

Figure S46. ^{13}C NMR spectrum of the mixture of compounds **4i** and **4i'** (175 MHz, $CDCl_3$).

HRMS spectra of *N*-phenylhydrazones 1a–i

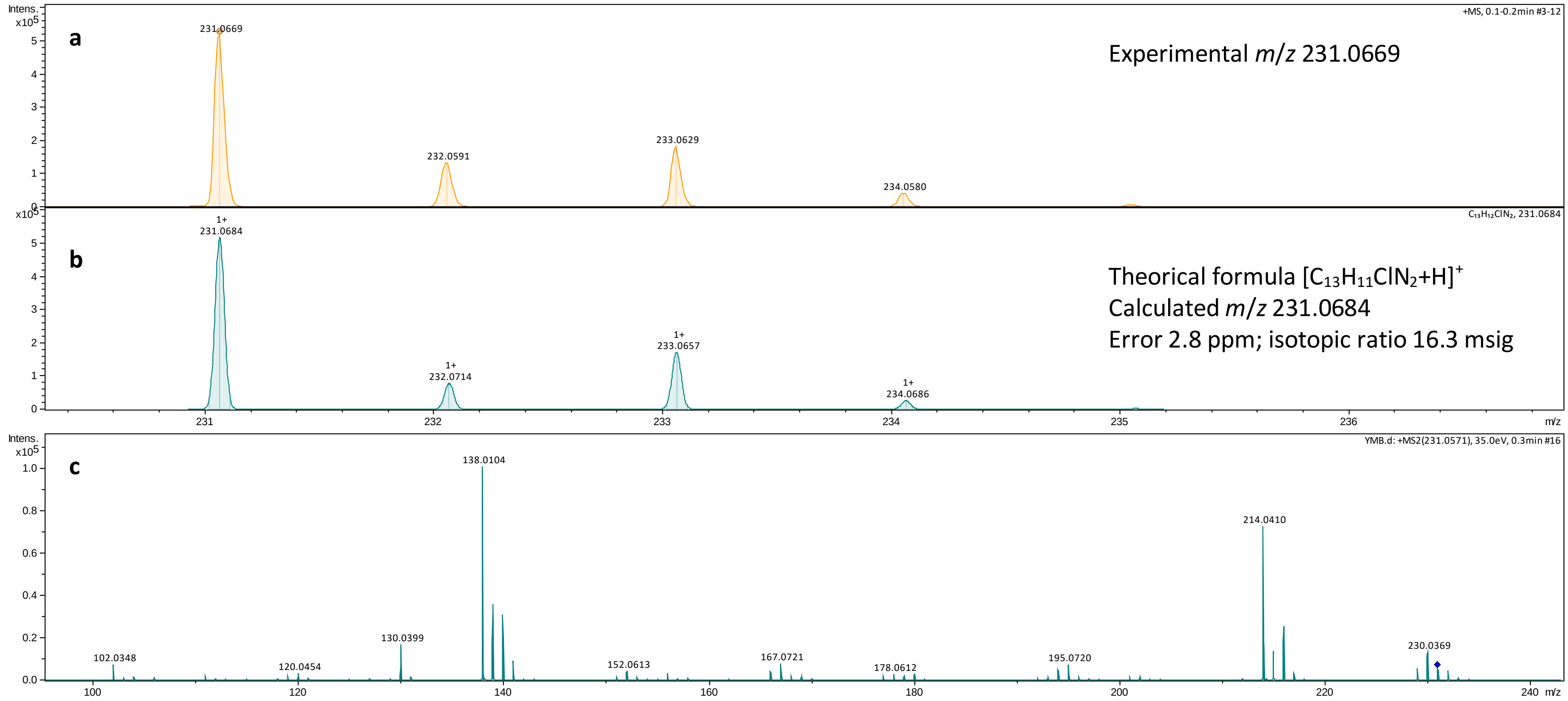

Figure S47. The HRMS analysis of compound **1a**. The experimental spectrum (**a**) and the simulated spectrum (**b**), both expanded between 230.3 and 236.9 Da highlighting the exact mass and isotopic ratio; the analysis in MS–MS mode (**c**) (fragmentation pathway).

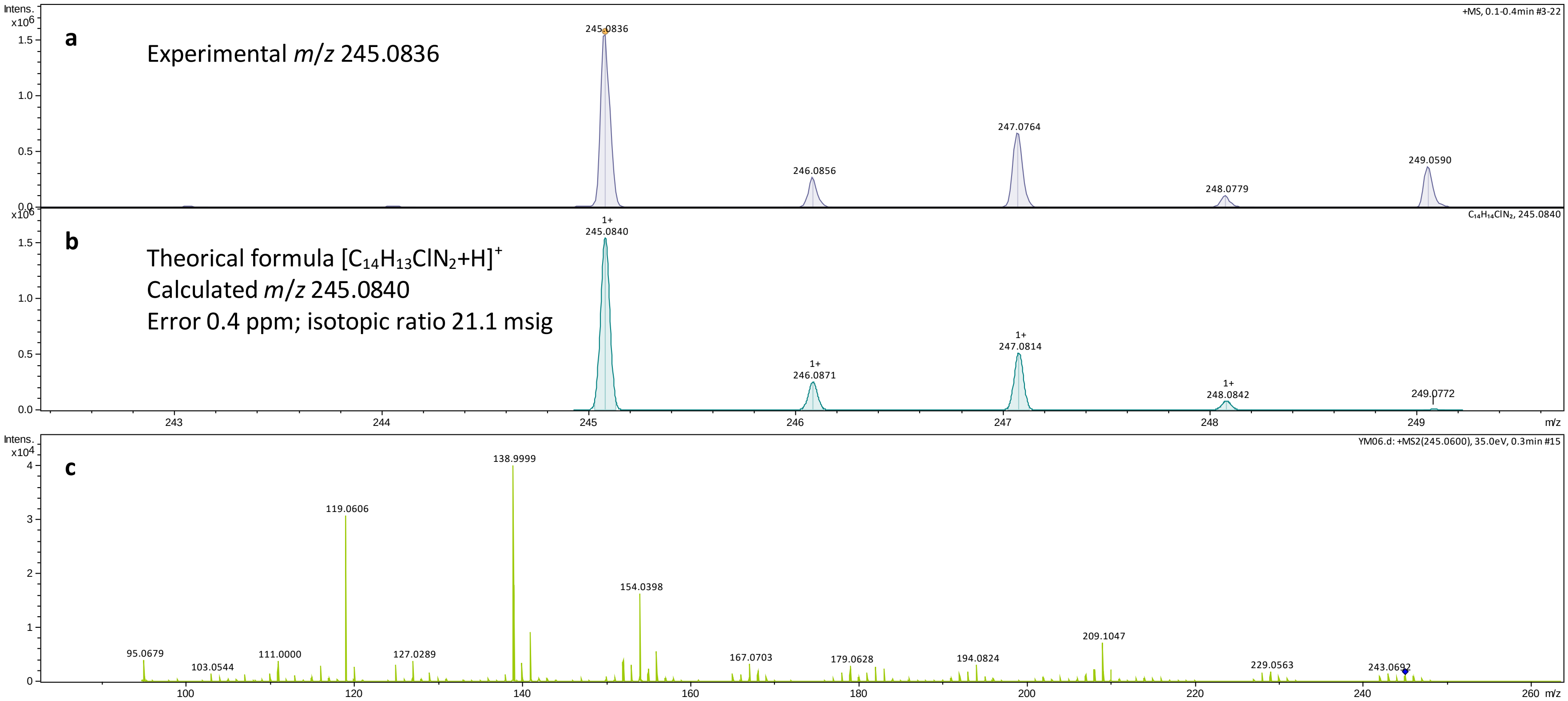

Figure S48. The HRMS analysis of compound **1b**. The experimental spectrum (**a**) and the simulated spectrum (**b**), both expanded between 242.4 and 249.7 Da highlighting the exact mass and isotopic ratio; the analysis in MS–MS mode (**c**) (fragmentation pathway).

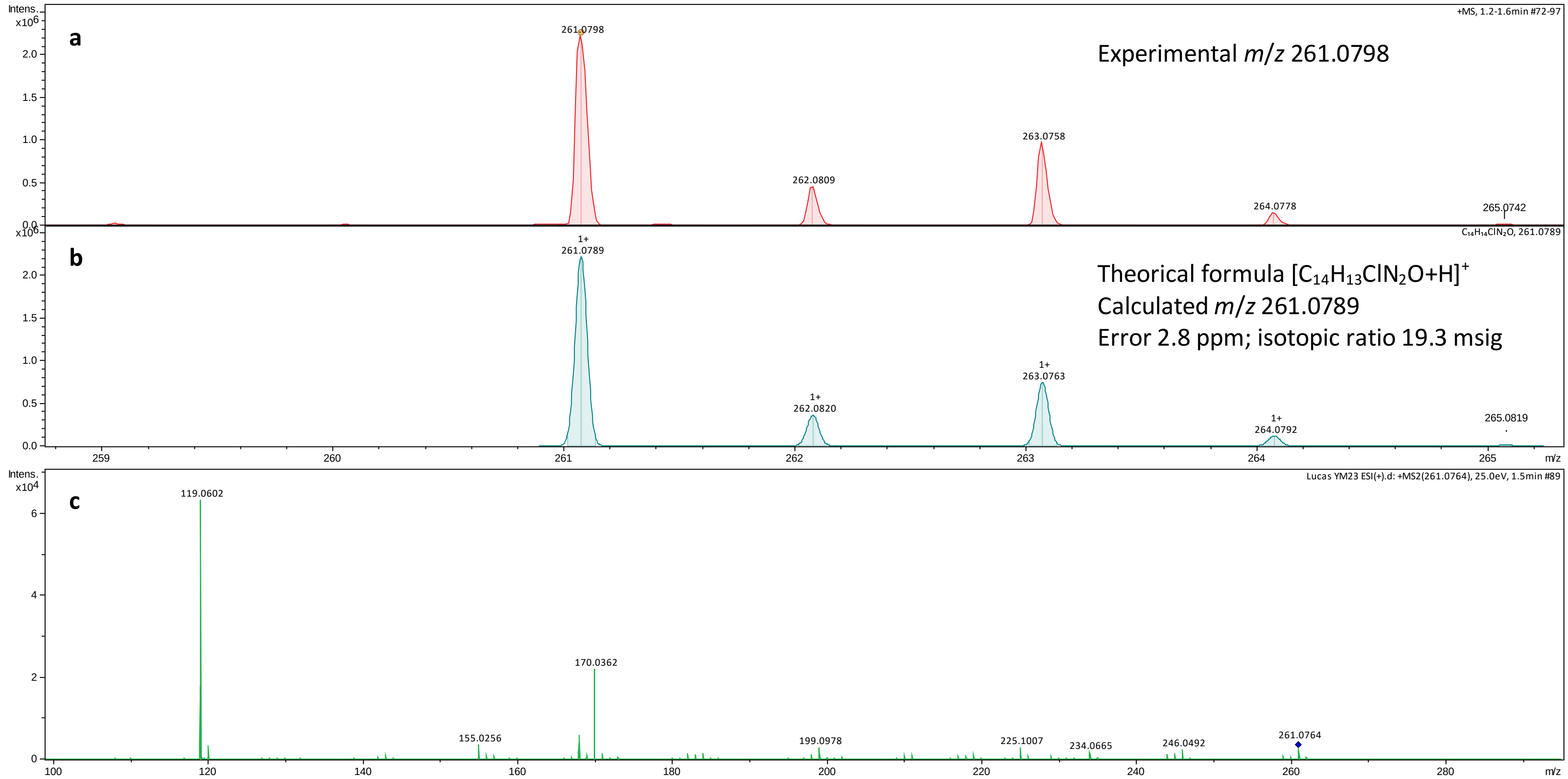

Figure S49. The HRMS analysis of compound **1c**. The experimental spectrum (**a**) and the simulated spectrum (**b**), both expanded between 242.4 and 249.7 Da highlighting the exact mass and isotopic ratio; the analysis in MS–MS mode (**c**) (fragmentation pathway).

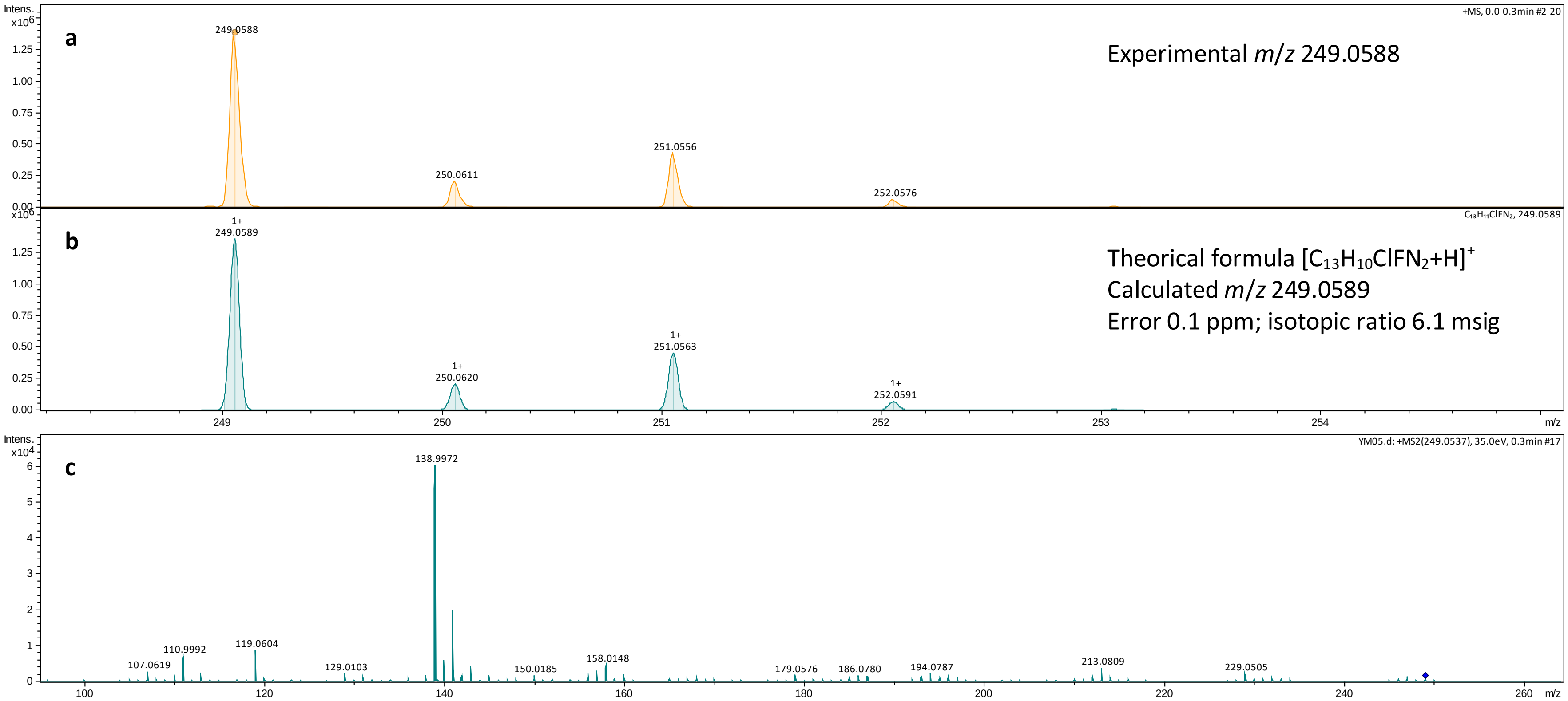

Figure S50. The HRMS analysis of compound **1d**. The experimental spectrum (**a**) and the simulated spectrum (**b**), both expanded between 248.2 and 255.1 Da highlighting the exact mass and isotopic ratio; the analysis in MS–MS mode (**c**) (fragmentation pathway).

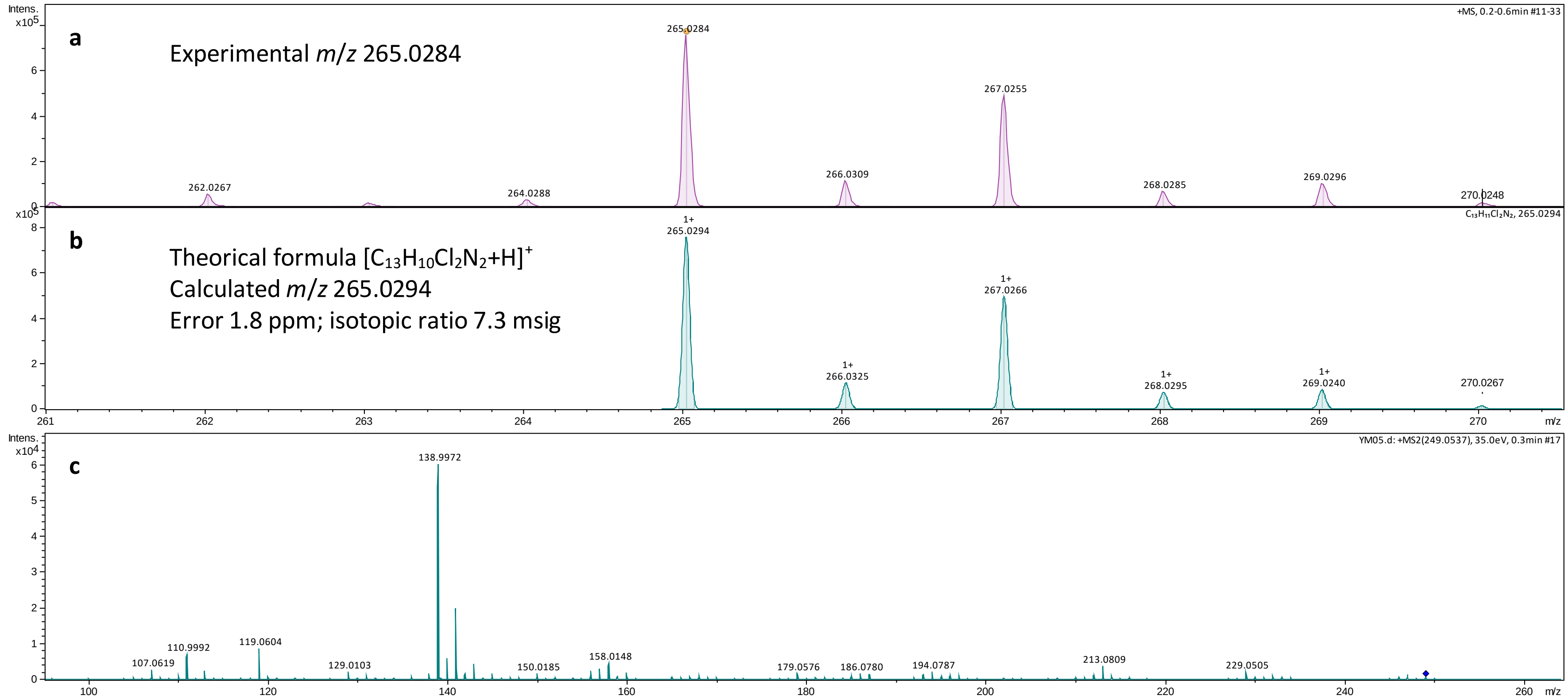

Figure S51. The HRMS analysis of compound **1e**. The experimental spectrum (**a**) and the simulated spectrum (**b**), both expanded between 261.0 and 270.5 Da highlighting the exact mass and isotopic ratio; the analysis in MS–MS mode (**c**) (fragmentation pathway).

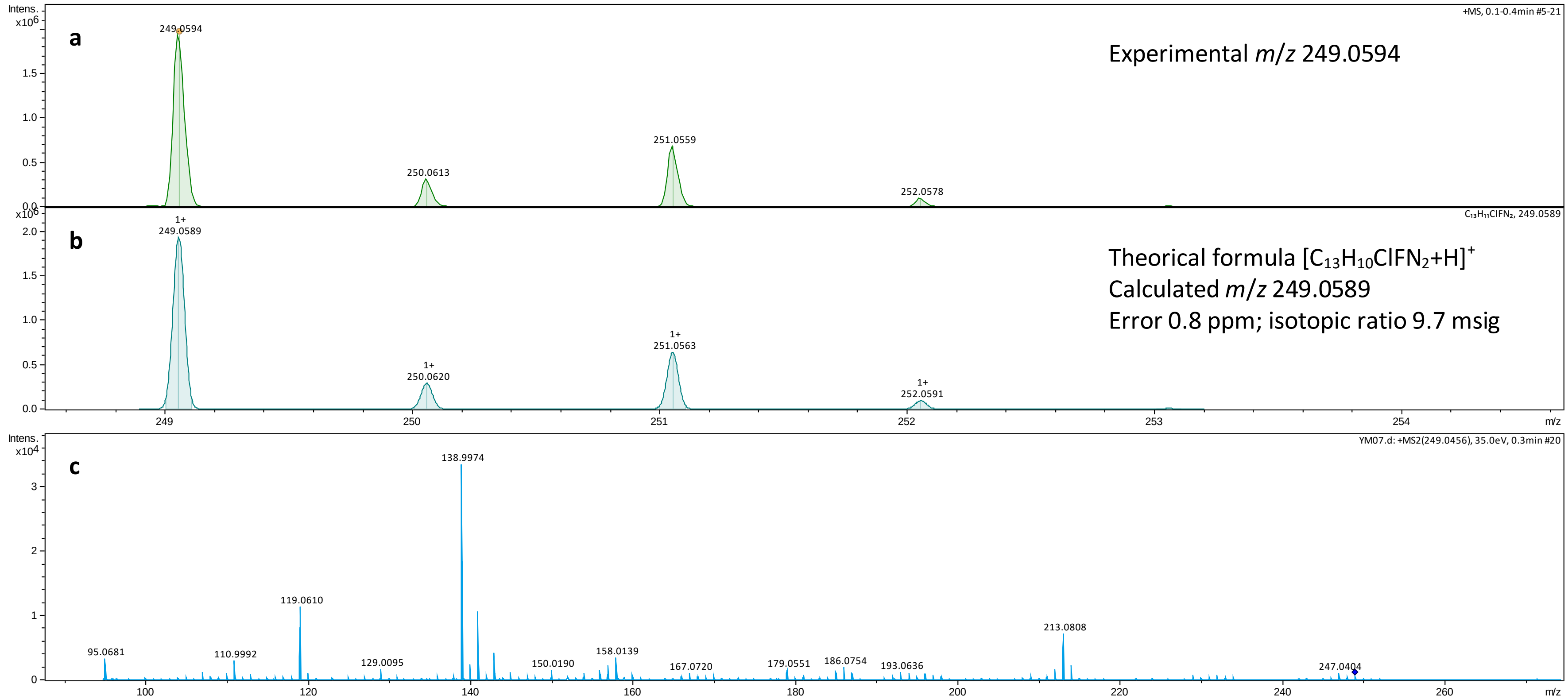

Figure S52. The HRMS analysis of compound **1f**. The experimental spectrum (**a**) and the simulated spectrum (**b**), both expanded between 248.5 and 254.6 Da highlighting the exact mass and isotopic ratio; the analysis in MS–MS mode (**c**) (fragmentation pathway).

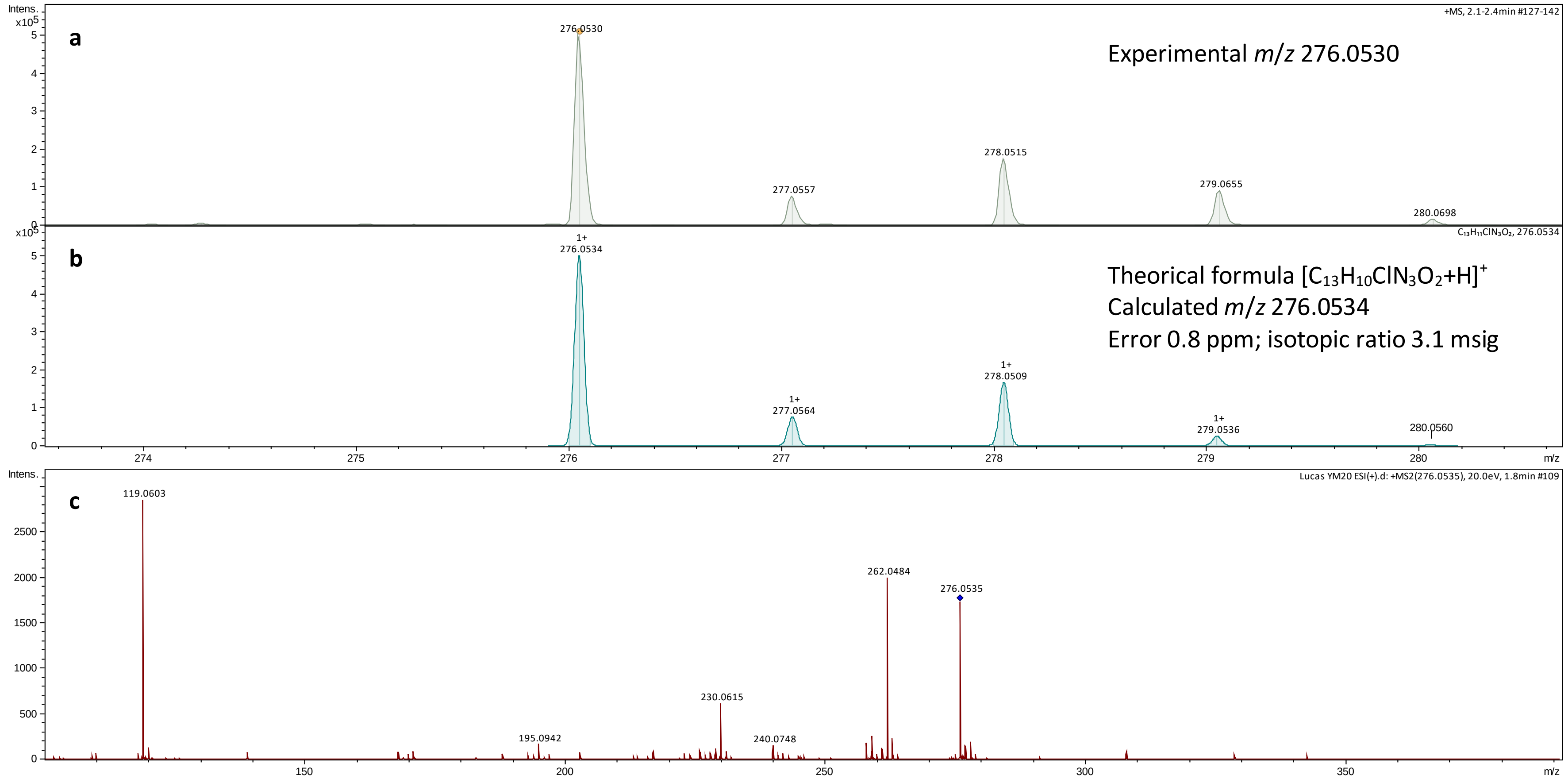

Figure S53. The HRMS analysis of compound **1g**. The experimental spectrum (**a**) and the simulated spectrum (**b**), both expanded between 248.5 and 254.6 Da highlighting the exact mass and isotopic ratio; the analysis in MS–MS mode (**c**) (fragmentation pathway).

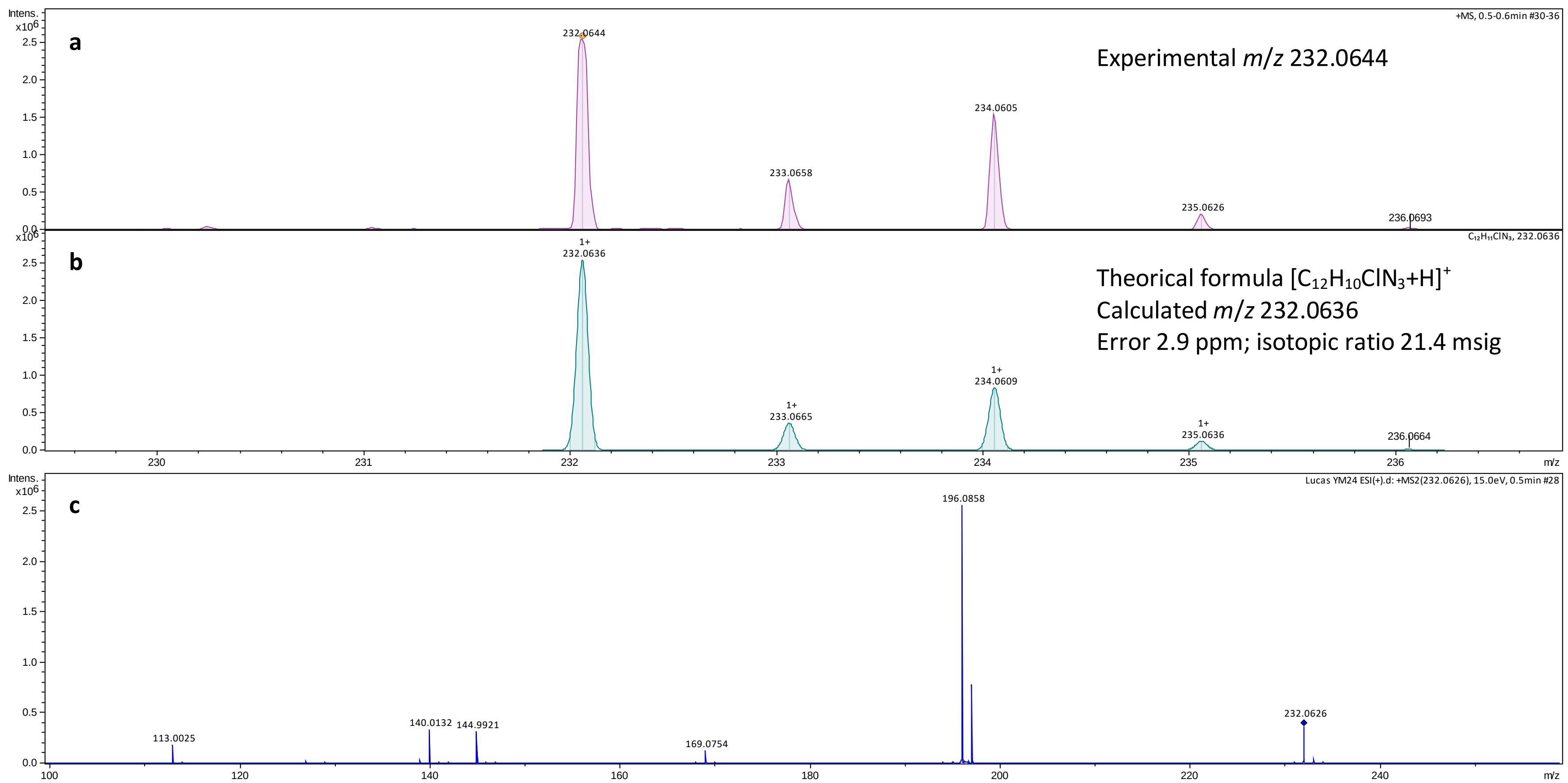

Figure S54. The HRMS analysis of compound **1h**. The experimental spectrum (**a**) and the simulated spectrum (**b**), both expanded between 229.5 and 236.8 Da highlighting the exact mass and isotopic ratio; the analysis in MS–MS mode (**c**) (fragmentation pathway).

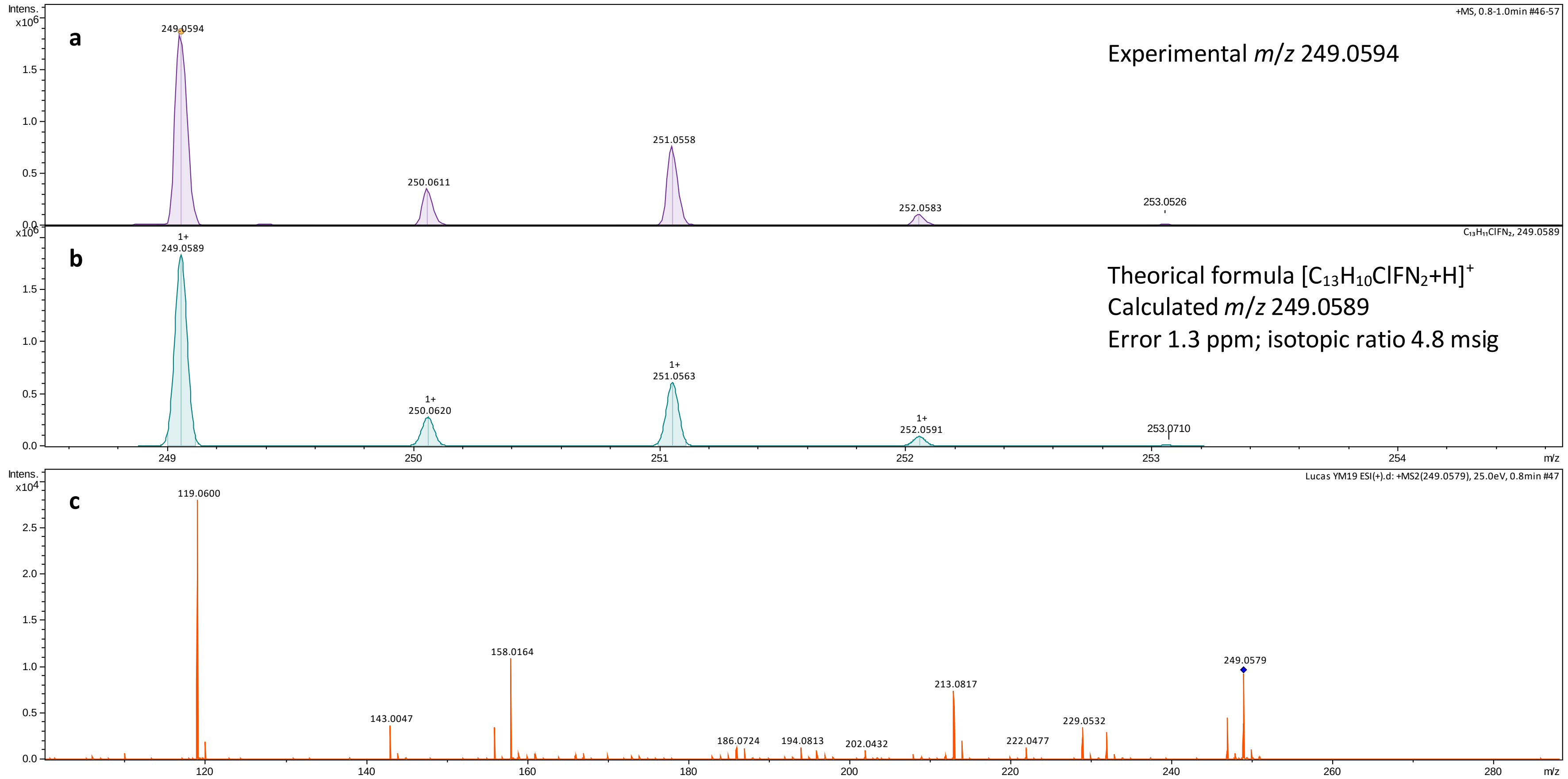

Figure S55. The HRMS analysis of compound **1i**. The experimental spectrum (**a**) and the simulated spectrum (**b**), both expanded between 248.5 and 254.6 Da highlighting the exact mass and isotopic ratio; the analysis in MS–MS mode (**c**) (fragmentation pathway).

HRMS spectra of *N*-phenyl-1*H*-indazoles

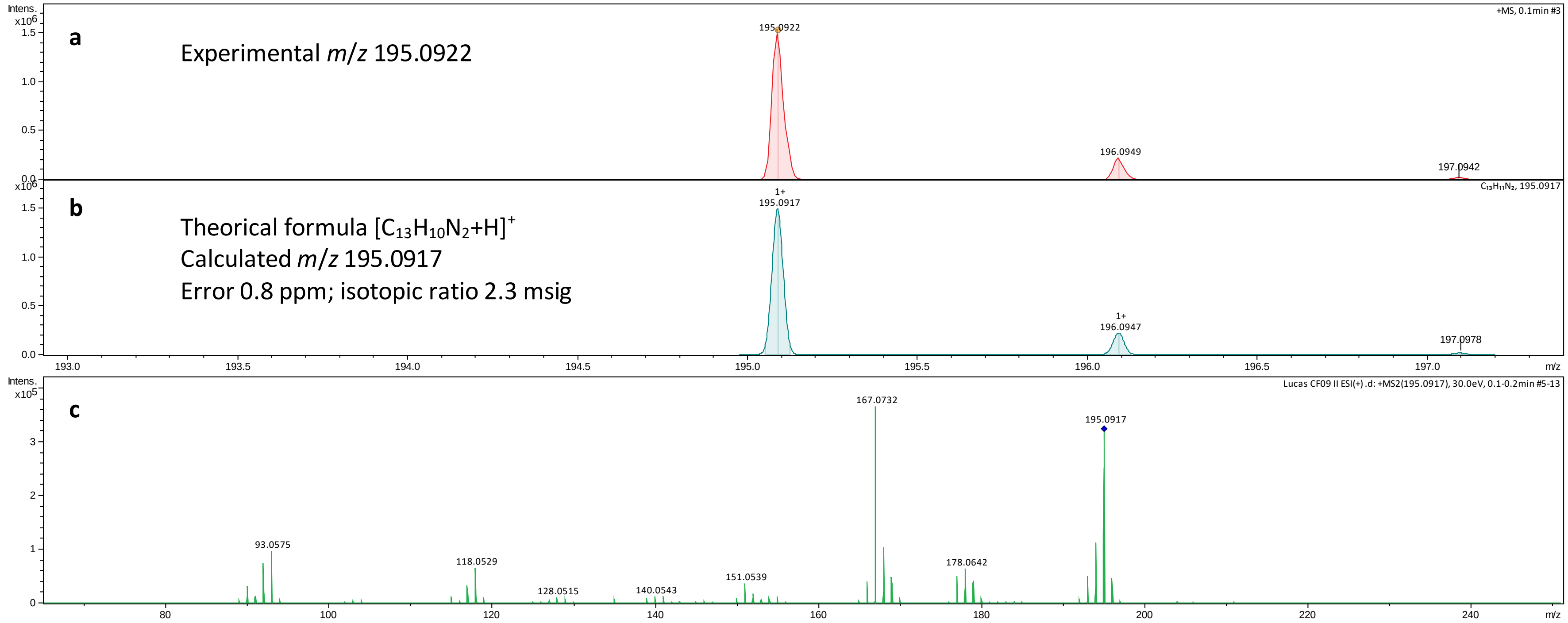

Figure S56. The HRMS analysis of compound **2a**. The experimental spectrum (**a**) and the simulated spectrum (**b**), both expanded between 192.9 and 197.4 Da highlighting the exact mass and isotopic ratio; the analysis in MS–MS mode (**c**) (fragmentation pathway).

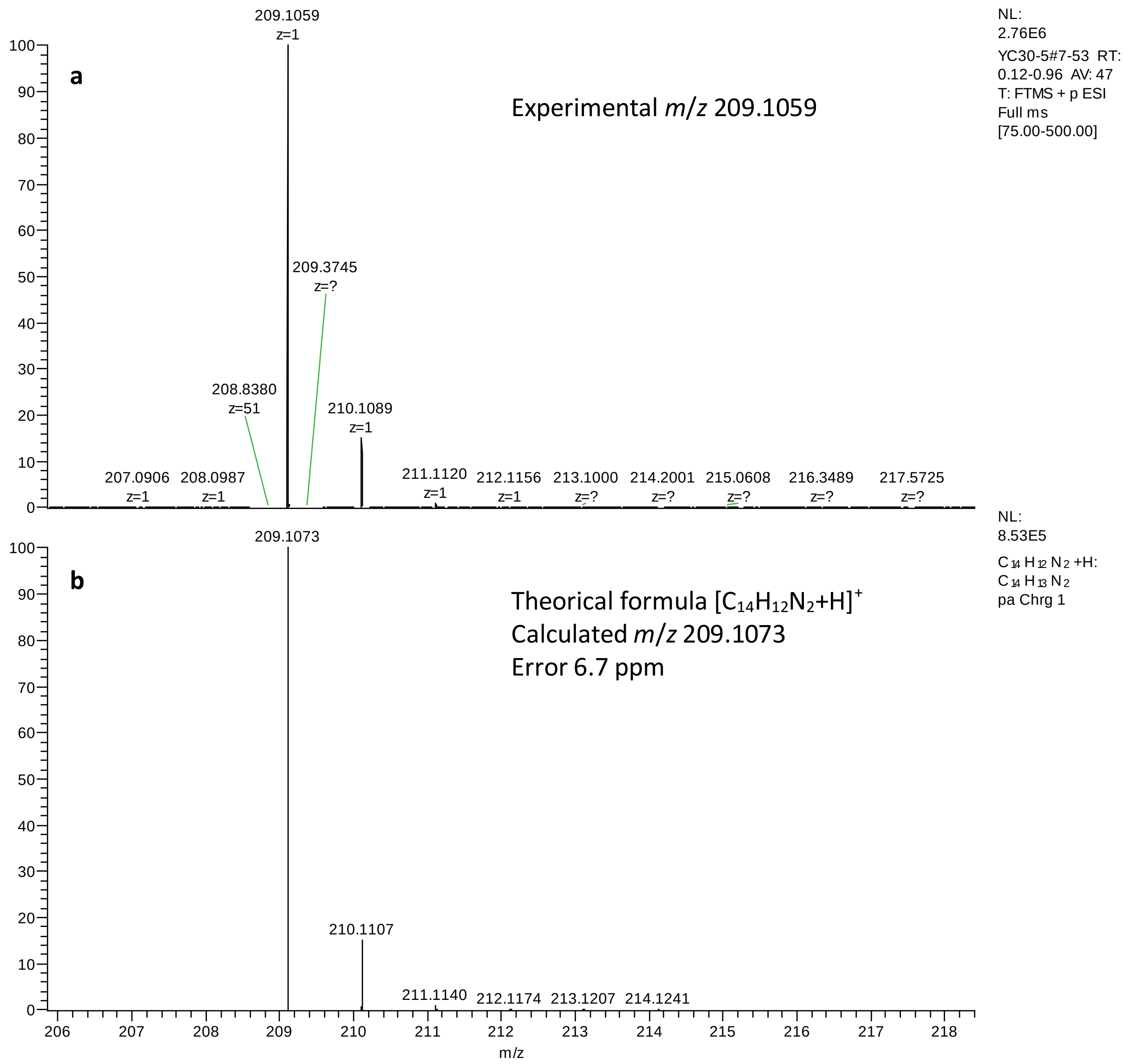

Figure S57. The HRMS analysis of compound **2b**. The experimental spectrum (**a**) and the simulated spectrum (**b**), both expanded between 205.9 and 218.4 Da highlighting the exact mass and isotopic ratio.

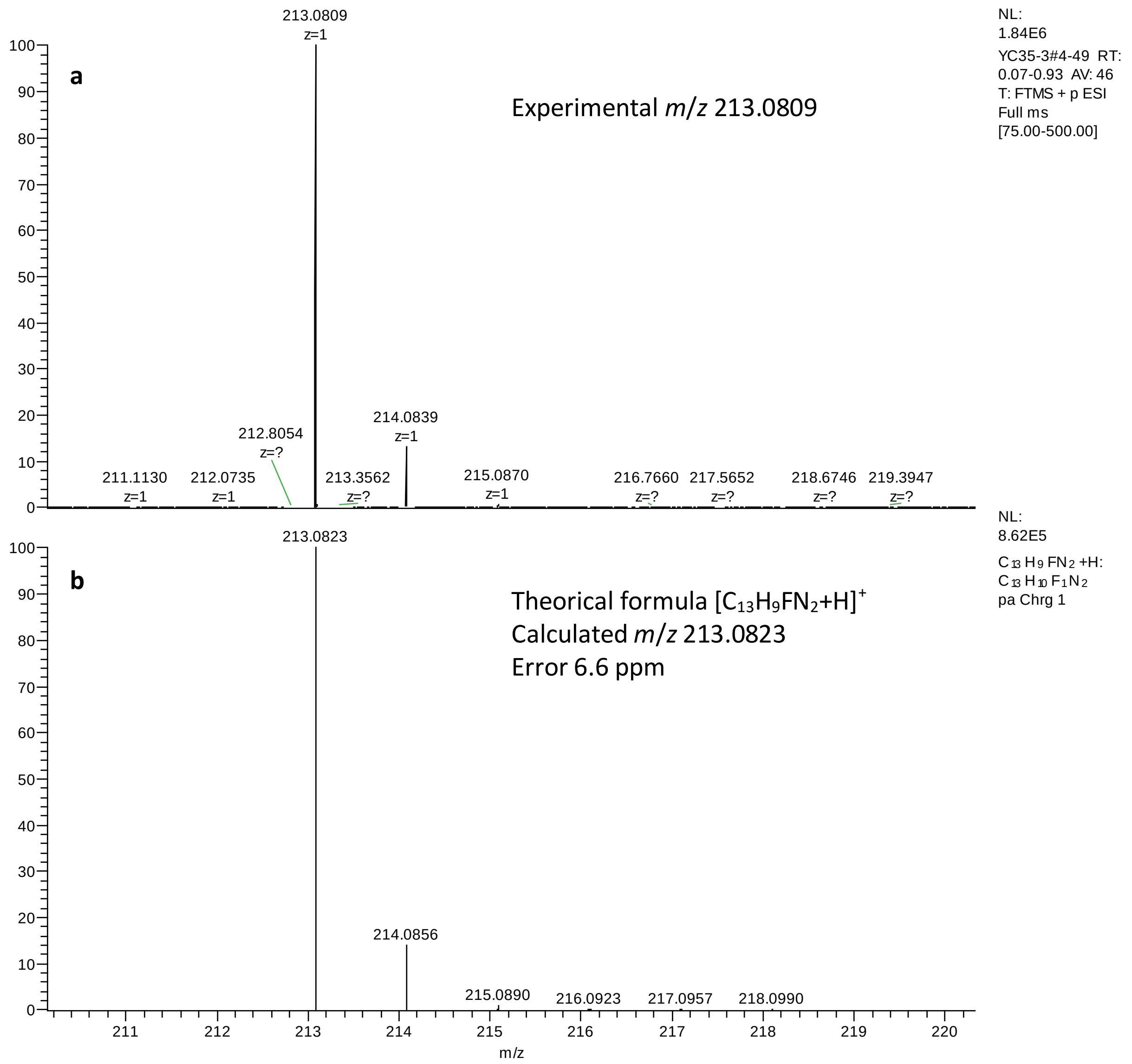

Figure S58. The HRMS analysis of compound **2d**. The experimental spectrum (**a**) and the simulated spectrum (**b**), both expanded between 210.1 and 220.3 Da highlighting the exact mass and isotopic ratio.

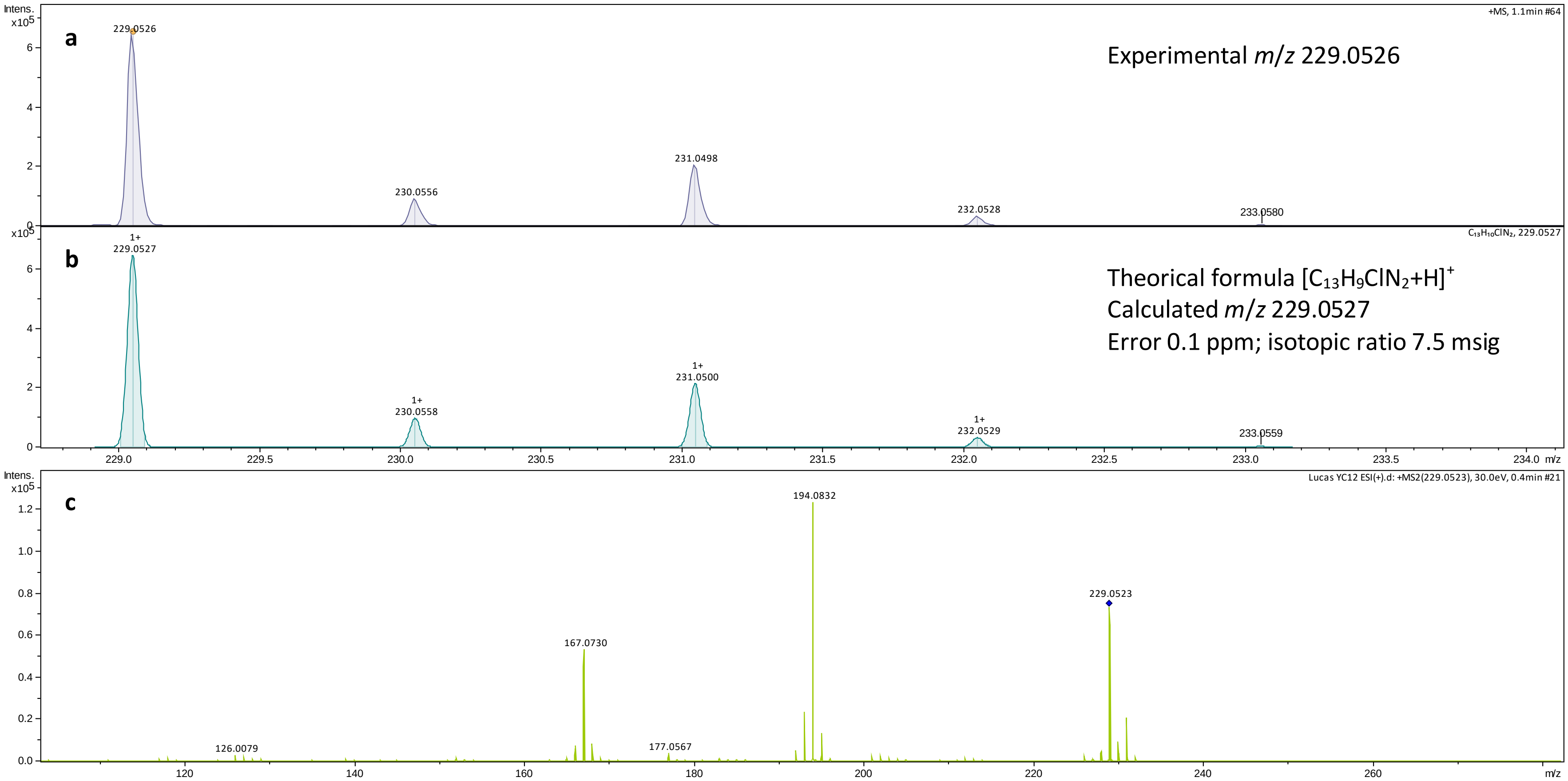

Figure S59. The HRMS analysis of compound **2e**. The experimental spectrum (**a**) and the simulated spectrum (**b**), both expanded between 228.7 and 234.1 Da highlighting the exact mass and isotopic ratio; the analysis in MS–MS mode (**c**) (fragmentation pathway).

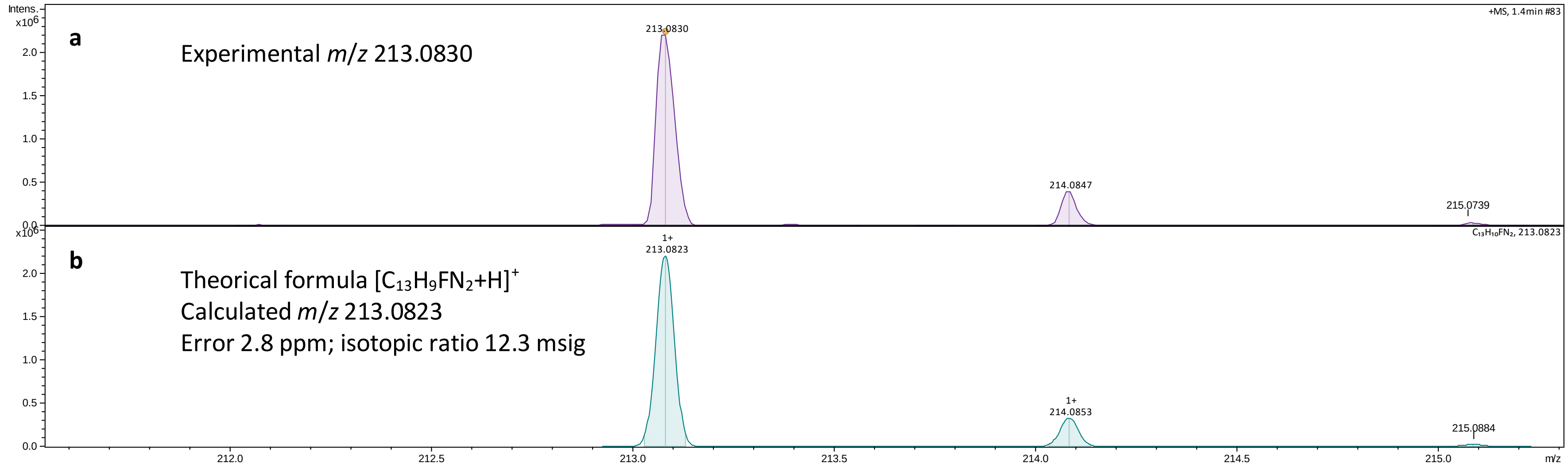

Figure S60. The HRMS analysis of compound **2f**. The experimental spectrum (**a**) and the simulated spectrum (**b**), both expanded between 211.1 and 215.6 Da highlighting the exact mass and isotopic ratio.

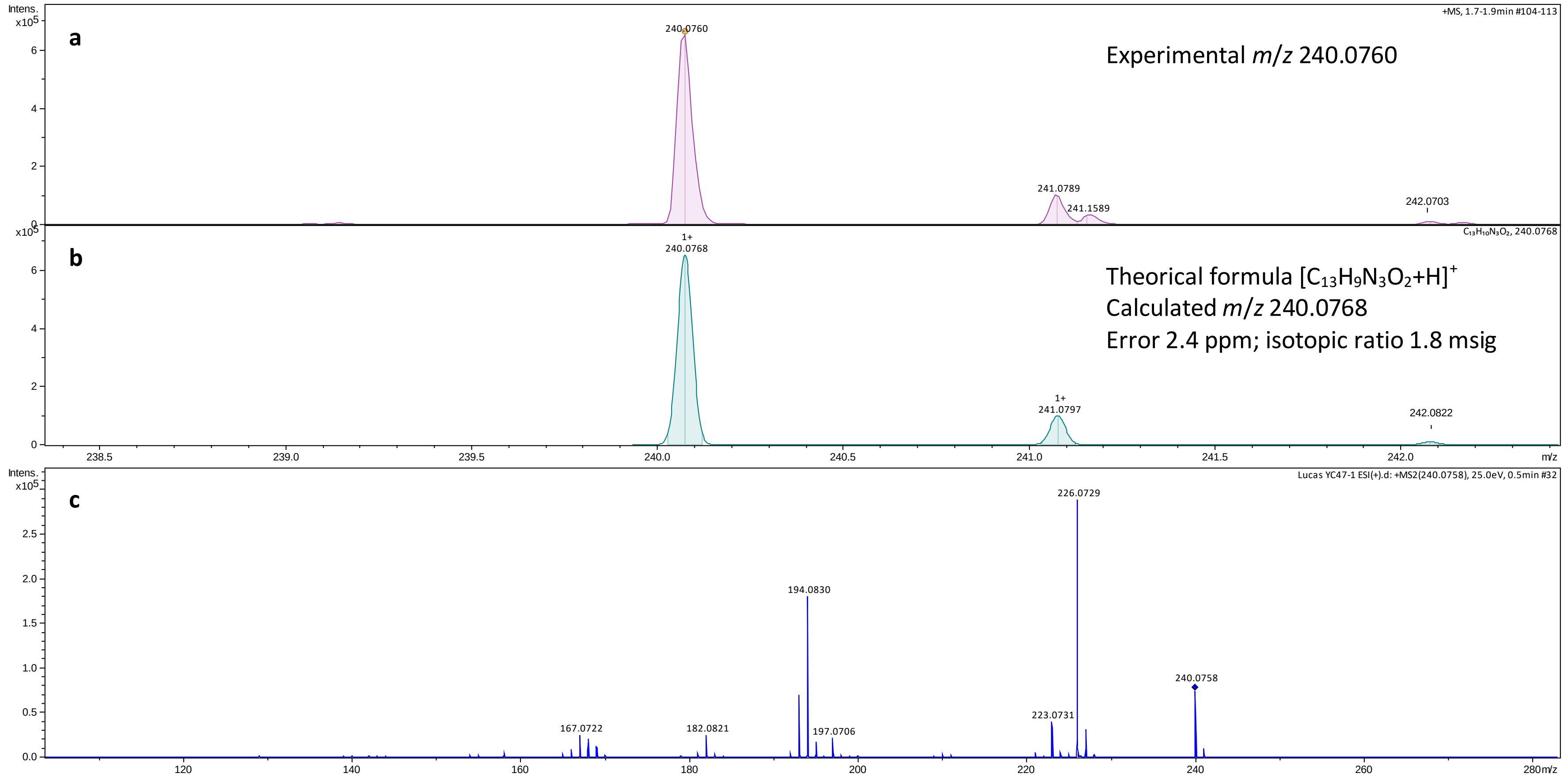

Figure S61. The HRMS analysis of compound **2g**. The experimental spectrum (**a**) and the simulated spectrum (**b**), both expanded between 238.4 and 242.4 Da highlighting the exact mass and isotopic ratio; the analysis in MS–MS mode (**c**) (fragmentation pathway).

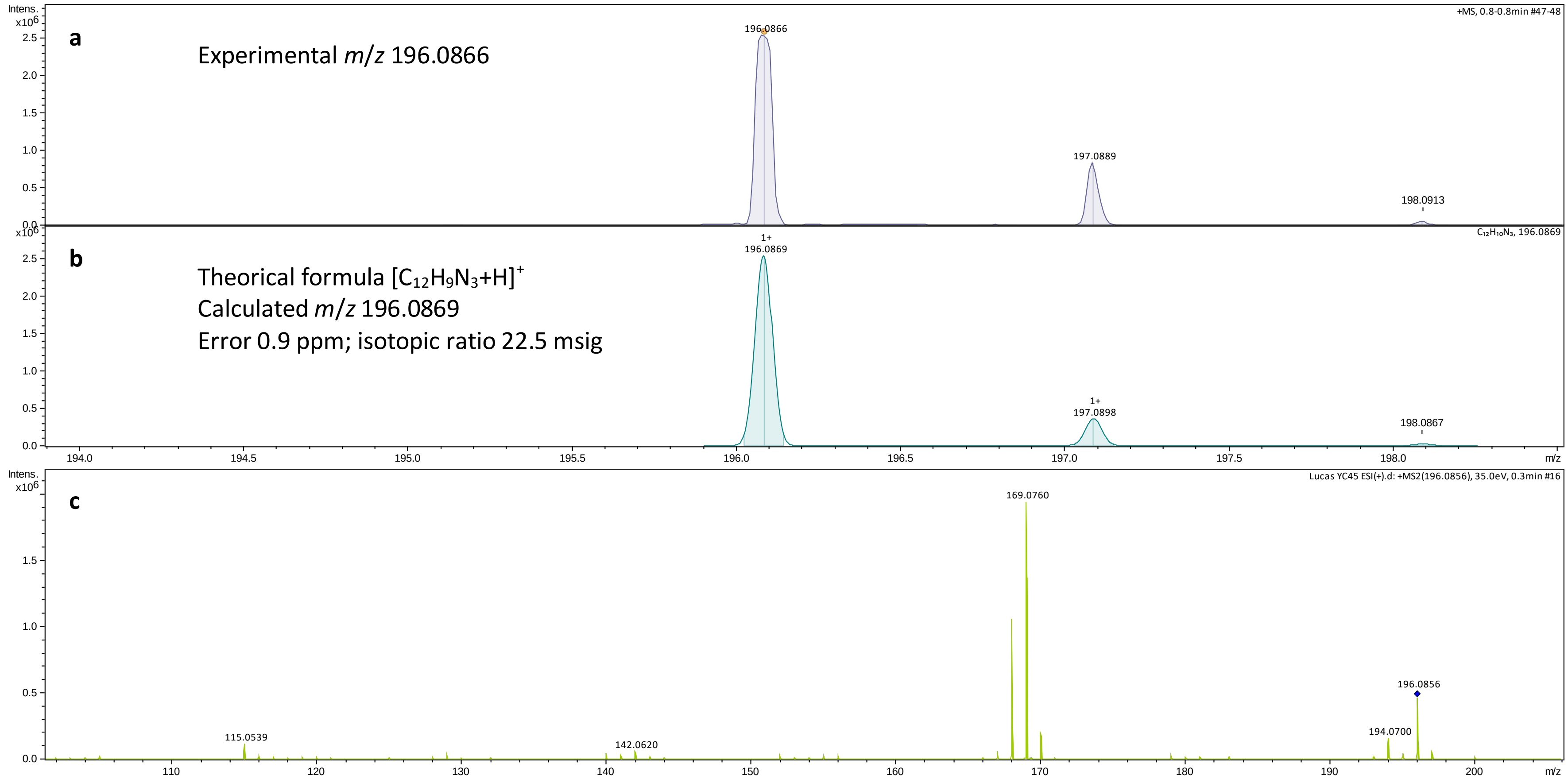

Figure S62. The HRMS analysis of compound **2h**. The experimental spectrum (**a**) and the simulated spectrum (**b**), both expanded between 193.9 and 198.5 Da highlighting the exact mass and isotopic ratio; the analysis in MS–MS mode (**c**) (fragmentation pathway).

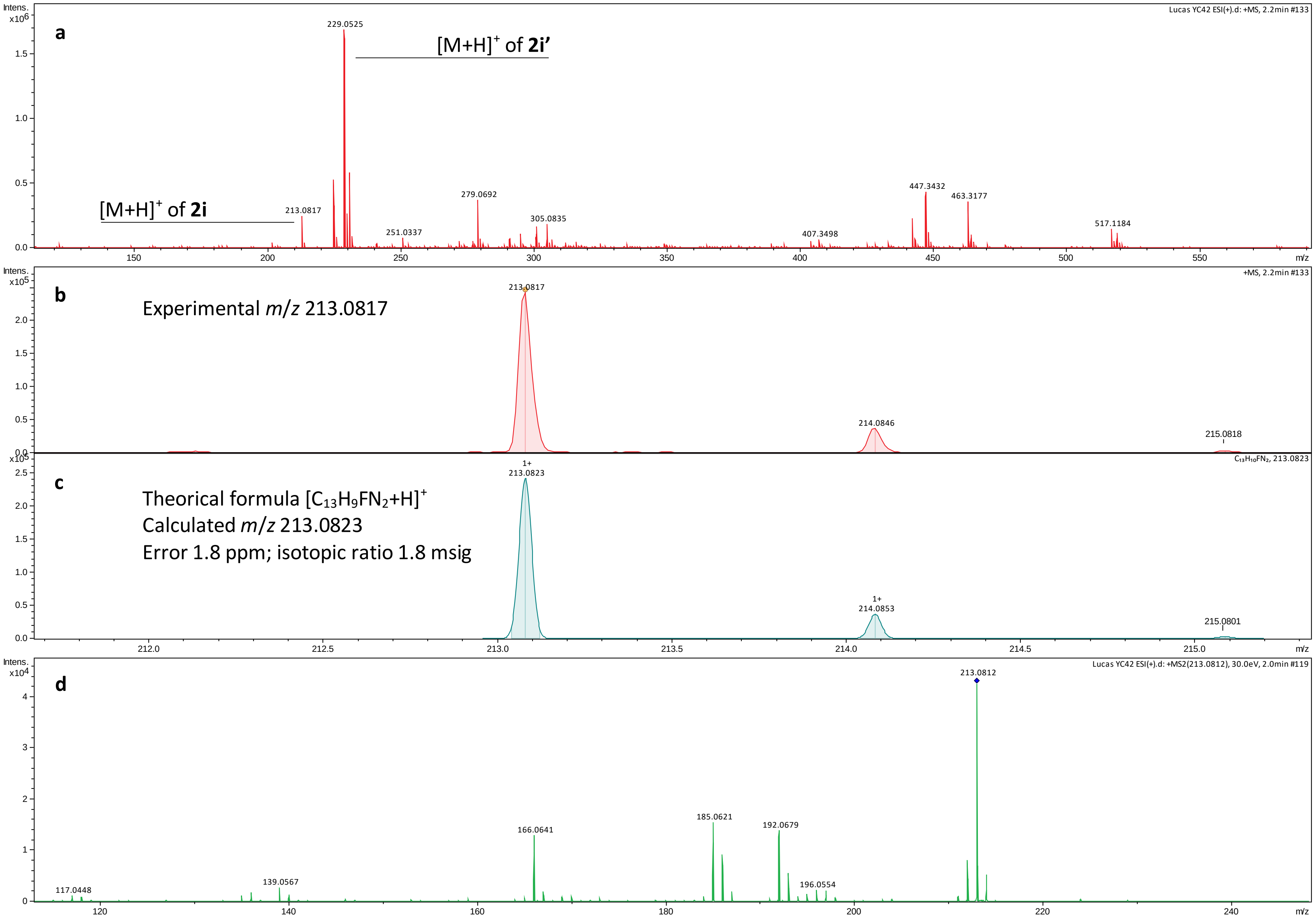

Figure S63. The HRMS analysis of the mixture of compounds **2i+2i'**. The full experimental spectrum (**a**); the experimental spectrum (**b**) and the simulated spectrum (**c**), both expanded between 211.4 and 215.6 Da highlighting the exact mass and isotopic ratio; the analysis in MS–MS mode (**d**) (fragmentation pathway).

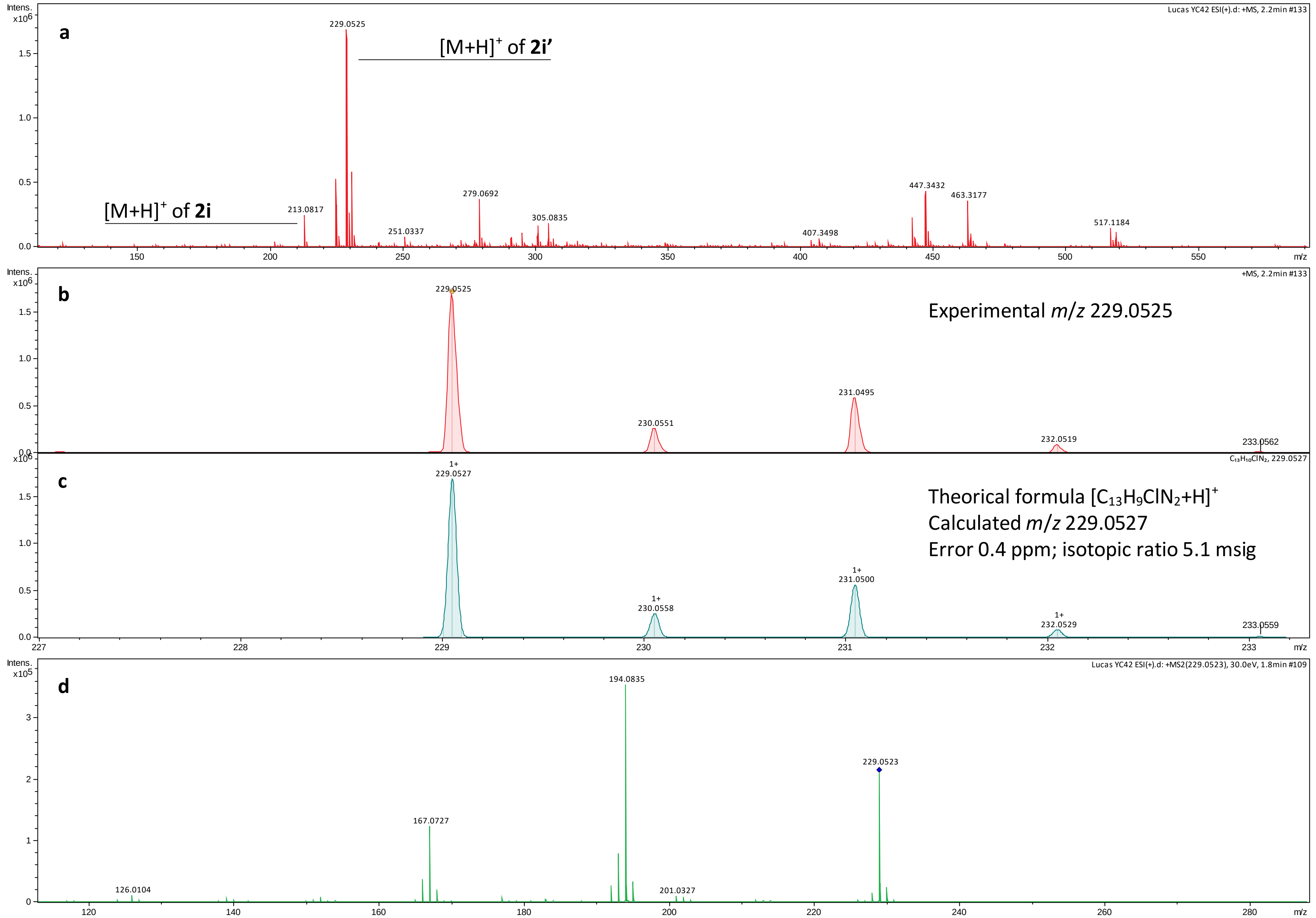

Figure S64. The HRMS analysis of the mixture of compounds **2i** and **2i'**. The full experimental spectrum (**a**); the experimental spectrum (**b**) and the simulated spectrum (**c**), both expanded between 227.0 and 233.3 Da highlighting the exact mass and isotopic ratio; the analysis in MS–MS mode (**d**) (fragmentation pathway).

HRMS spectra of *N*-thiazolylhydrazones 3a–i

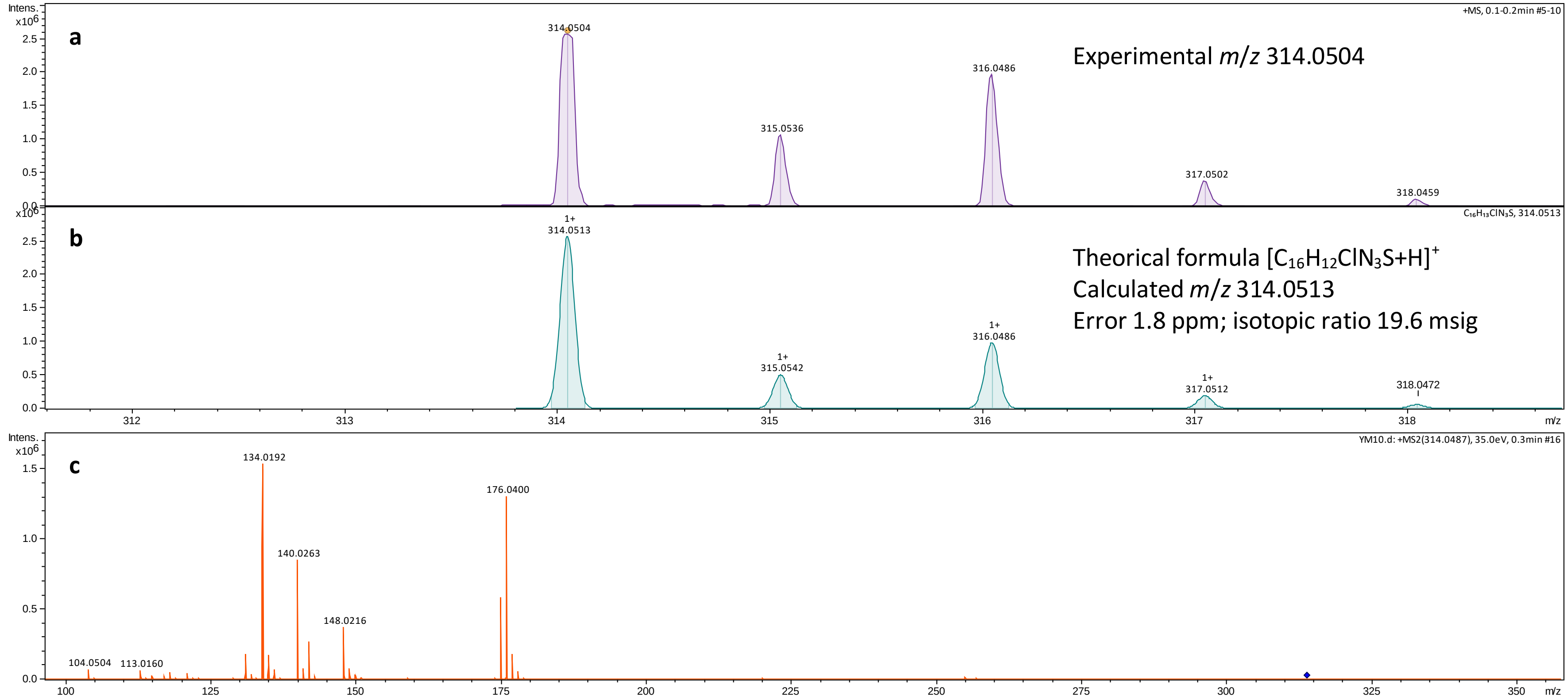

Figure S65. The HRMS analysis of compound **3a**. The experimental spectrum (**a**) and the simulated spectrum (**b**), both expanded between 311.8 and 318.7 Da highlighting the exact mass and isotopic ratio; the analysis in MS–MS mode (**c**) (fragmentation pathway).

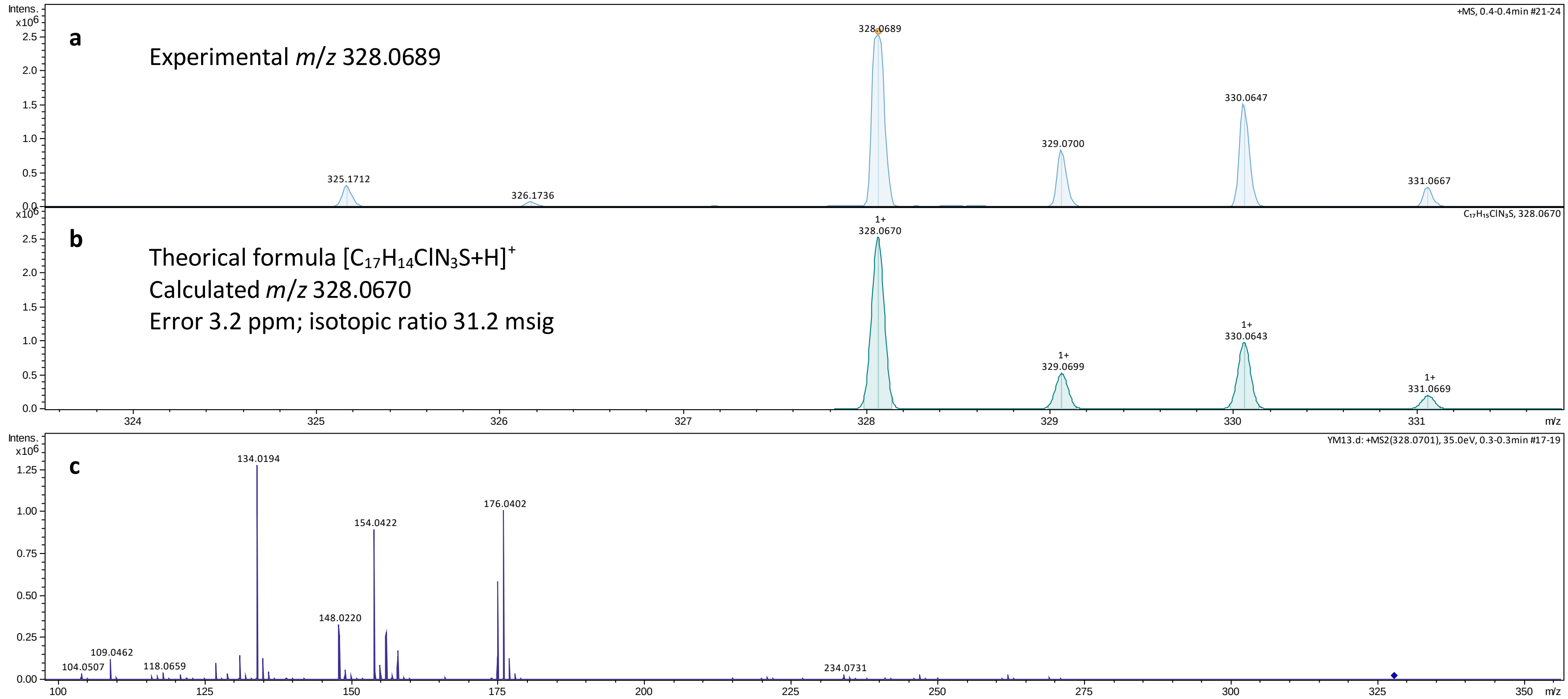

Figure S66. The HRMS analysis of compound **3b**. The experimental spectrum (**a**) and the simulated spectrum (**b**), both expanded between 323.5 and 331.8 Da highlighting the exact mass and isotopic ratio; the analysis in MS–MS mode (**c**) (fragmentation pathway).

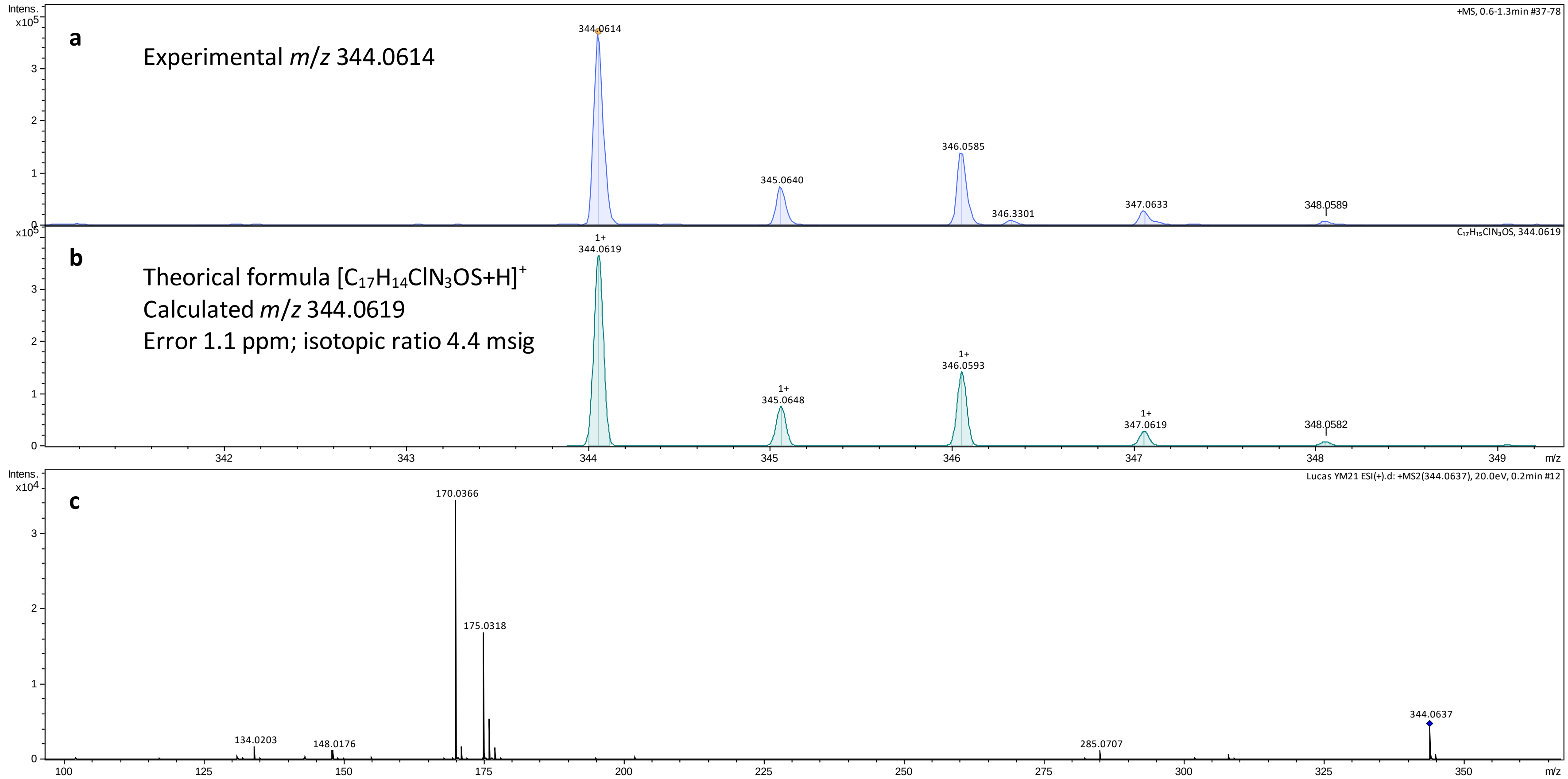

Figure S67. The HRMS analysis of compound **3c**. The experimental spectrum (**a**) and the simulated spectrum (**b**), both expanded between 341.0 and 349.2 Da highlighting the exact mass and isotopic ratio; the analysis in MS–MS mode (**c**) (fragmentation pathway).

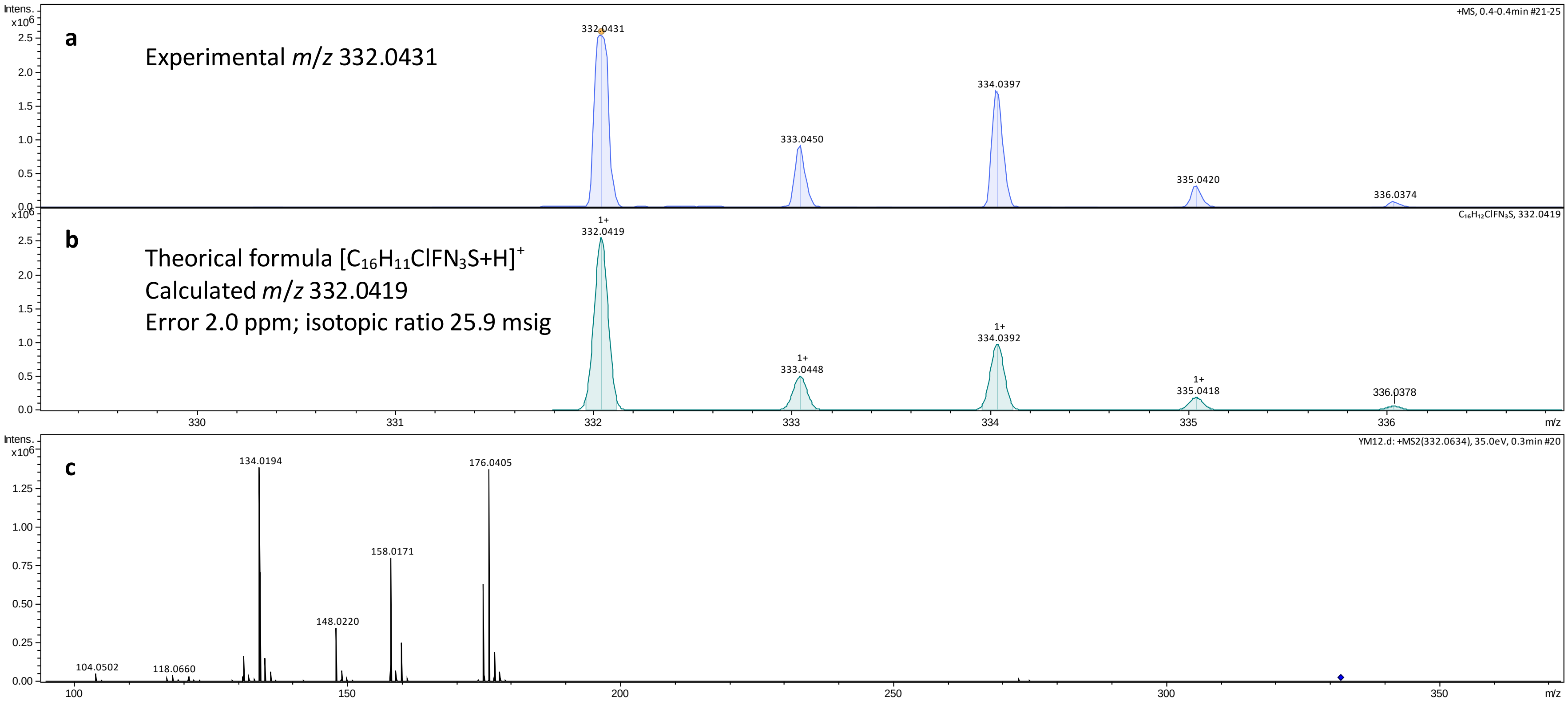

Figure S68. The HRMS analysis of compound **3d**. The experimental spectrum (**a**) and the simulated spectrum (**b**), both expanded between 329.2 and 336.9 Da highlighting the exact mass and isotopic ratio; the analysis in MS–MS mode (**c**) (fragmentation pathway).

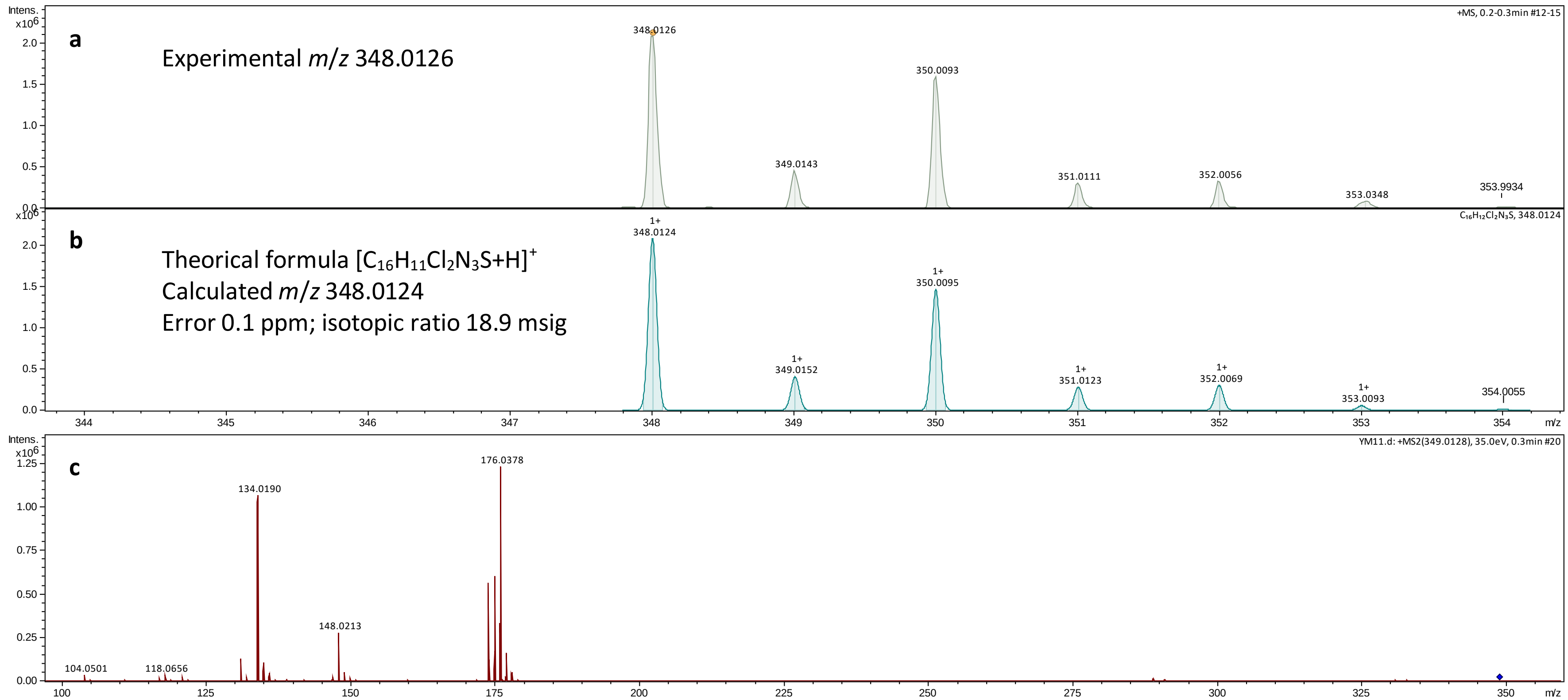

Figure S69. The HRMS analysis of compound **3e**. The experimental spectrum (**a**) and the simulated spectrum (**b**), both expanded between 343.7 and 354.4 Da highlighting the exact mass and isotopic ratio; the analysis in MS–MS mode (**c**) (fragmentation pathway).

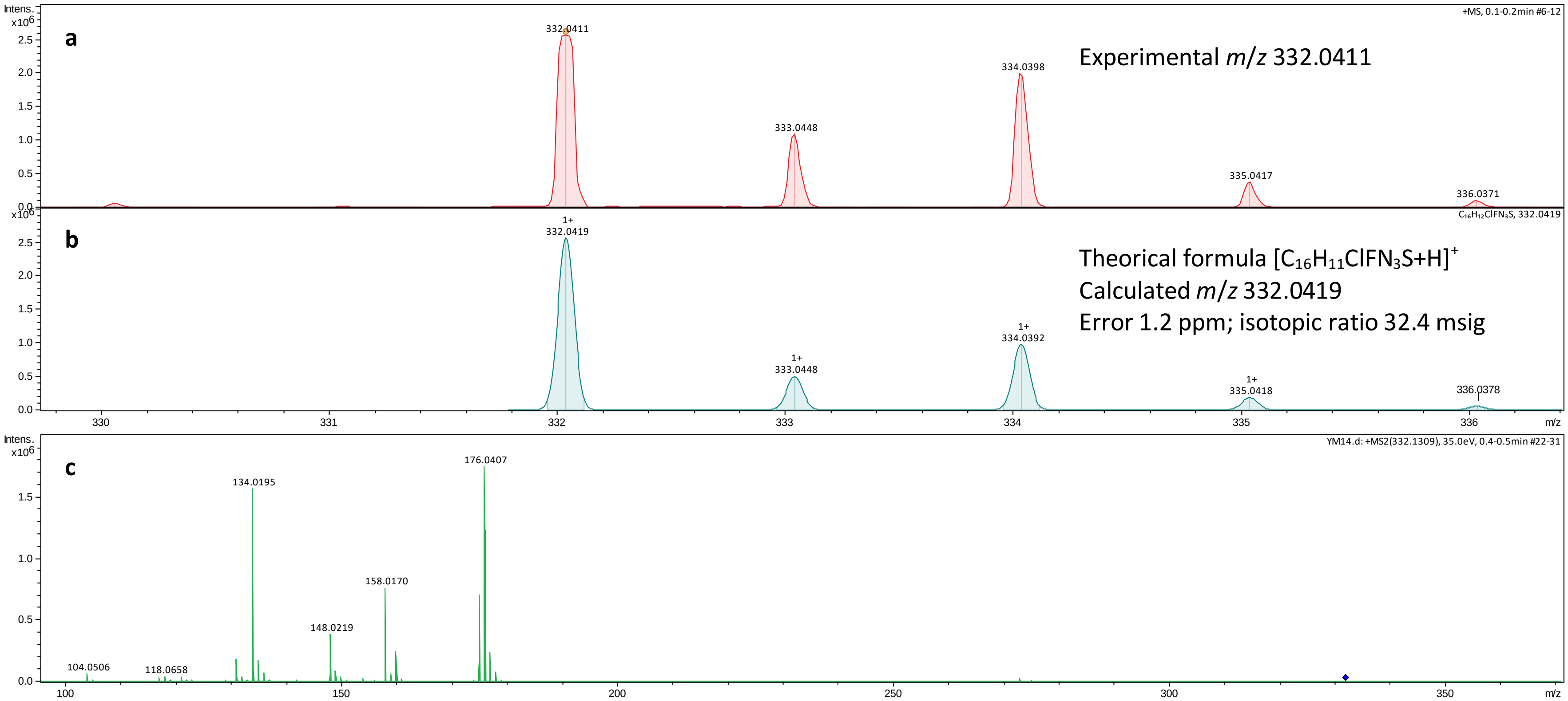

Figure S70. The HRMS analysis of compound **3f**. The experimental spectrum (**a**) and the simulated spectrum (**b**), both expanded between 329.2 and 336.9 Da highlighting the exact mass and isotopic ratio; the analysis in MS–MS mode (**c**) (fragmentation pathway).

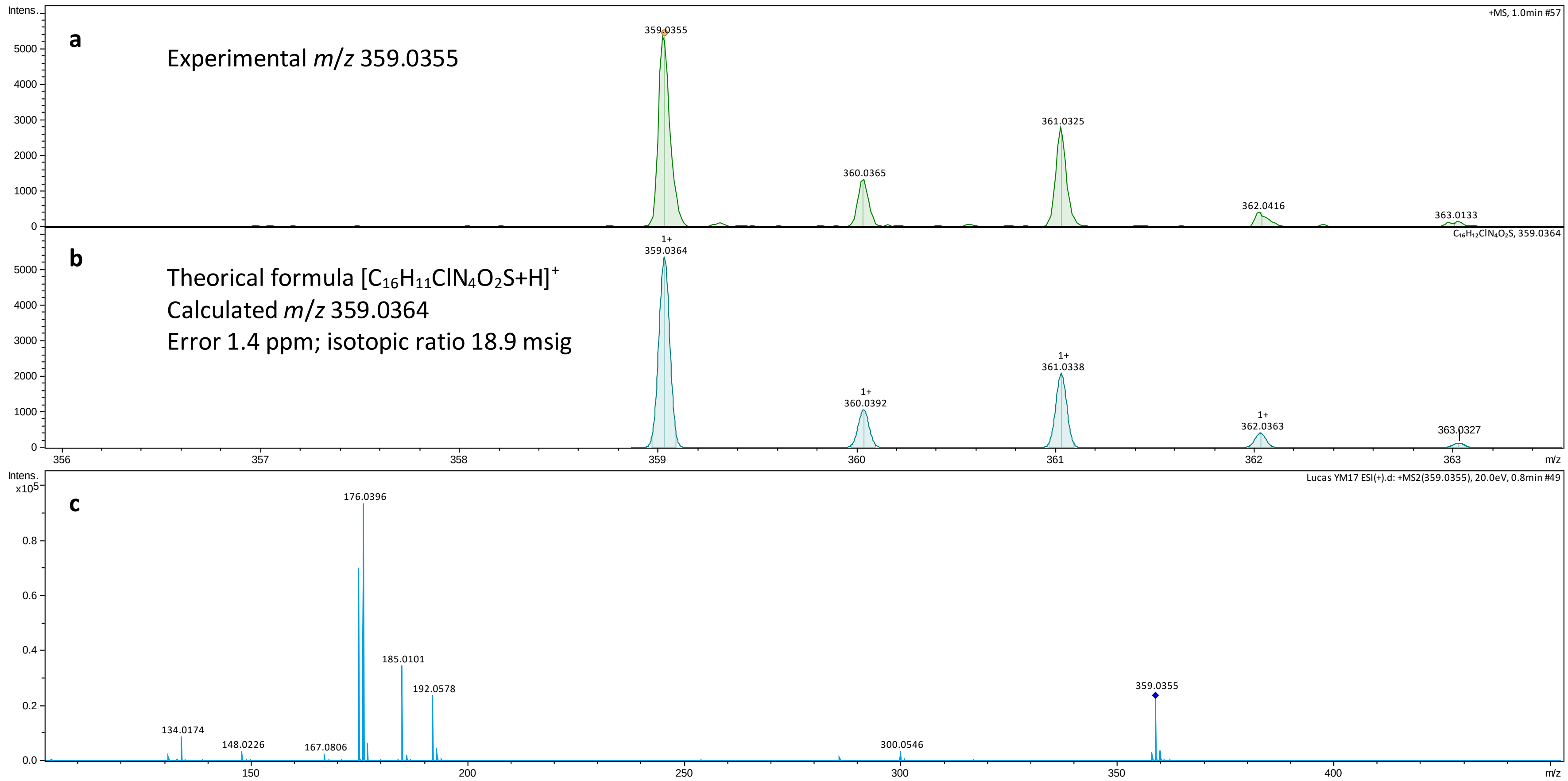

Figure S71. The HRMS analysis of compound **3g**. The experimental spectrum (**a**) and the simulated spectrum (**b**), both expanded between 356.0 and 363.6 Da highlighting the exact mass and isotopic ratio; the analysis in MS–MS mode (**c**) (fragmentation pathway).

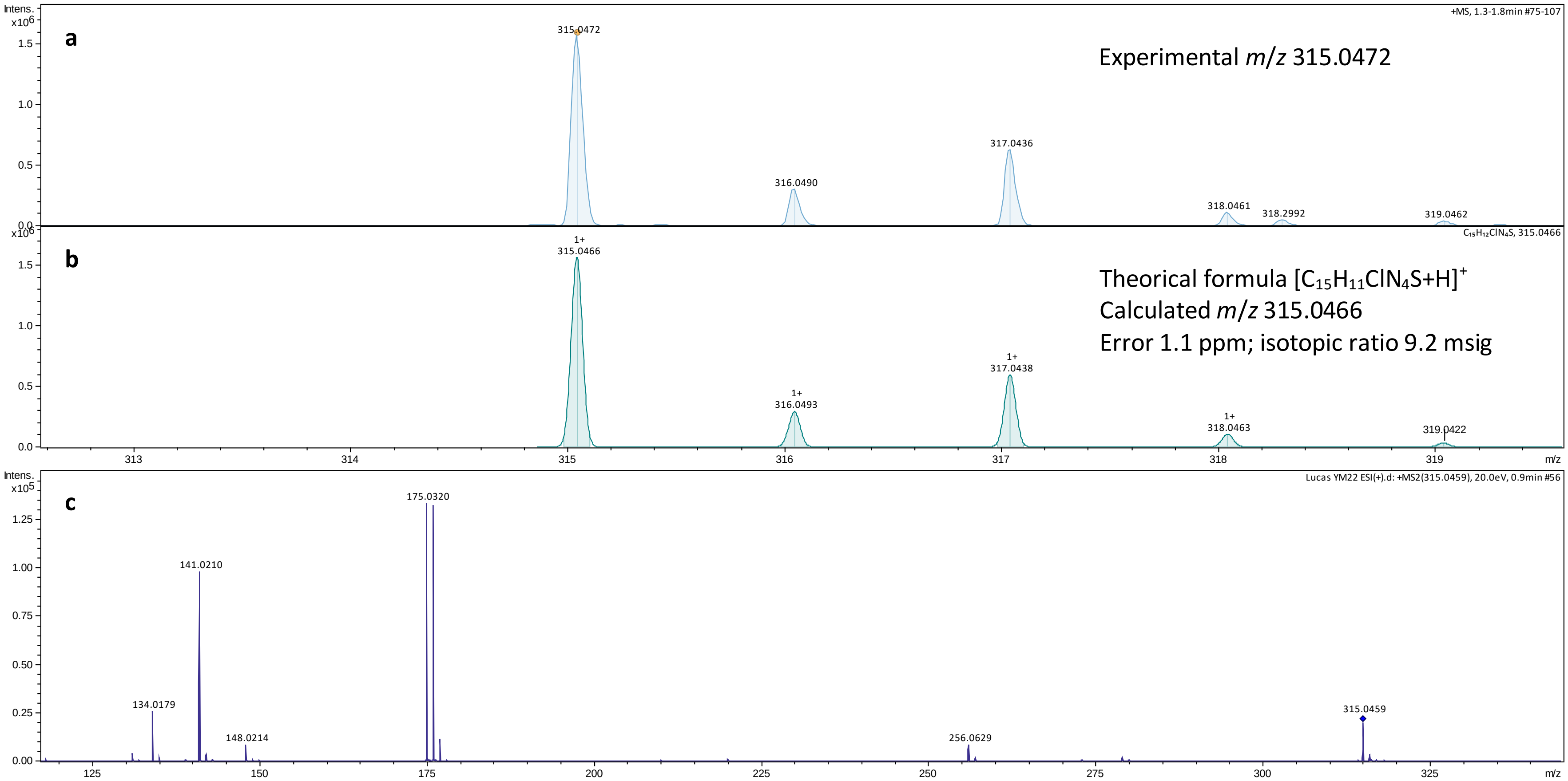

Figure S72. The HRMS analysis of compound **3h**. The experimental spectrum (**a**) and the simulated spectrum (**b**), both expanded between 312.6 and 319.6 Da highlighting the exact mass and isotopic ratio; the analysis in MS–MS mode (**c**) (fragmentation pathway).

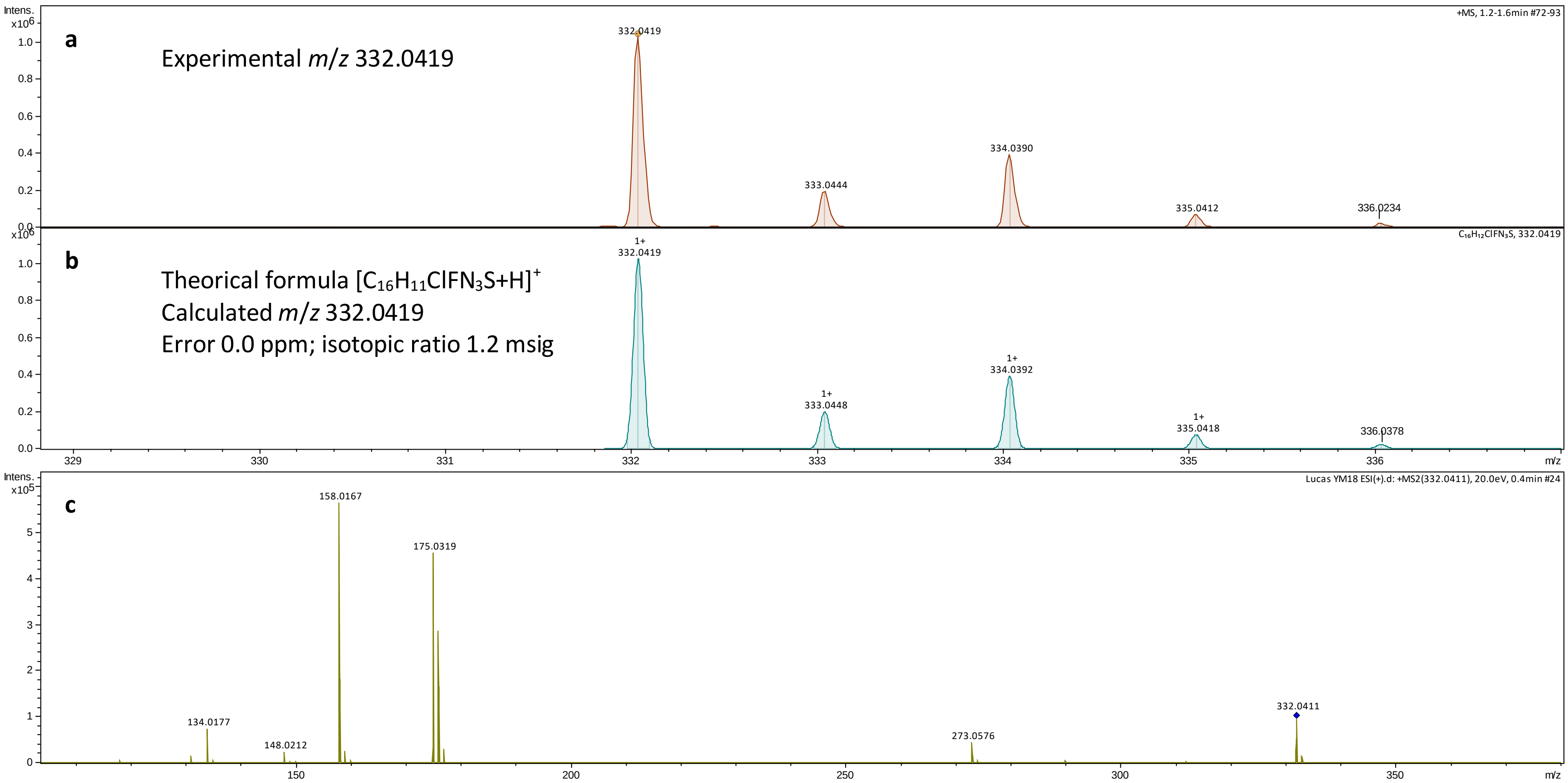

Figure S73. The HRMS analysis of compound **3i**. The experimental spectrum (**a**) and the simulated spectrum (**b**), both expanded between 328.8 and 337.0 Da highlighting the exact mass and isotopic ratio; the analysis in MS–MS mode (**c**) (fragmentation pathway).

HRMS spectra of *N*-thiazolyl-1*H*-indazoles

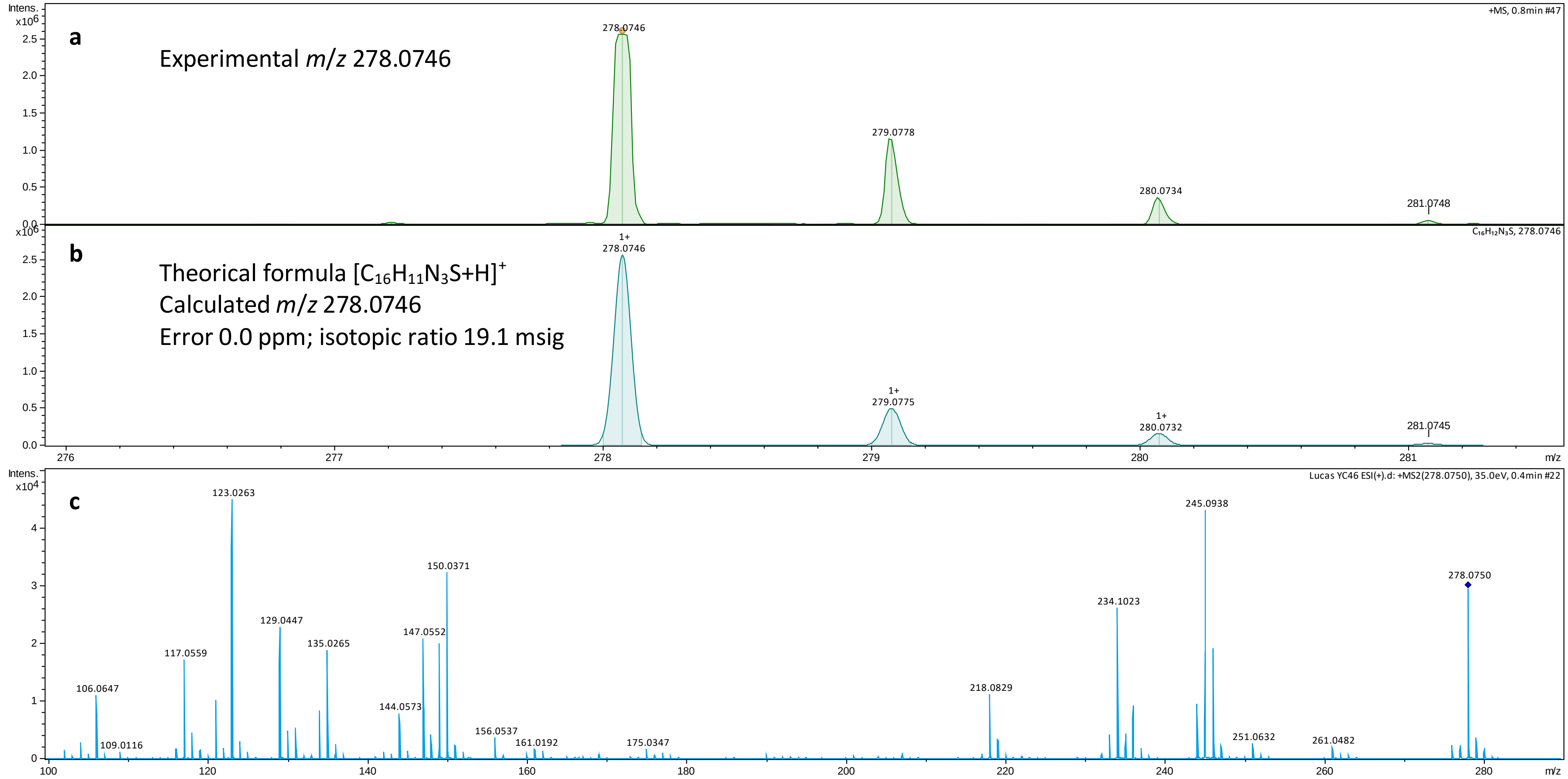

Figure S74. The HRMS analysis of compound **4a**. The experimental spectrum (**a**) and the simulated spectrum (**b**), both expanded between 275.9 and 281.6 Da highlighting the exact mass and isotopic ratio; the analysis in MS–MS mode (**c**) (fragmentation pathway).

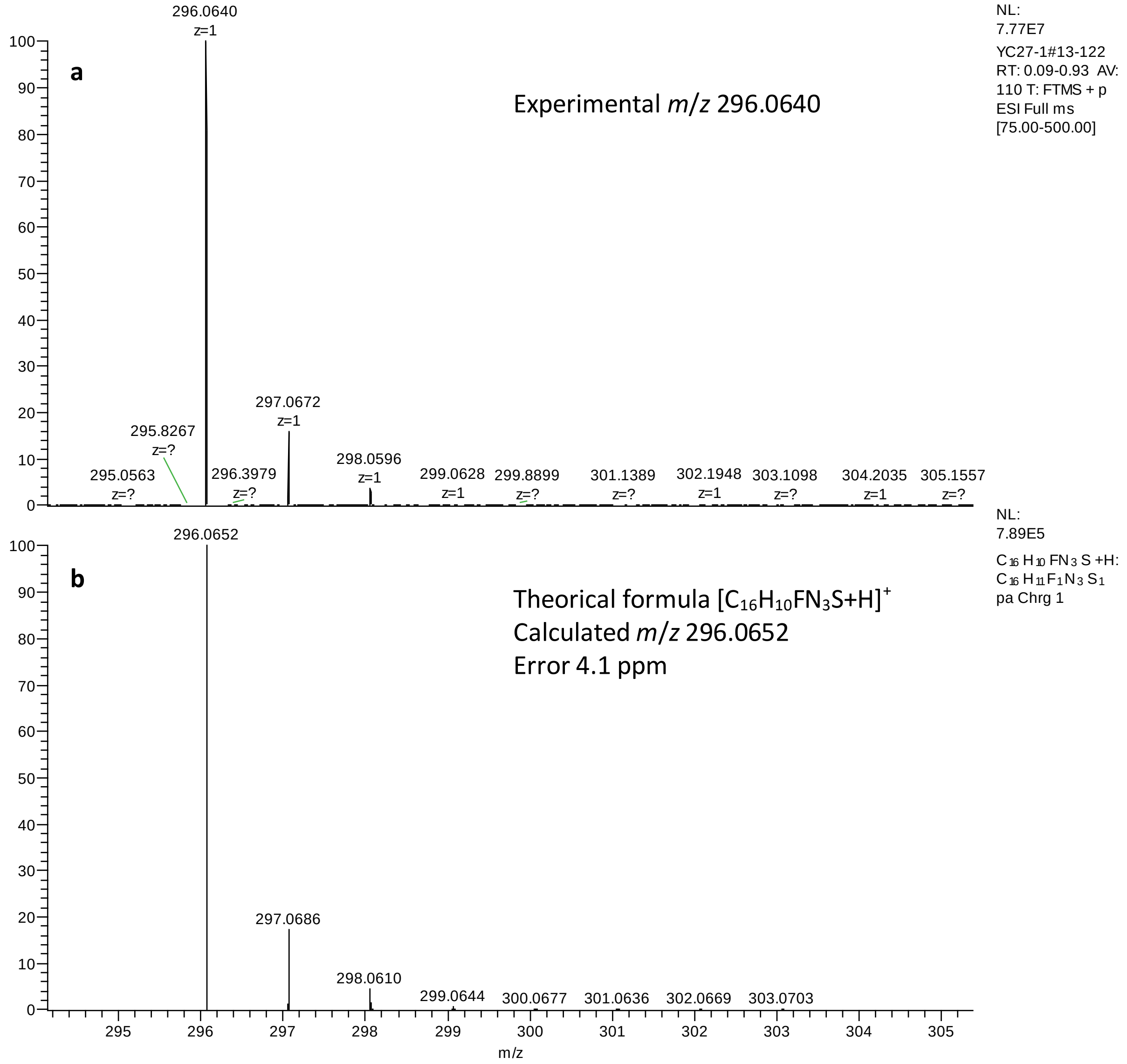

Figure S75. The HRMS analysis of compound **4d**. The experimental spectrum (**a**) and the simulated spectrum (**b**), both expanded between 294.2 and 305.4 Da highlighting the exact mass and isotopic ratio.

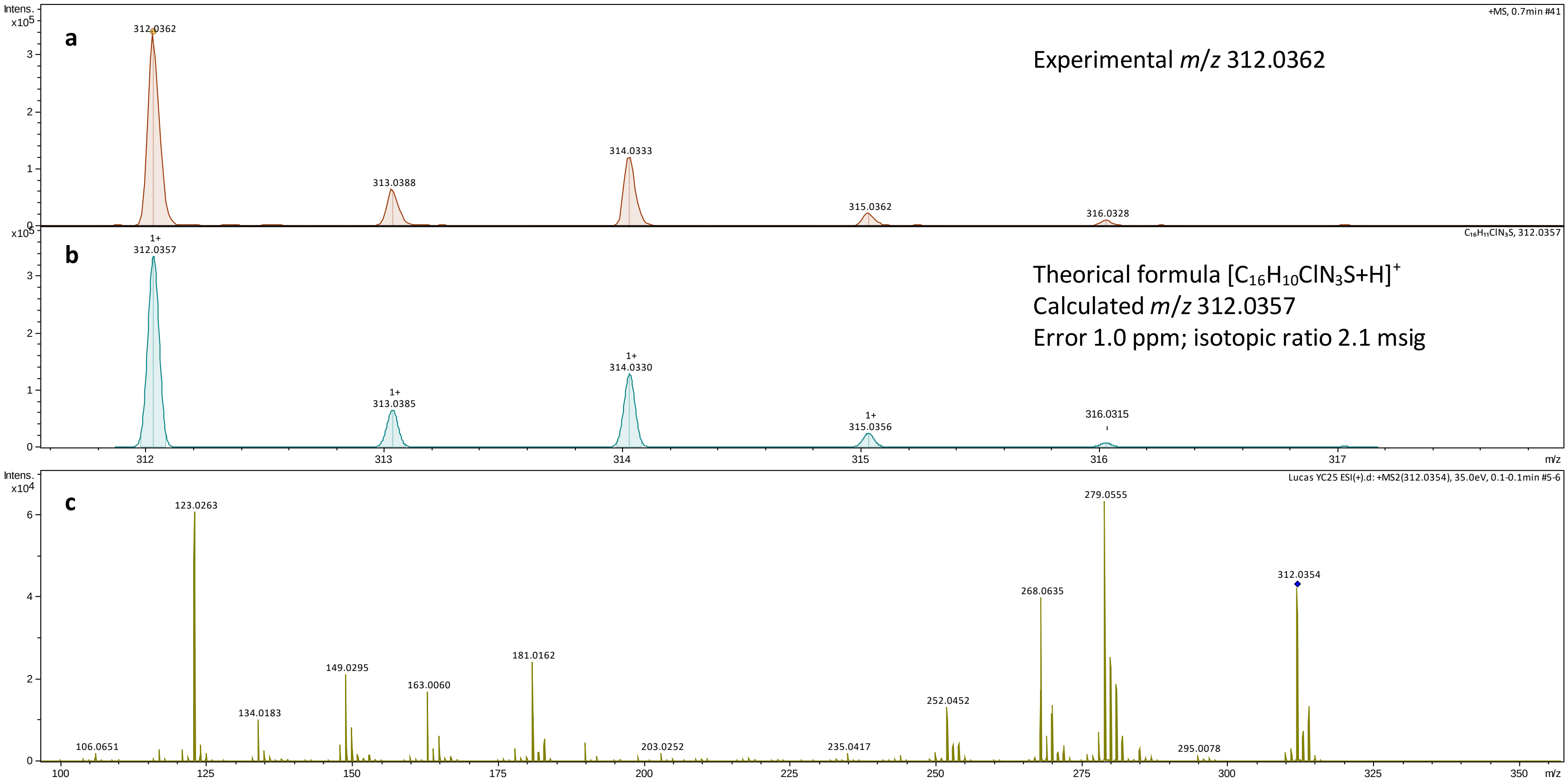

Figure S76. The HRMS analysis of compound **4e**. The experimental spectrum (**a**) and the simulated spectrum (**b**), both expanded between 311.8 and 317.9 Da highlighting the exact mass and isotopic ratio; the analysis in MS–MS mode (**c**) (fragmentation pathway).

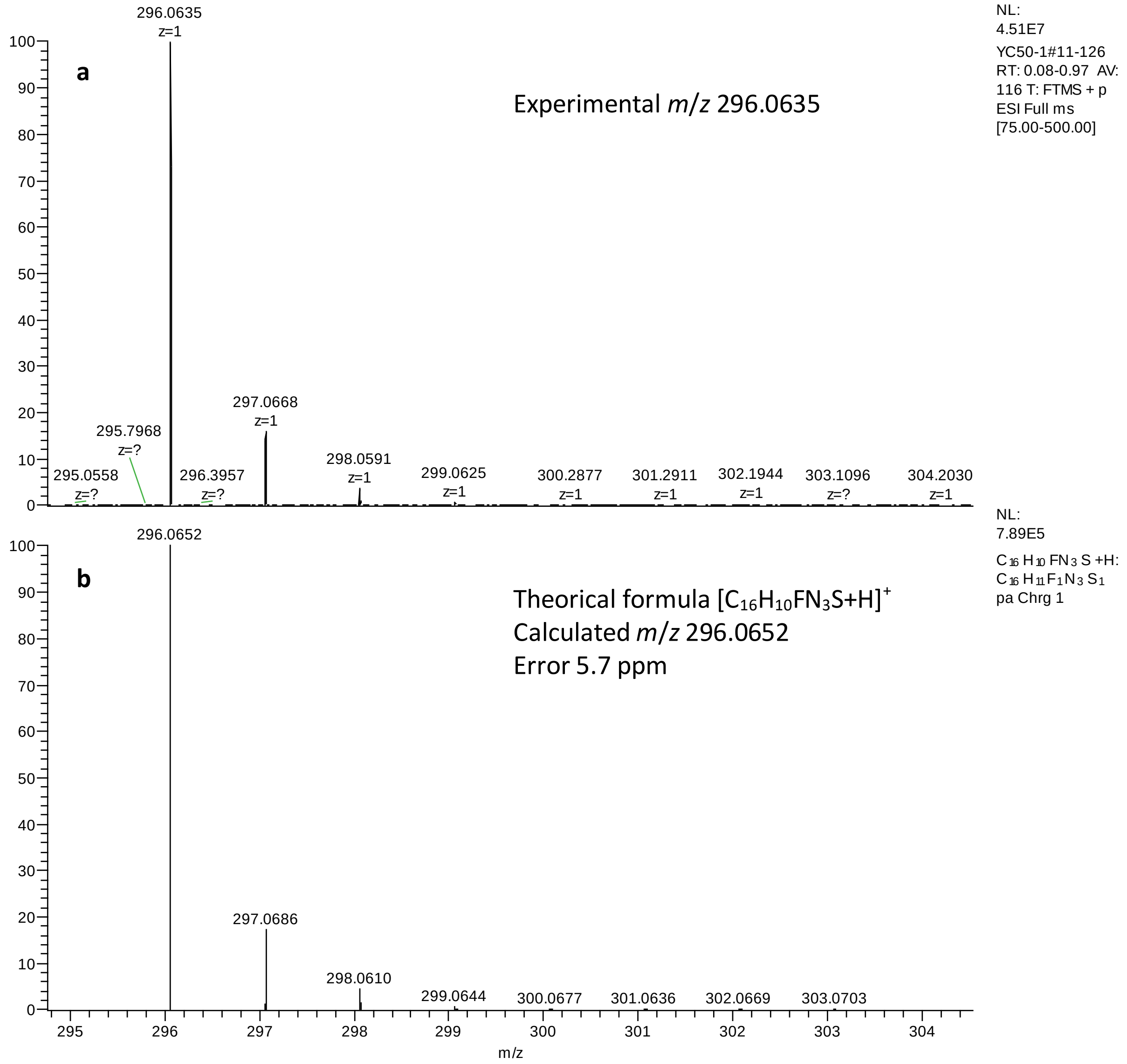

Figure S77. The HRMS analysis of compound **4f**. The experimental spectrum (**a**) and the simulated spectrum (**b**), both expanded between 294.8 and 304.5 Da highlighting the exact mass and isotopic ratio.

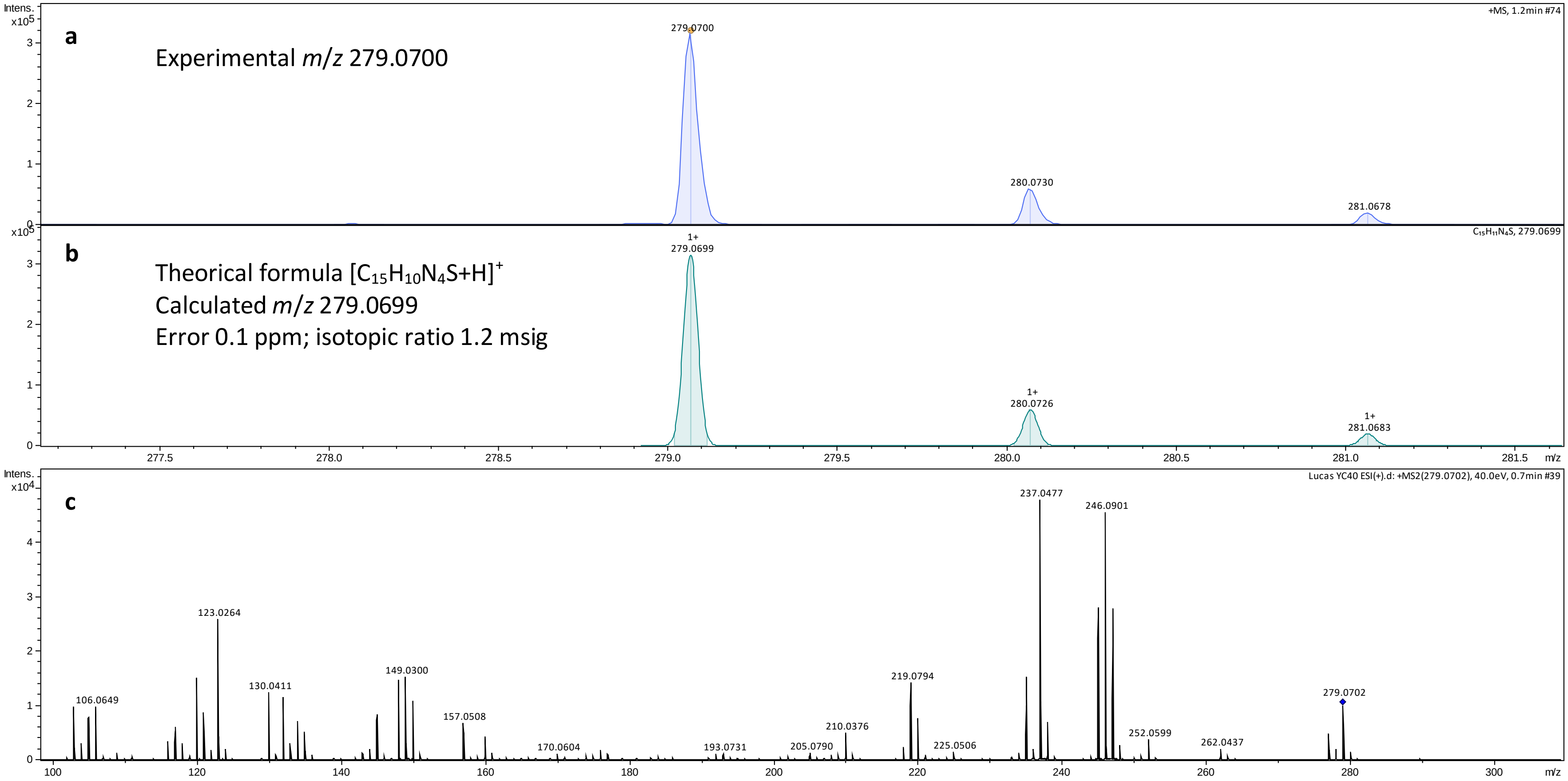

Figure S78. The HRMS analysis of compound **4h**. The experimental spectrum (**a**) and the simulated spectrum (**b**), both expanded between 275.9 and 281.6 Da highlighting the exact mass and isotopic ratio; the analysis in MS–MS mode (**c**) (fragmentation pathway).

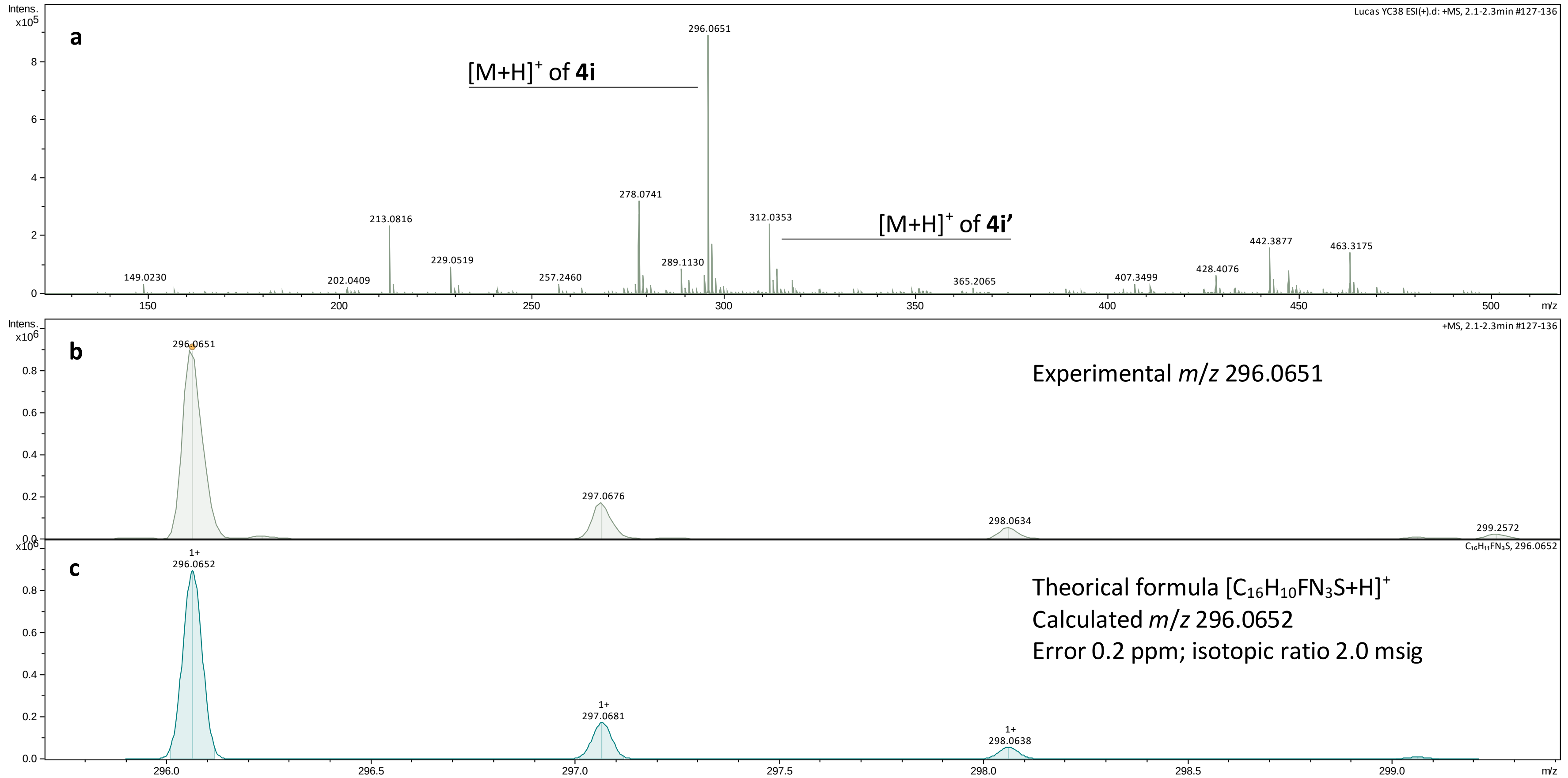

Figure S79. The HRMS analysis of the mixture of compounds **4i** and **4i'**. The full experimental spectrum (**a**); the experimental spectrum (**b**) and the simulated spectrum (**c**), both expanded between 295.6 and 299.8 Da highlighting the exact mass and isotopic ratio.

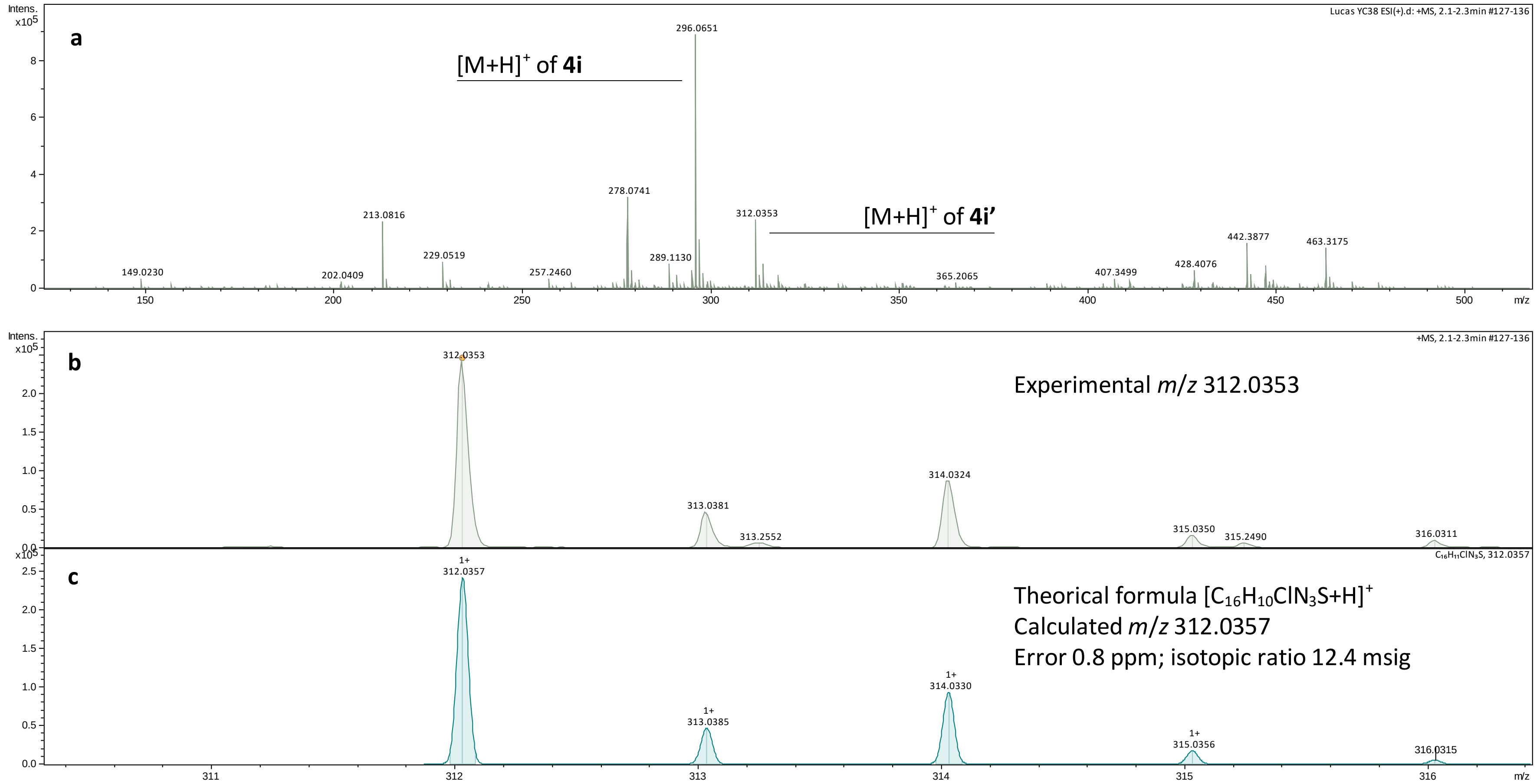

Figure S80. The HRMS analysis of the mixture of compounds **4i** and **4i'**. The full experimental spectrum (**a**); the experimental spectrum (**b**) and the simulated spectrum (**c**), both expanded between 310.3 and 316.4 Da highlighting the exact mass and isotopic ratio.

References

1. Liu, R.; Zhu, Y.; Qin, L.; Ji, S. *Synth. Commun.* **2008**, *38*, 249–254. doi:10.1080/00397910701750250

2. Lebedev, A. Y.; Khartulyari, A. S.; Voskoboynikov, A. Z. *J. Org. Chem.* **2005**, *70*, 596–602. doi:10.1021/jo048671t

3. Gao, M.; Liu, X.; Wang, X.; Cai, Q.; Ding, K. *Chin. J. Chem.* **2011**, *29*, 1199–1204. doi:10.1002/cjoc.201190223

4. Alam, M. S.; Liu, L.; Lee, Y.-E.; Lee, D.-U. *Chem. Pharm. Bull.* **2011**, *59*, 568–573. doi:10.1248/cpb.59.568

5. Maccioni, E.; Cardia, M. C.; Distinto, S.; Bonsignore, L.; De Logu, A. *Farmaco* **2003**, *58*, 951–959. doi:10.1016/S0014-827X(03)00154-X
